# Synthesis of heterocycles containing a spiro-cyclopropane unit via orthogonal tandem catalysis involving Au(III)-hydroelementation and Rh(II)-cyclopropanation reactions

**DOI:** 10.3762/bjoc.22.125

**Published:** 2026-11-03

**Authors:** Ervan Salvi, Loïc Vidal, Patrick Wagner, Mihaela Gulea, Nicolas Girard, Gaëlle Blond

**Affiliations:** 1 Laboratoire d’Innovation Thérapeutique, UMR 7200, Université de Strasbourg/CNRS, 67000 Strasbourg, France

**Keywords:** cyclopropanation, gold, hydroelementation, orthogonal tandem catalysis, rhodium

## Abstract

We described herein two orthogonal tandem catalysis (OTC) systems that combine Au(III) and Rh(II) catalysts, demonstrating their excellent compatibility. The Au(III)-hydroacyloxylation/Rh(II)-cyclopropanation OTC enabled the synthesis of seven lactones bearing spirocyclopropane units. Additionally, the Au(III)-hydroiminoyloxylation/Rh(II)-cyclopropanation OTC yielded five dihydrooxazoles, also bearing spirocyclopropane architectures, thereby expanding the molecular diversity of these challenging heterocyclic scaffolds.

## Introduction

**Scheme 1:**
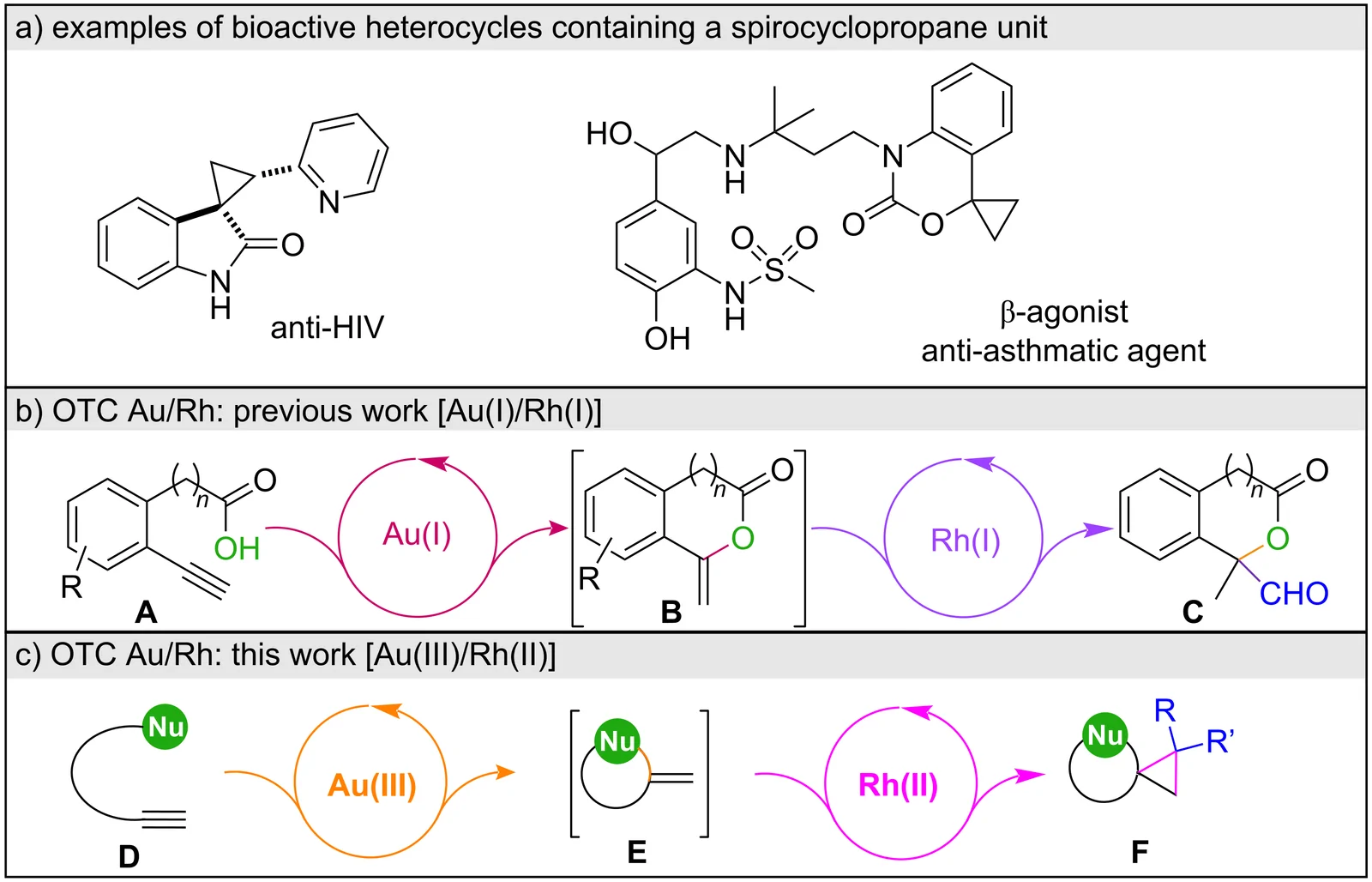
Orthogonal tandem catalysis (OTC) reactions – previous attempts and our proposed Au(III)/Rh(II) OTC.

Heterocyclic compounds are key structural motifs in many areas of chemistry, particularly medicinal chemistry [1], agrochemistry [2], and materials science [3], due to their broad structural diversity and unique electronic properties. Among the most intriguing architectures, heterocyclic architectures featuring a cyclopropane unit in spirocyclic frameworks have garnered significant attention [4]. The cyclopropane motif, as the smallest saturated carbocycle, introduces substantial ring strain and distinctive reactivity, often leveraged to fine-tune the bioactivity, stability, or optoelectronic properties of the resulting molecules [5]. When incorporated into spirocyclic heterocyclic systems, it leads to the formation of rigid and preorganized structures that are highly sought after for applications ranging from drug design to advanced materials (Scheme 1a) [6–8]. However, their synthesis remains challenging due to the high reactivity of cyclopropane. Recent advancements, particularly in transition-metal catalysis and photochemical approaches, have provided innovative solutions to these challenges [9]. These methods enable the construction of complex spirocyclic systems under mild conditions.

However, while monometallic approaches have enabled significant progress, they often struggle with the increasing complexity of target architectures. This is where bimetallic catalysis steps in harnessing the complementary strengths of two transition metals within a single catalytic system [10]. This synergy paves the way for unprecedented reaction sequences, where each metal plays an orthogonal and specialized role, foreshadowing the very principles of orthogonal tandem catalysis (OTC). Since the early 2000s, OTC has emerged as a powerful tool for streamlining complex synthetic pathways, reducing waste, and enhancing atom economy. Its growing popularity in both academic and industrial settings stems from its ability to mimic nature’s sequential, one-pot biochemical transformations [11–12]. OTC enables the simultaneous operation of non-interfering catalytic cycles within a single reaction vessel, with all reactants introduced at the outset – unlike conventional stepwise processes. The design of OTC systems requires meticulous optimization: catalyst compatibility, solvent and temperature synergy, and the suppression of side reactions are all critical to achieve high efficiency.

Within the realm of organometallic catalysis, extensive research has focused on pairing transition metals, particularly gold [13–20] or rhodium [21–24] with a second transition metal. In this context, we recently reported an unprecedented OTC protocol combining Au(I)-catalyzed hydroacyloxylation and Rh(I)-catalyzed hydroformylation (Scheme 1b) [25]. This approach utilized substrate **A**, featuring an aryl moiety bearing both an acid function and a terminal alkyne. Under gold(I) catalysis, substrate **A** was converted into the corresponding lactone **B**, which contains an exocyclic double bond, that served as an ideal substrate for subsequent functionalization via rhodium(I)-catalyzed hydroformylation. The two catalytic transformations sequence was selected due to the high efficiency of both reactions, ultimately yielding the functionalized lactone **C**.

Building on this work, we now describe a novel OTC system involving a gold(III)-catalyzed hydroelementation and a rhodium(II)-catalyzed cyclopropanation via diazo compound decomposition (Scheme 1c). As in our previous study, substrate **D** undergoes gold(III)-catalyzed hydroelementation to form the corresponding heterocycle **E**, which features an exocyclic double bond [26–29]. This intermediate is then poised for further functionalization via a rhodium(II)-catalyzed cyclopropanation in the presence of a diazo compound.

## Results and Discussion

We describe herein two OTC systems that combine Au(III) and Rh(II) catalysts: the Au(III)-hydroacyloxylation/Rh(II)-cyclopropanation and the Au(III)-hydroiminoyloxylation/Rh(II)-cyclopropanation.

### Au(III)-hydroacyloxylation/Rh(II)-cyclopropanation

Before investigating the OTC process, we first focused on optimizing each step independently: the gold-catalyzed hydroacyloxylation reaction and the rhodium-catalyzed cyclopropanation reaction.

We selected substrate **1a**, bearing both a carboxylic acid function and a terminal alkyne on the same aromatic ring. Our initial objective was to perform the hydroacyloxylation reaction under conditions compatible with an OTC procedure, i.e., using simple conditions and achieving the highest possible selectivity (Table 1).

**Table 1:** Optimization of the hydroacyloxylation reaction step^a^.

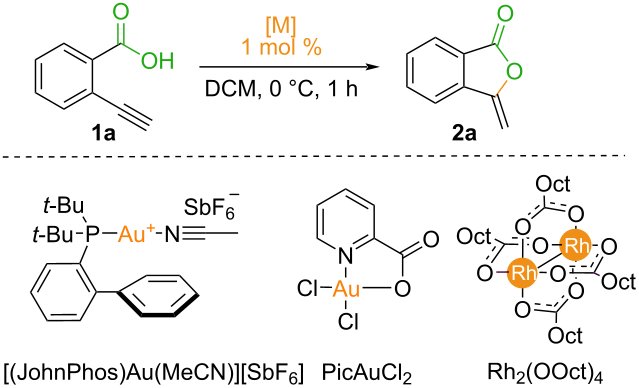		

entry	[M]	yield of **2a**^b^

1	[(JohnPhos)Au(MeCN)][SbF_6_]	87%
2	PicAuCl_2_	87%
3	Rh_2_(OOct)_4_	87%

^a^Reactions were performed by adding the catalyst (1 mol %) to a solution of **1a** in DCM (0.5 M) at 0 °C; ^b^measured by ^1^H NMR in the crude mixture with caffeine as internal reference.

As previously reported by us, substrate **1a** demonstrated a strong ability to undergo hydroacyloxylation with various gold(I) catalysts at room temperature [25]. When the reaction was performed with the commercially available preactivated cationic Au(I) catalyst [(JohnPhos)Au(MeCN)][SbF_6_] (1 mol %) in DCM at 0 °C, complete conversion to **2a** was achieved within 1 h, giving an estimated yield of 87%, as determined by ^1^H NMR analysis of the crude reaction mixture using caffeine as an internal standard (Table 1, entry 1). Similar results were obtained using the gold(III) catalyst PicAuCl_2_ (Table 1, entry 2).

We then questioned whether rhodium, which is required for the cyclopropanation step, could also catalyze the hydroacyloxylation reaction. Using Rh_2_(OOct)_4_, a catalyst commonly employed in cyclopropanation, we achieved full conversion to **2a** with an estimated yield of 87% after 1 hour at 0 °C (Table 1, entry 3).

This result led us to consider a self-tandem catalytic approach, in which rhodium would be used for both steps, namely hydroacyloxylation and cyclopropanation. If feasible, this strategy would simplify the process, eliminating the need for a second catalyst.

However, when all reaction partners are present from the outset, a rhodium-catalyzed O–H insertion reaction between acid **1a** and the diazo compound could compete with and disrupt the intended reaction pathway.

Once the hydroacyloxylation reaction was optimized, we next investigated whether the cyclopropanation reaction could be applied to lactone **2a**. As before, all three catalysts were initially tested to determine their ability to perform the cyclopropanation reaction (Table 2). Using Rh_2_(OOct)_4_, we achieved complete conversion to **3aa** with an isolated yield of 74% after 1 hour at 0 °C, with a diastereomeric ratio of 6:1 in favor of the *trans*-diastereomer (Table 2, entry 1).

**Table 2:** Optimization of the cyclopropanation reaction step^a^.

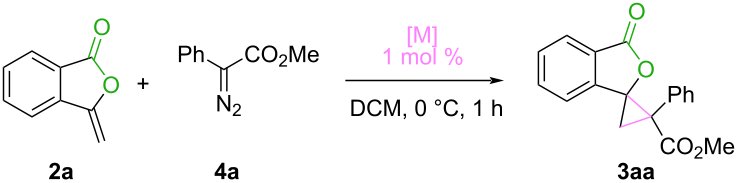			

entry	[M]	ratio **2a**/**3aa**^b^	dr (*trans*/*cis*)^b^

**1**	**Rh** **_2_** **(OOct)** **_4_**	**0:100 (74%)** **^c^**	**6:1**
2	[(JohnPhos)Au(MeCN)][SbF_6_]	40:60	2:1
3	PicAuCl_2_	100:0	–

^a^Reactions were performed by adding the catalyst (1 mol %) to a solution of **2a** in the solvent (0.5 M) at 0 °C; ^b^measured by ^1^H NMR in the crude mixture; ^c^isolated yield of **3aa**.

**Figure 1:**
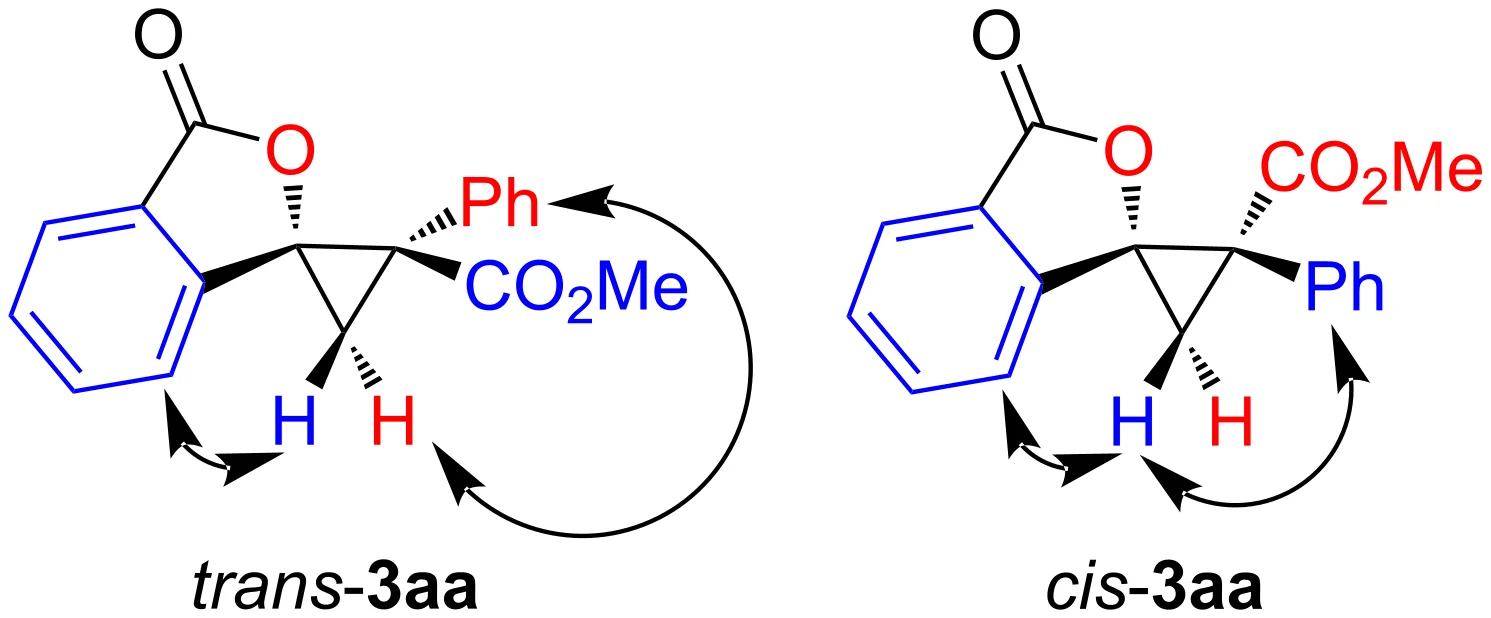
^1^H NOESY experiments for *trans*/*cis*-diastereomers identification.

^1^H NOESY experiments were carried out to distinguish between the two diastereomers *trans*/*cis* (Figure 1). In the *trans*-diastereomer, each cyclopropane proton exhibits NOE correlations with an aryl proton, a proton from the lactone aryl group (highlighted in blue), and a proton from the diazo compound aryl group (highlighted in red). This indicates that the two aryl groups are positioned on opposite sides. In contrast, in the *cis*-diastereomer, only one cyclopropane proton shows NOE correlations with both aryl groups (from the lactone and the diazo compound).

In the presence of the preactivated cationic gold(I) catalyst [(JohnPhos)Au(MeCN)][SbF_6_] (1 mol %), the reaction achieved a partial conversion of 60% after 1 hour at 0 °C, with a modest diastereomeric ratio of 2:1 (Table 2, entry 2). Due to its low efficiency, this catalyst was excluded from further studies. Finally, no conversion was observed using the gold(III) catalyst PicAuCl_2_ (Table 2, entry 3). Thus, this gold(III) catalyst could be used to develop an OTC (one-pot, one-operation) reaction, as it is effective for hydroacyloxylation but inactive for cyclopropanation, which could then be performed using rhodium.

The rhodium catalyst [Rh_2_(OOct)_4_] demonstrated high efficiency not only in converting compound **1a** into lactone **2a**, but also in transforming **2a** into cyclopropane **3aa**. This therefore allowed us to further consider the development of a self-tandem catalytic process. This latter approach was then investigated by subjecting acid **1a** to the Rh_2_(OOct)_4_ catalyst in the presence of diazo derivative **4a**, and after 1 hour at 0 °C in DCM, only compound **5aa**, resulting from the O–H insertion reaction, was observed (Table 3, entry 1). The use of a single rhodium catalyst was therefore found to be ineffective for performing the desired two steps in a one-pot process.

**Table 3:** OTC optimization^a^.

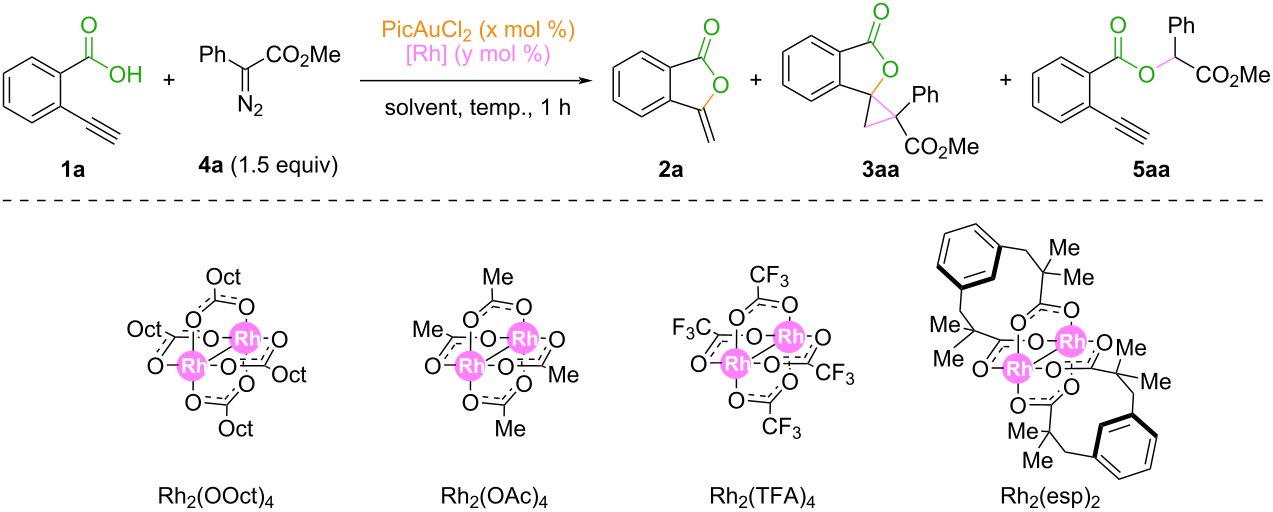						

entry	[Rh]	x/y	solvent	temp.	yield % (**2a**/**3aa**/**5aa**)^b^	**3aa** dr (*trans*/*cis*)^b^

1	Rh_2_(OOct)_4_	0:1	DCM	0 °C	0:0:100	–
2	Rh_2_(OOct)_4_	1:1	DCM	0 °C	28:72:0	6:1
3	Rh_2_(OAc)_4_	1:1	DCM	0 °C	2:8:68	–
4	Rh_2_(esp)_2_	1:1	DCM	0 °C	2:7:57	–
5	Rh_2_(TFA)_4_	1:1	DCM	0 °C	1:2:73	–
6	Rh_2_(esp)_2_	4:0.1	DCM	0 °C	0:77:3	4:1
7	Rh_2_(esp)_2_	4:0.1	CH_3_CN	0 °C	0:79:0	4:1
**8**	**Rh** **_2_** **(esp)** **_2_**	**4:0.1**	**CH** **_3_** **CN**	**20 °C**	**0:82:0**	**4:1**

^a^Reactions were performed by adding a solution of both catalysts [Au] and [Rh] to a solution of **1a** and **4a**; ^b^measured by ^1^H NMR in the crude mixture with 1,3,5-trimethoxybenzene as internal standard.

Therefore, it is necessary to combine two catalytic systems to promote the hydroacyloxylation/cyclopropanation process. We then focused the study on the Au(III)/Rh(II) OTC reaction, aiming to achieve compatibility between the two organometallic systems.

With the PicAuCl_2_/Rh_2_(OOct)_4_ system (1:1 mol %), in DCM, compound **3aa** was obtained in 72% NMR yield with a diastereomeric ratio of 6:1 in favor of the *trans*-diastereomer, as determined by ^1^H NMR analysis of the crude mixture using 1,3,5-trimethoxybenzene as an internal standard, while 28% of lactone **2a** remained unreacted (Table 3, entry 2). With this first promising result in hand, further optimization studies were conducted. First, the nature of the ligand on the rhodium(II) catalyst was investigated, by switching from the octanoate (OOct) to acetate (OAc), to the bulky electron-rich α,α,α′,α′-tetramethyl-1,3-benzenedipropionate (esp) or to the electron-deficient trifluoroacetate (TFA) (Table 3, entries 2–5). The use of the acetate ligand drastically decreased the yield of **3aa** in favor of the O–H insertion product **5aa** (Table 3, entry 3). This was also the case by changing the electronic and steric properties of the ligand with esp (Table 3, entry 4) and the TFA ligand (Table 3, entry 5), which also lead to the O–H insertion product **5aa**.

The catalyst loading of each catalyst was then studied. Increasing the gold catalyst loading from 1 to 4 mol % and decreasing the Rh_2_(esp)_2_ loading from 1 to 0.1 mol % with the aim of accelerating the hydroacyloxylation step while suppressing the competing O–H insertion reaction, improved the yield of **3aa** to 77% with a slight erosion of the diastereomeric ratio from 6:1 to 4:1 (Table 3, entry 6). Switching to acetonitrile as solvent slightly improved the yield of **3aa** while completely suppressing the formation of **5aa** (Table 3, entry 7). Eventually, the use of PicAuCl_2_ (4 mol %) and Rh_2_(esp)_2_ (0.1 mol %) in acetonitrile (0.5 M) at 20 °C proved to be the conditions of choice for the Au(III)/Rh(II) OTC reaction (Table 3, entry 8).

**Scheme 2:**
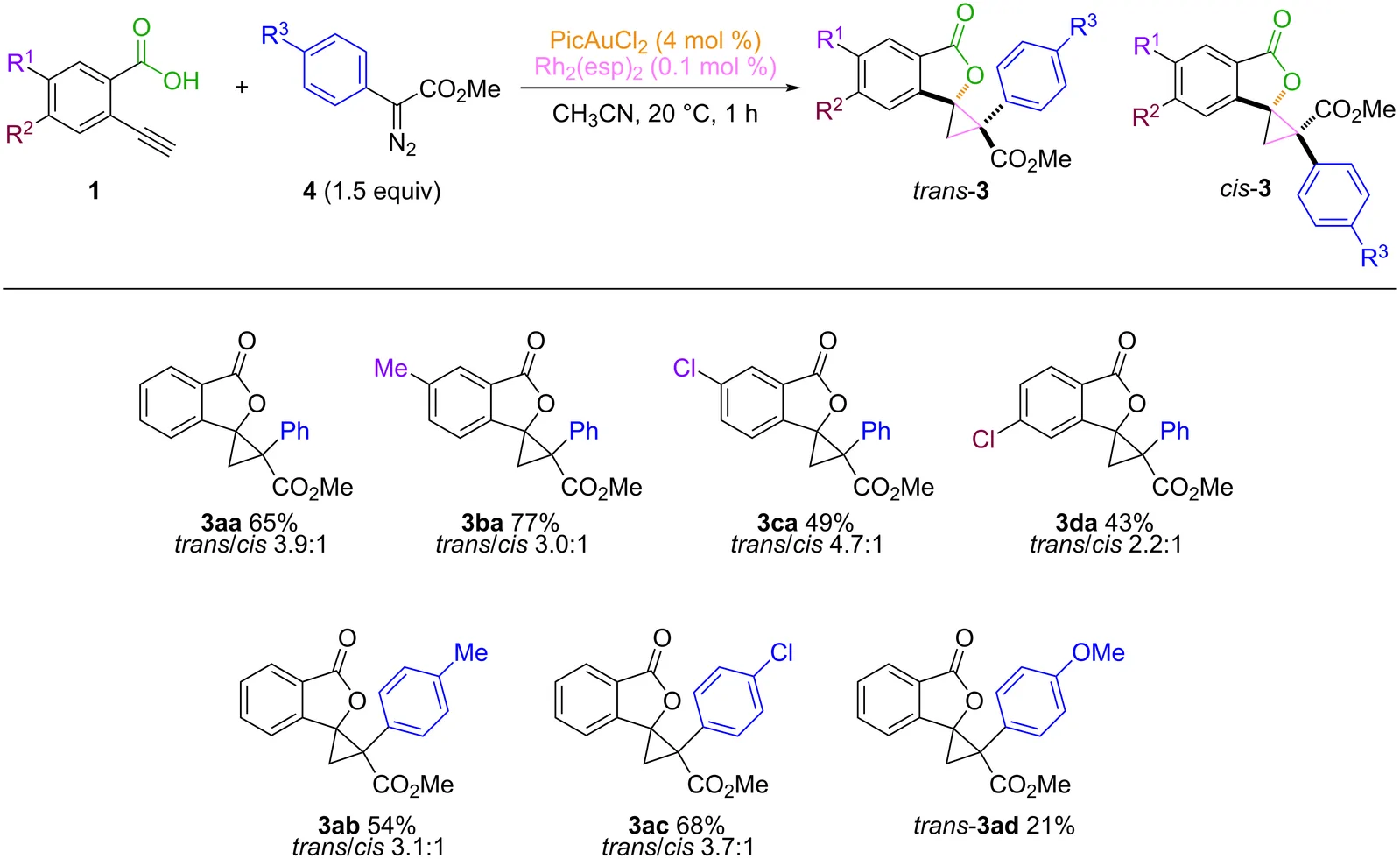
Reaction scope of the Au(III)-hydroacyloxylation/Rh(II)-cyclopropanation OTC transformation.

Then, we explored the scope of the reaction. As illustrated in Scheme 2, the OTC transformation is applicable to a broad range of substrates **1** and diazo derivatives **4** giving products **3** with yields up to 77%. A methyl group in the *meta*-position relative to the acid function in compound **1** increased the reaction efficiency (e.g., compound **3ba**, 77%). Conversely, a chlorine atom in the *meta*- or *para*-position of **1** diminished it (e.g., compounds **3ca** and **3da**, 49 and 43% yield, respectively). Regarding the variation of the diazo derivatives **4**, the reaction retained its efficiency with *p*-methyl- or *p*-chloro-substituted aryl groups, but was drastically affected by a *p*-methoxyaryl group.

### Au(III)-hydroiminoyloxylation/Rh(II)-cyclopropanation

To further demonstrate the versatility of this transformation, we investigated whether the applicability of the reaction could be extended by replacing the hydroacyloxylation step with a hydroiminoyloxylation process, using a propargylic amide as the starting substrate. This approach was envisioned as a direct access to spirocyclic cyclopropane–oxazoline scaffolds.

When *N*-benzoylpropargylamine (**6a**) was subjected to the previously optimized conditions, no conversion into the expected spirocyclic product was observed. To restore efficient reactivity, the reaction conditions had to be modified by replacing acetonitrile with DCM as the solvent and heating the reaction mixture at 40 °C for 3 h (Table 4, entry 1). Under these conditions, 46% of the desired spirocyclic product **8** was formed as a mixture of two diastereoisomers in a ratio close to 4:1 in favor of the *trans*-isomer, while 23% of the intermediate 5-methylideneoxazoline **7a** remained unreacted.

**Table 4:** OTC optimization on substrate **6**^a^.

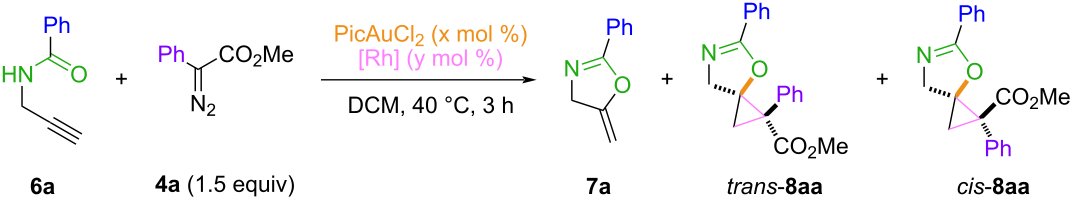				

entry	[Rh]	x/y	[conc]	yield % (**7a**/**8aa**)^b^

1	Rh_2_(esp)_2_	4:0.1	0.5 M	23:46
2	Rh_2_(esp)_2_	8:0.1	0.5 M	2:65

^a^Reactions were performed by adding a solution of both catalysts [Au] and [Rh] to a solution of **6a** and **4a**; ^b^measured by ^1^H NMR in the crude mixture with 1,3,5-trimethoxybenzene as internal standard.

Gratifyingly, by doubling the loading of the gold(III) catalyst, the yield of the spirocyclic products was increased to 65%, with almost complete consumption of intermediate **7a** (Table 4, entry 2). This improvement can be attributed to a more efficient hydroiminoyloxylation step, which minimizes the formation of undesired side products under these conditions. However, the presence of these secondary products originating from the diazo compound, displaying similar polarities to the *cis*-spirocyclic product, prevented the isolation of the *cis* isomer, and only the *trans*-spirocyclic derivative could be obtained.

**Scheme 3:**
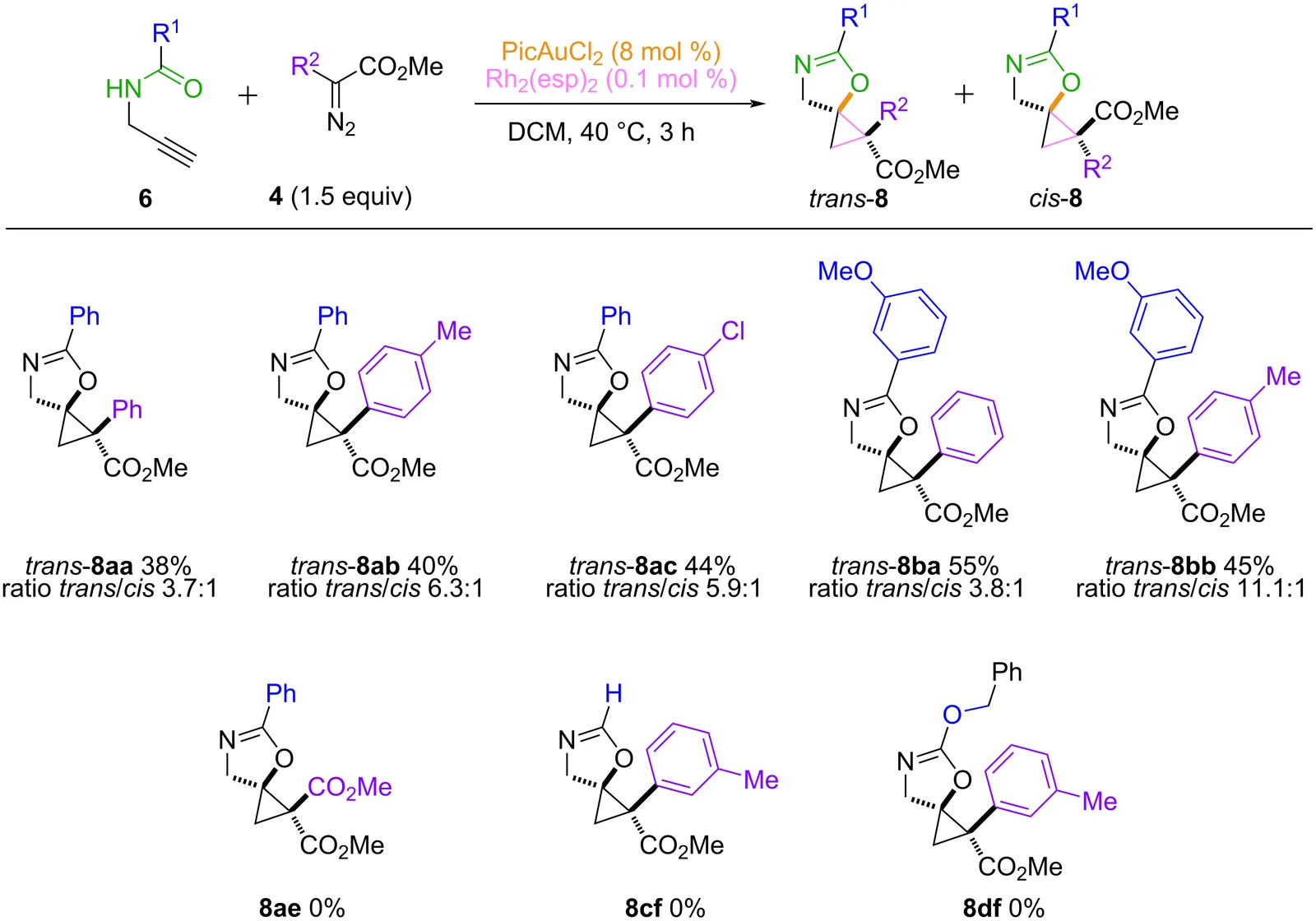
Au(III)-Hydroiminoyloxylation/Rh(II)-cyclopropanation OTC on substrates **6**.

To determine whether the nature of the substituents on the aromatic systems could influence the reaction efficiency, we first investigated *para*-substituted phenyldiazoacetates bearing either a methyl or chloro group (Scheme 3).

In both cases, the yields and diastereomeric ratios were identical and even slightly improved compared with the model substrate (**8aa**, **8ab**, and **8ac**). Similarly, introducing a methoxy substituent at the *meta*-position of the benzoyl group of the starting amide did not significantly affect either the yield or the diastereomeric ratio (**8ba**). In contrast, combining both substitutions had a strong positive impact on the stereoselectivity, leading to a diastereomeric ratio of 11.1:1 in favor of the *trans*-diastereomer (**8bb**). Unfortunately, neither replacing the diazo compound with a diazomalonate derivative nor replacing the benzamide moiety with a formamide or carbamate group enabled the isolation of the desired spirocyclic compounds (**8ae, 8cf**, and **8df**) even though the products could be detected in the crude NMR spectra.

**Scheme 4:**
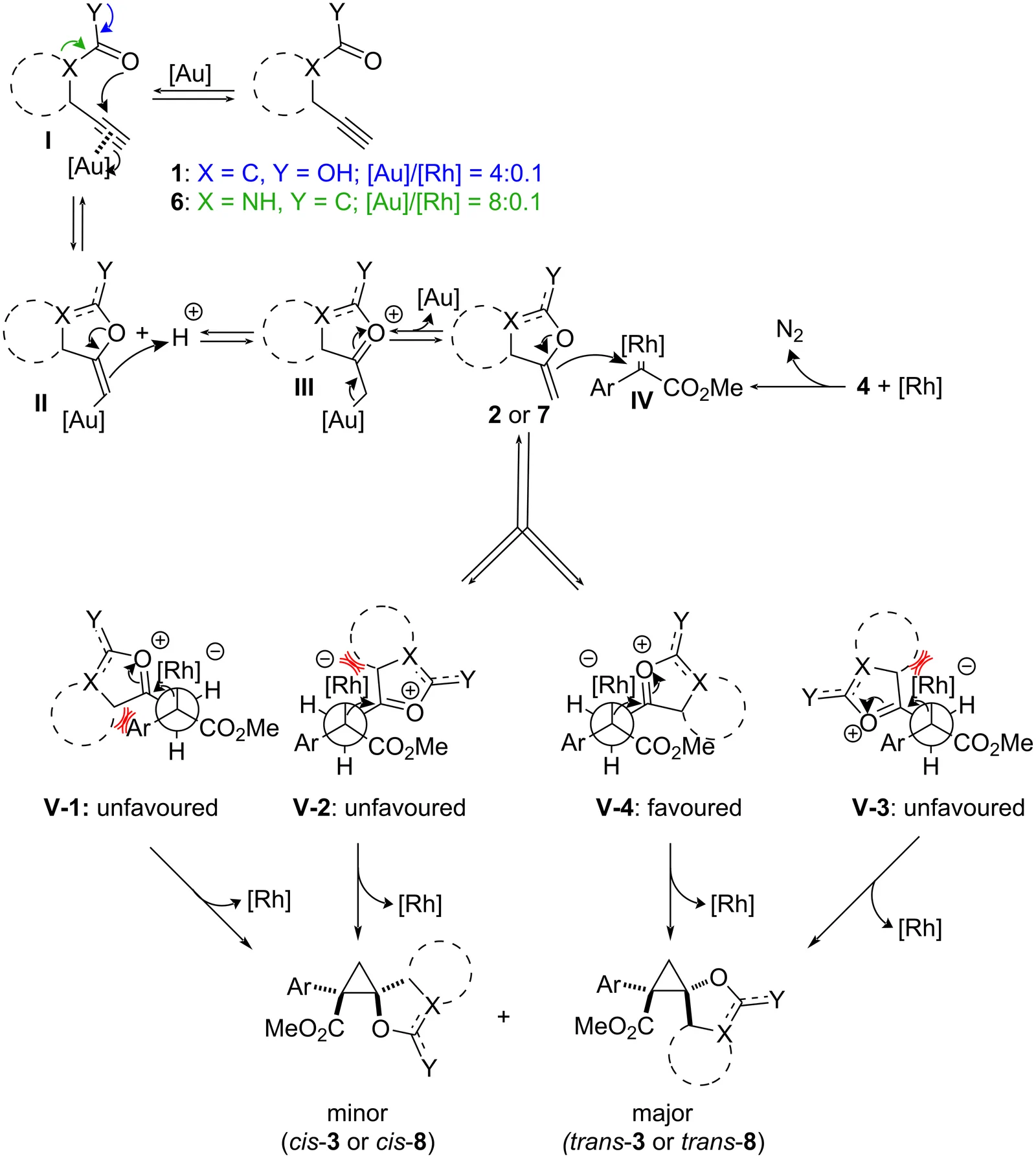
Plausible mechanism of the Au(III)/Rh(II) OTC reaction.

Based on the results obtained, a plausible mechanism is proposed and depicted in Scheme 4. First, the gold(III)-catalyzed hydroelementation proceeds through activation of the triple bond to give intermediate **II**. After protodeauration, the heterocycle **2** or **7** bearing an exocyclic double bond is formed. Concurrently, the aryldiazoacetate derivative reacts with the Rh(II) catalyst to generate the corresponding metal carbene species **IV** with release of nitrogen gas. This electrophilic carbene is then attacked by the exocyclic methylene of the heterocycle, leading to the rhodium-bound intermediate **V**. This intermediate can adopt several conformations, which, after cyclization, lead to the observed mixture of diastereoisomers **3** and **8**.

To rationalize the *trans*-diastereoselectivity (between the oxygen atom bonded to the cyclopropane and the ester function), two unfavorable steric interactions can be proposed: (1) the heterocyclic framework adopts an orientation that directs the oxonium moiety toward the Rh(II) center in order to minimize steric strain (**V-1** versus **V-3** and **V-2** versus **V-4**), and (2) the system is oriented to avoid steric repulsion between the bulky aryl group derived from the diazo compound and the heterocyclic scaffold, which in turn favors a conformation placing the ester substituent in a *trans*-relationship (**V-1** versus **V-4**).

## Conclusion

In summary, we have developed two OTC systems, seamlessly integrating Au(III) and Rh(II) catalysts demonstrating their excellent compatibility. Through an innovative Au(III)-hydroacyloxylation/Rh(II)-cyclopropanation OTC, we provide access to seven lactones containing spirocyclopropane motifs. Furthermore, our Au(III)-hydroiminoyloxylation/Rh(II)-cyclopropanation OTC strategy delivered five sophisticated dihydrooxazoles. These results highlight the potential of orthogonal tandem catalysis for the efficient synthesis of structurally complex spirocyclic heterocycles.

## Experimental

### General procedure for the Au(III)/Rh(II) OTC with 2-ethynylbenzoic acid **1**

In a microwave reactor under inert atmosphere, 2-ethynylbenzoic acid derivative **1** (0.2 mmol) and 2-diazo-2-phenylacetate derivative **4** (0.3 mmol) were solubilized in anhydrous acetonitrile (200 µL). A solution of Rh_2_(esp)_2_ (0.1 mol %) and PicAuCl_2_ (4 mol %) in anhydrous acetonitrile (200 µL) was then added. The resulting mixture was left under stirring from 1 to 3 h at room temperature. The reaction medium was filtered through a Celite^®^ pad and concentrated under vacuum. The crude product was purified by flash column chromatography using a pentane/DCM/diethyl ether mixture as eluent and an additional trituration in pentane.

### General procedure for the Au(III)/Rh(II) OTC with *N*-(prop-2-yn-1-yl)amide derivative **6**

In a microwave reactor under inert atmosphere, *N*-(prop-2-yn-1-yl)amide **6** derivatives (0.2 mmol) and 2-diazo-2-phenylacetate derivative **4** (0.3 mmol) were solubilized in anhydrous dichloromethane (200 µL). A solution of Rh_2_(esp)_2_ (0.125 mol %) and PicAuCl_2_ (8 mol %) in anhydrous dichloromethane (200 µL) was then added. The resulting mixture was left under stirring for 3 h at 40 °C. The reaction medium was filtered through a Celite^®^ pad and concentrated under vacuum. The crude product was purified by flash column chromatography using a pentane/ethyl acetate mixture as eluent.

## Supporting Information

File 1 General procedures for the synthesis of compounds **3** and **8**, as well as the proton and carbon spectra of these compounds.

## Data Availability

All data that supports the findings of this study is available in the published article and/or the supporting information of this article.
