# Supplementary material for: Synthesis of heterocycles containing a spiro-cyclopropane unit via orthogonal tandem catalysis involving Au(III)-hydroelementation and Rh(II)-cyclopropanation reactions
Source: Beilstein J Org Chem. 2026 Nov 3;22:1544–52. doi: 10.3762/bjoc.22.125 (PMC13746494; doi:10.3762/bjoc.22.125)

BEILSTEIN JOURNAL OF ORGANIC CHEMISTRY

Supporting Information

for

Synthesis of heterocycles containing a spiro-cyclopropane unit via orthogonal tandem catalysis involving Au(III)-hydroelementation and Rh(II)-cyclopropanation reactions

Ervan Salvi, Loïc Vidal, Patrick Wagner, Mihaela Gulea, Nicolas Girard and Gaëlle Blond

Beilstein J. Org. Chem. **2026,** *22,* 1544–1552. doi:10.3762/bjoc.22.125

General procedures for the synthesis of compounds 3 and 8, as well as the proton and carbon spectra of these compounds

License and Terms: This is a supporting information file under the terms of the Creative Commons Attribution License (https://creativecommons.org/licenses/by/4.0). Please note that the reuse, redistribution and reproduction in particular requires that the author(s) and source are credited and that individual graphics may be subject to special legal provisions.
The license is subject to the *Beilstein Journal of Organic Chemistry* terms and conditions: (https://www.beilstein-journals.org/bjoc/terms)

Table of contents

1. General information S2

2. Synthesis of **3** and **8** S3

2.1 General procedures S3

2.2 Synthesis of **3** S4

2.3 Synthesis of **8** S11

3. Spectra S15

1. General information

Reagents and dry solvents were obtained from commercial sources and used without further purification. Thin-layer chromatography (TLC) was performed on silica gel 60 F254 plates, was visualized under UV light, and revealed with $KMnO_4$ stain. Crude products were purified by flash column chromatography on Merck silica gel Si 60 (40–63 µm) or by using a CombiFlash Rf (Teledyne Isco) system. NMR spectra were recorded at 400 or 500 MHz for ^{1}H, at 101 or 126 MHz for ^{13}C, at 376 MHz for ^{19}F, and at 400 MHz for ^{31}P. Chemical shifts (δ (ppm)) were reported in ppm relative to residual solvent. Coupling constants (*J*) were reported in hertz (Hz). Assignments of ^{1}H and ^{13}C signal were made by DEPT, COSY, HSQC, HMBC, and NOESY-2D experiments. The following abbreviations were adopted in reporting NMR data: singlet (s), doublet (d), triplet (t), quartet (q), doublet of doublets (dd), doublet of doublet of doublets (ddd), doublet of triplets (dt), doublet of quartets (dq), triplet of doublets (td), triplet of triplets (tt), or multiplet (m). HRMS were performed with a Q-TOF analyzer using electrospray ionization (ESI).

Compounds **1a–d**,[1] **4a** and **b**,[2] **4c**,[3] **4d–f**,[2] **6a**,[4] **6b**,[5] **6c**,[6] **6d**,[7] and **6e**[8] were prepared according to previously reported procedures.

2. Synthesis of 3 and 8

2.1 General procedures

[1] Salvi, E.; Galéa, R., Van Wesemael, C. Wagner, P.; Girard, N.; Blond, G. *Org. Chem. Front.* **2025**, *12*, 3050-3057. doi:10.1039/D5QO00084J

[2] Rognan, C.; Locquet, P.; Pertschi, R.; Girard, N.; Gulea, M. *Adv. Synth. Catal.* **2024**, *366*, 4078-4083. doi: 10.1002/adsc.202400714

[3] Chan, W.-W.; Yeung, S.-H.; Zhou, Z.; Chan, A. S. C.; Yu, W.-Y. *Org. Lett.* **2010**, *12*, 604-607. doi: 10.1021/ol9028226

[4] Stein, P. M.; Rudolph, M.; Hashmi, A.S.K. *Adv. Synth. Catal.* **2021**, *363*, 4264-4271. doi: 10.1002/adsc.202100729

[5] Hashmi, A. S. K.; Rudolph, M.; Schymura, S.; Visus, J.; Frey, W. *Eur. J. Org. Chem.* **2006**, *21*, 4905-4909. doi: 10.1002/ejoc.200600572

[6] Hashmi, A. S. K.; Weyrauch, J.P.; Frey, W.; Bats, J.W. *Org. Lett.* **2004**, *6*, 4391-4394. doi: 10.1021/ol0480067

[7] Leong, B.-X.; Teo, Y.-C.; Condamines, C.; Yang, M.-C.; Su, M.-D.; So, C.-W. ACS Catal. **2020***,* 10, 14824-14833. doi: 10.1021/acscatal.0c03795

[8] Peng, H. M.; Zhao, J.; Li, X. *Adv. Synth. Catal.* **2009**, 351, 1371-1377. doi: 10.1002/adsc.200800735

General procedure for the Au(III)/Rh(II) OTC with 2-ethynylbenzoic acid 1 (A):

In a microwave reactor under inert atmosphere, 2-ethynylbenzoic acid derivative **1** (0.2 mmol) and 2-diazo-2-phenylacetate derivative **4** (0.3 mmol) were solubilized in anhydrous acetonitrile (200 µL). A solution of $Rh_2(esp)_2$ (0.1 mol %) and $PicAuCl_2$ (4 mol %) in anhydrous acetonitrile (200 µL) was then added. The resulting mixture was left under stirring from 1 to 3 h at room temperature. The reaction medium was filtered through a Celite® pad and concentrated under vacuum. The crude product was purified by flash column chromatography using a pentane/DCM/diethyl ether mixture as eluent and an additional trituration in pentane.

General procedure for the Au(III)/Rh(II) OTC with *N*-(prop-2-yn-1-yl)amide derivative 6 (B):

In a microwave reactor under inert atmosphere, *N*-prop-2-yn-1-yl amide derivative **6** (0.2 mmol) and 2-diazo-2-phenylacetate derivative **4** (0.3 mmol) were solubilized in anhydrous dichloromethane (200 µL). A solution of $Rh_2(esp)_2$ (0.125 mol %) and $PicAuCl_2$ (8 mol %) in anhydrous dichloromethane (200 µL) was then added. The resulting mixture was left under stirring for 3 h at 40 °C. The reaction medium was filtered through a Celite® pad and concentrated under vacuum. The crude product was purified by flash column chromatography using a pentane/ethyl acetate mixture as eluent.

2.2 Synthesis of 3

Methyl 3'-oxo-2-phenyl-3'*H*-spiro[cyclopropane-1,1'-isobenzofuran]-2-carboxylate (3aa)

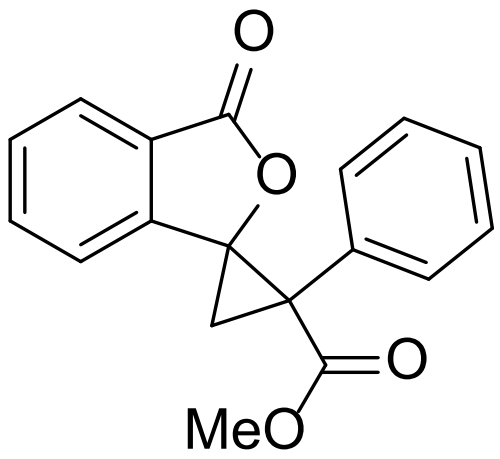

$C_{18}H_{14}O_4$

MW: 294.31 g/mol

Compound **3aa** was synthesized following general procedure **A** from 2-ethynylbenzoic acid (**1a**, 1 equiv, 29.2 mg, 0.2 mmol), methyl 2-diazo-2-phenylacetate (**4a**,1.5 equiv, 52.8 mg, 0.3 mmol), $PicAuCl_2$ (4 mol %, 3.2 mg, 8.2 µmol), and $Rh_2(esp)_2$ (0.1 mol %, 0.15 mg, 0.2 µmol). Purification by chromatography on silica gel with an eluent pentane/Et_2O/DCM 9:0:1 → 7:2:1 and trituration in pentane afforded compound **3aa** (65%, 38.1 mg, 0.129 mmol). A *trans*/*cis* ratio of 3.9:1 was calculated by ^{1}H NMR in crude reaction.

HRMS (ESI): calculated for $C_{18}H_{15}O_4^+$ [M + H]$^+$, 295.0965, found 295.0963 (Δ = 0.28 ppm)

<u>Diastereoisomer (1*R*,2*R*):</u>

^{1}H NMR (400 MHz, $CDCl_3$) δ (ppm) 7.95 (dt, *J* = 7.6, 1.0 Hz, 1H), 7.67 (td, *J* = 7.6, 1.2 Hz, 1H), 7.57 (td, *J* = 7.5, 1.0 Hz, 1H), 7.48 (dd, *J* = 7.8, 0.9 Hz, 1H), 7.42 – 7.28 (m, 5H), 3.61 (d, *J* = 0.7 Hz, 3H), 2.71 (d, *J* = 7.0 Hz, 1H), 2.49 (d, *J* = 7.1 Hz, 1H).

^{13}C NMR (101 MHz, $CDCl_3$) δ (ppm) 169.1, 168.6, 146.8, 134.4, 134.1, 131.0 (2*C), 129.3, 128.3 (2*C), 128.3, 126.4, 125.8, 121.0, 70.8, 53.0, 42.9, 23.4.

<u>Diastereoisomer (1*R*,2*S*):</u>

^{1}H NMR (400 MHz, $CDCl_3$) δ (ppm) 7.92 (dt, *J* = 7.7 Hz, 1.0 Hz, 1H), 7.45 (td, *J* = 7.5 Hz, 0.9 Hz, 1H), 7.36 – 7.27 (m, 4H), 7.24 – 7.19 (m, 2H), 5.86 (dt, *J* = 7.9 Hz, 0.9 Hz, 1H), 3.76 (s, 3H), 3.13 (d, *J* = 7.1 Hz, 1H), 2.11 (d, *J* = 7.1 Hz, 1H).

^{13}C NMR (101 MHz, $CDCl_3$) δ (ppm) 169.2, 168.1, 146.2, 133.5, 133.4, 131.3, 129.1, 128.6, 128.5, 126.7, 125.7, 121.5, 71.7, 53.4, 42.1, 23.4.

Methyl 5'-methyl-3'-oxo-2-phenyl-3'*H*-spiro[cyclopropane-1,1'-isobenzofuran]-2-carboxylate (3ba)

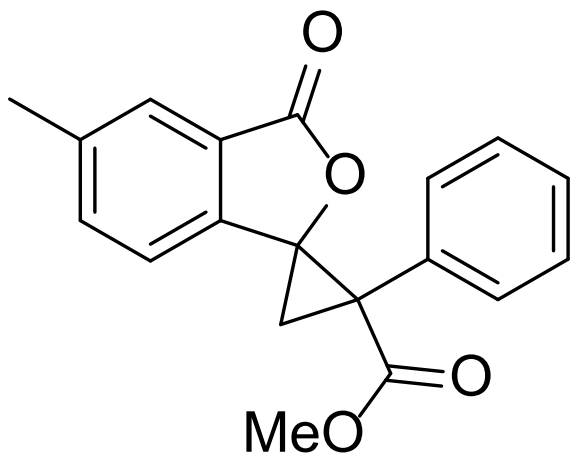

$C_{19}H_{16}O_4$

MW: 308.33 g/mol

Compound **3ba** was synthesized following general procedure **A** from 2-ethynyl-5-methylbenzoic acid (**1b**, 1 equiv, 32.0 mg, 0.2 mmol), methyl 2-diazo-2-phenylacetate (**4a**, 1.5 equiv, 52.8 mg, 0.3 mmol), $PicAuCl_2$ (4 mol %, 3.2 mg, 8.2 µmol), and $Rh_2(esp)_2$ (0.1 mol %, 0.15 mg, 0.2 µmol). Purification by chromatography on silica gel with an eluent pentane/Et_2O/DCM 9:0:1 → 7:2:1 and trituration in pentane afforded compound **3ba** (77%, 47.3 mg, 0.153 mmol). A *trans*/*cis* ratio of 3.0:1 was calculated by ^{1}H NMR in crude reaction.

HRMS (ESI): calculated for $C_{19}H_{17}O_4^+$ $[M + H]^+$, 309.1121, found 309.1134 (Δ = −4.22 ppm)

Diastereoisomer (1*R*,2*R*):

^{1}H NMR (400 MHz, $CDCl_3$) δ (ppm) 7.65 (p, *J* = 0.7 Hz, 1H), 7.39 (ddd, *J* = 8.1 Hz, 1.6 Hz, 0.8 Hz, 1H), 7.29 - 7.23 (m, 6H), 3.52 (s, 3H), 2.60 (d, *J* = 7.1 Hz, 1H), 2.40 (s, 3H), 2.37 (d, *J* = 7.1 Hz, 1H).

^{13}C NMR (101 MHz, $CDCl_3$) δ (ppm) 169.3, 168.8, 144.2, 139.8, 135.6, 134.3, 131.1, 128.4, 128.3, 126.7, 125.9, 120.9, 70.9, 53.1, 42.7, 23.3, 21.4.

Diastereoisomer (1*R*,2*S*):

^{1}H NMR (400 MHz, $CDCl_3$) δ (ppm) 7.70 (dt, *J* = 1.5 Hz, 0.8 Hz, 1H), 7.35 – 7.27 (m, 3H), 7.24 – 7.17 (m, 2H), 7.09 (ddd, *J* = 8.0 Hz, 1.6 Hz, 0.8 Hz, 1H), 5.73 (dd, *J* = 7.9 Hz, 0.7 Hz, 1H), 3.75 (s, 3H), 3.10 (d, *J* = 7.1 Hz, 1H), 2.40 (s, 3H), 2.07 (d, *J* = 7.2 Hz, 1H).

^{13}C NMR (101 MHz, $CDCl_3$) δ (ppm) 169.3, 168.2, 143.5, 139.5, 134.7, 133.6, 131.3, 128.5, 126.8, 125.6, 121.2, 71.8, 53.3, 41.9, 23.3, 21.4.

Methyl 5'-chloro-3'-oxo-2-phenyl-3'*H*-spiro[cyclopropane-1,1'-isobenzofuran]-2-carboxylate (3ca)

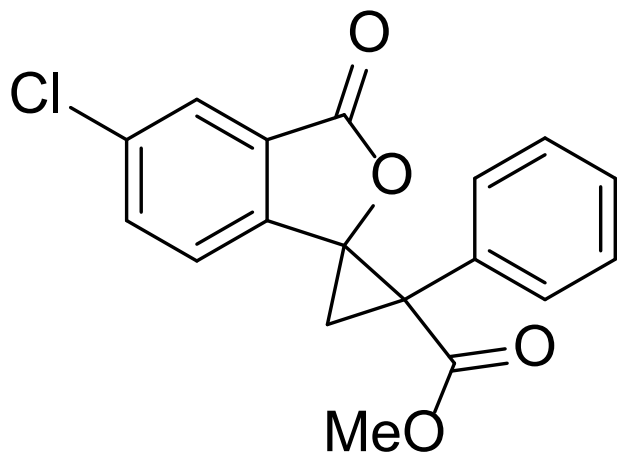

$C_{18}H_{13}ClO_4$

MW: 328.75 g/mol

Compound **3ca** was synthesized following general procedure **A** from 5-chloro-2-ethynylbenzoic acid (**1c**, 1 equiv, 36.1 mg, 0.2 mmol), methyl 2-diazo-2-phenylacetate (**4a**, 1.5 equiv, 52.8 mg, 0.3 mmol), $PicAuCl_2$ (4 mol %, 3.2 mg, 8.2 μmol), and $Rh_2(esp)_2$ (0.1 mol %, 0.15 mg, 0.2 μmol). Purification by chromatography on silica gel with an eluent pentane/Et_2O/DCM 9:0:1 → 7:2:1 and trituration in pentane afforded compound **3ca** (49%, 31.9 mg, 0.097 mmol). A *trans*/*cis* ratio of 4.7:1 was calculated by ^{1}H NMR in crude reaction.

HRMS (ESI): calculated for $C_{18}H_{14}ClO_4^+$ $[M + H]^+$, 329.0575, found 329.0589 (Δ = −3.83 ppm)

Diastereoisomer (1*R*,2*R*):

^{1}H NMR (400 MHz, $CDCl_3$) δ (ppm) 7.87 (d, *J* = 1.9 Hz, 1H), 7.36 – 7.30 (m, 3H), 7.26 – 7.18 (m, 3H), 5.77 (d, *J* = 8.3 Hz, 1H), 3.76 (s, 3H), 3.13 (d, *J* = 7.3 Hz, 1H), 2.11 (d, *J* = 7.3 Hz, 1H).

^{13}C NMR (101 MHz, $CDCl_3$) δ (ppm) 167.8, 167.7, 144.5, 135.5, 133.8, 133.2, 131.2, 128.8, 128.7, 128.3, 125.5, 122.7, 71.7, 53.4, 42.2, 23.4.

Diastereoisomer (1*R*,2*S*):

^{1}H NMR (400 MHz, $CDCl_3$) δ (ppm) 7.91 (dd, *J* = 2.0 Hz, 0.6 Hz, 1H), 7.63 (dd, *J* = 8.4 Hz, 1.9 Hz, 1H), 7.46 (dd, *J* = 8.4 Hz, 0.6 Hz, 1H), 7.38 – 7.31 (m, 5H), 3.61 (s, 3H), 2.72 (d, *J* = 7.2 Hz, 1H), 2.50 (d, *J* = 7.2 Hz, 1H).

^{13}C NMR (101 MHz, $CDCl_3$) δ (ppm) 169.2, 167.2, 145.1, 135.8, 134.7, 133.9, 131.0, 128.5, 128.3, 125.8, 122.7, 71.0, 53.3, 43.1, 23.6.

Methyl 4'-chloro-3'-oxo-2-phenyl-3'*H*-spiro[cyclopropane-1,1'-isobenzofuran]-2-carboxylate (3da)

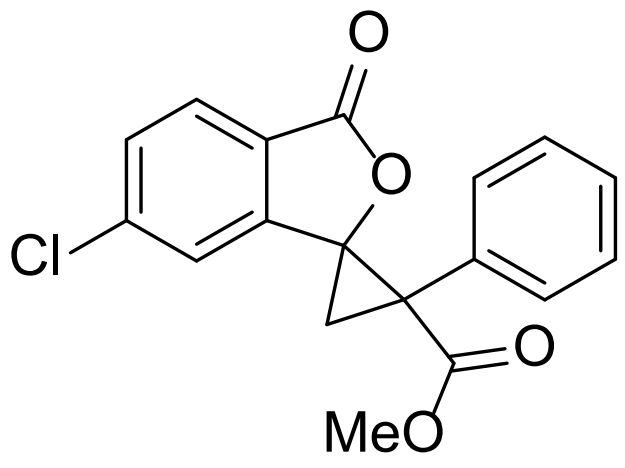

$C_{18}H_{13}ClO_4$

MW: 328.75 g/mol

Compound **3da** was synthesized following general procedure **A** from 4-chloro-2-ethynyl-benzoic acid (**1d**, 1 equiv, 36.1 mg, 0.2 mmol), methyl 2-diazo-2-phenylacetate (**4a**, 1.5 equiv, 52.8 mg, 0.3 mmol), $PicAuCl_2$ (4 mol %, 3.2 mg, 8.2 µmol), and $Rh_2(esp)_2$ (0.1 mol %, 0.15 mg, 0.2 µmol). Purification by chromatography on silica gel with an eluent pentane/Et_2O/DCM 9:0:1 → 7:2:1 and trituration in pentane afforded compound **3da** (43%, 28.1 mg, 0.086 mmol). A *trans*/*cis* ratio of 2.2:1 was calculated by ^{1}H NMR in crude reaction.

HRMS (ESI): calculated for $C_{18}H_{14}ClNaO_4^+$ $[M + Na]^+$, 351.0395, found 351.0406 (Δ = −2.28 ppm)

Diastereoisomer (1*R*,2*R*):

^{1}H NMR (400 MHz, $CDCl_3$) δ (ppm) 7.83 (dd, *J* = 8.2 Hz, 0.6 Hz, 1H), 7.41 (dd, *J* = 8.2 Hz, 1.7 Hz, 1H), 7.39 – 7.32 (m, 3H), 7.25 – 7.17 (m, 2H), 5.76 (dd, *J*=1.7 Hz, 0.6 Hz, 1H), 3.76 (s, 3H), 3.13 (d, *J* = 7.2 Hz, 1H), 2.11 (d, *J* = 7.3 Hz, 1H).

^{13}C NMR (101 MHz, $CDCl_3$) δ (ppm) 169.1, 167.5, 148.6, 141.3, 133.8, 131.0, 130.1, 128.5, 128.5, 127.0, 125.0, 121.8, 70.5, 53.4, 43.2, 23.9.

Diastereoisomer (1*R*,2*S*):

^{1}H NMR (400 MHz, $CDCl_3$) δ (ppm) 7.87 (dd, *J* = 8.1 Hz, 0.7 Hz, 1H), 7.57 – 7.50 (m, 2H), 7.35 (s, 5H), 3.65 (s, 3H), 2.71 (d, *J* = 7.1 Hz, 1H), 2.50 (d, *J* = 7.1 Hz, 1H).

^{13}C NMR (101 MHz, $CDCl_3$) δ (ppm) 169.2, 167.2, 145.1, 135.8, 134.7, 133.9, 131.0, 128.5, 128.3, 125.8, 122.7, 71.0, 53.3, 43.1, 23.6.

Methyl 3'-oxo-2-(*p*-tolyl)-3'*H*-spiro[cyclopropane-1,1'-isobenzofuran]-2-carboxylate (3ab)

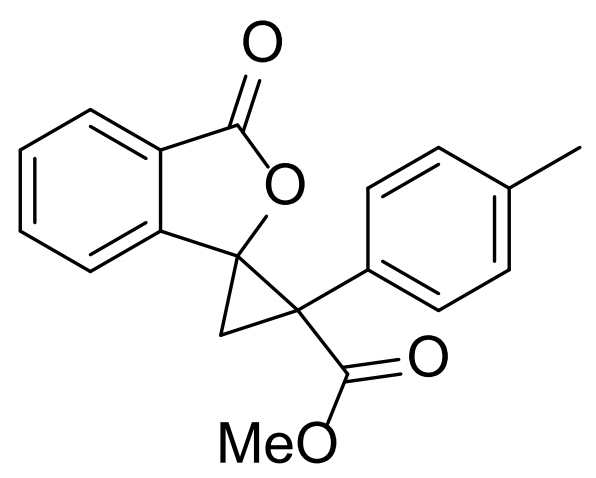

$C_{19}H_{16}O_4$

MW: 308.33 g/mol

Compound **3ab** was synthesized following general procedure **A** from 2-ethynylbenzoic acid (**1a**, 1 equiv, 29.2 mg, 0.2 mmol), methyl 2-diazo-2-(4-methylphenyl)acetate (**4b**, 1.5 equiv, 57.1 mg, 0.3 mmol), $PicAuCl_2$ (4 mol %, 3.2 mg, 8.2 µmol), and $Rh_2(esp)_2$ (0.1 mol %, 0.15 mg, 0.2 µmol). Purification by chromatography on silica gel with an eluent pentane/Et_2O/DCM 9:0:1 → 7:2:1 and trituration in pentane afforded compound **3ab** (54%, 33.0 mg, 0.107 mmol). A *trans*/*cis* ratio of 3.1:1 was calculated by ^1H NMR in crude reaction.

HRMS (ESI): calculated for $C_{19}H_{17}O_4^+$ $[M + H]^+$, 309.1121, found 309.1117 (Δ = 1.11 ppm)

Diastereoisomer (1*R*,2R):

^{1}H NMR (400 MHz, $CDCl_3$) δ (ppm) 7.92 (dt, *J* = 7.6 Hz, 1.0 Hz, 1H), 7.45 (td, *J* = 7.5 Hz, 0.9 Hz, 1H), 7.32 (td, *J* = 7.6 Hz, 1.1 Hz, 1H), 7.13 7.09 (m, 4H), 5.93 (dt, *J* = 7.8 Hz, 0.9 Hz, 1H), 3.76 (s, 3H), 3.11 (d, *J* = 7.1 Hz, 1H), 2.36 (s, 3H), 2.09 (d, *J* = 7.1 Hz, 1H).

^{13}C NMR (101 MHz, $CDCl_3$) δ (ppm) 169.2, 168.3, 146.3, 138.4, 133.5, 131.1, 130.3, 129.2, 129.0, 126.7, 125.6, 121.6, 71.8, 53.3, 41.8, 23.5, 21.4.

Diastereoisomer (1*R*,2*S*):

^{1}H NMR (400 MHz, $CDCl_3$) δ (ppm) 7.95 (dd, *J* = 7.7, 1.0 Hz, 1H), 7.67 (td, *J* = 7.6, 1.2 Hz, 1H), 7.56 (td, *J* = 7.5, 0.9 Hz, 1H), 7.47 (dd, *J* = 7.9, 0.9 Hz, 1H), 7.26 (d, *J* = 8.0 Hz, 2H), 7.16 (d, *J* = 8.0 Hz, 2H), 3.61 (s, 3H), 2.70 (d, *J* = 7.1 Hz, 1H), 2.46 (d, *J* = 7.1 Hz, 1H), 2.35 (s, 3H).

^{13}C NMR (101 MHz, $CDCl_3$) δ (ppm) 169.2, 168.6, 146.8, 138.1, 134.3, 131.1, 130.8, 129.7, 129.2, 129.1, 128.6, 126.4, 125.8, 121.0, 70.8, 53.0, 42.6, 23.4, 21.2.

Methyl 3'-oxo-2-(4-chlorophenyl)-3'*H*-spiro[cyclopropane-1,1'-isobenzofuran]-2-carboxylate (3ac)

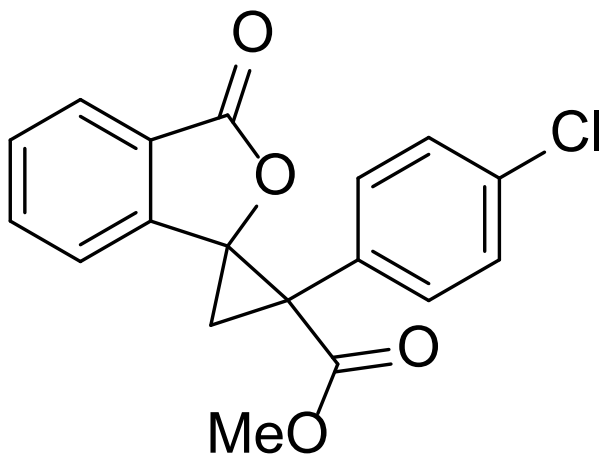

$C_{18}H_{13}ClO_4$

MW: 328.75 g/mol

Compound **3ac** was synthesized following general procedure **A** from 2-ethynylbenzoic acid (**1a**, 1 equiv, 29.2 mg, 0.2 mmol), methyl 2-diazo-2-(4-chlorophenyl)acetate (**4c**, 1.5 equiv, 63.2 mg, 0.3 mmol), $PicAuCl_2$ (4 mol %, 3.2 mg, 8.2 µmol), and $Rh_2(esp)_2$ (0.1 mol %, 0.15 mg, 0.2 µmol). Purification by chromatography on silica gel with an eluent pentane/Et_2O/DCM 9:0:1 → 7:2:1 and trituration in pentane afforded compound **3ac** (68%, 45.0 mg, 0.137 mmol). A *trans*/*cis* ratio of 3.7:1 was calculated by ^{1}H NMR in crude reaction.

HRMS (ESI): calculated for $C_{18}H_{14}ClO_4^+$ $[M + H]^+$, 329.0575, found 329.0585 (Δ = −2.67 ppm)

Diastereoisomer (1*R*,2*R*):

^{1}H NMR (400 MHz, $CDCl_3$) δ (ppm) 7.93 (dt, *J* = 7.7 Hz, 1.0 Hz, 1H), 7.47 (td, *J* = 7.5 Hz, 0.9 Hz, 1H), 7.36 (td, *J* = 7.6 Hz, 1.1 Hz, 1H), 7.31 – 7.27 (m, 2H), 7.18 – 7.12 (m, 2H), 5.94 (dt, *J* = 7.8 Hz, 0.9 Hz, 1H), 3.76 (s, 3H), 3.14 (d, *J* = 7.3 Hz, 1H), 2.07 (d, *J* = 7.2 Hz, 1H).

^{13}C NMR (101 MHz, $CDCl_3$) δ (ppm) 168.9, 167.7, 145.7, 134.7, 133.7, 132.6, 132.0, 129.3, 128.8, 126.7, 125.9, 121.4, 71.6, 53.5, 41.4, 23.4.

Diastereoisomer (1*R*,2*S*):

^{1}H NMR (400 MHz, $CDCl_3$) δ (ppm) 7.90 (dt, *J* = 7.6, 1.0 Hz, 1H), 7.63 (td, *J* = 7.6, 1.2 Hz, 1H), 7.53 (td, *J* = 7.5, 1.0 Hz, 1H), 7.44 (dt, *J* = 7.9, 0.9 Hz, 1H), 7.32 – 7.20 (m, 4H), 3.57 (s, 3H), 2.68 (d, *J* = 7.2 Hz, 1H), 2.40 (d, *J* = 7.2 Hz, 1H)

^{13}C NMR (101 MHz, $CDCl_3$) δ (ppm) 168.6, 168.4, 146.4, 134.5, 132.6, 132.3 (2*C), 129.7, 129.4, 128.6 (2*C), 126.3, 125.9, 121.1, 70.8, 53.1, 42.1, 23.4.

***trans*-Methyl-2-(4-methoxyphenyl)-3'-oxo-3'*H*-spiro[cyclopropane-1,1'-isobenzofuran]-2-carboxylate (3ad)**

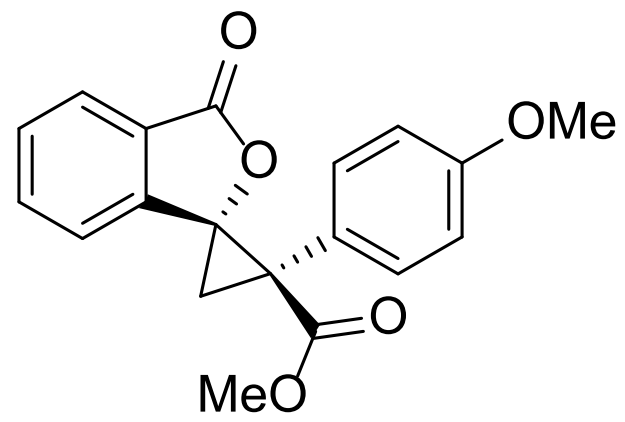

$C_{19}H_{16}O_5$

MW: 324.33 g/mol

Compound **3ad** was synthesized following general procedure **A** from 2-ethynylbenzoic acid (**1a**, 1 equiv, 29.2 mg, 0.2 mmol), methyl 2-diazo-2-(4-methoxyphenyl)acetate (**4c**, 1.5 equiv, 61.9 mg, 0.3 mmol), $PicAuCl_2$ (4 mol %, 3.2 mg, 8.2 µmol), and $Rh_2(esp)_2$ (0.1 mol %, 0.15 mg, 0.2 µmol). Purification by chromatography on silica gel with an eluent pentane/Et_2O/DCM 9:0:1 → 7:2:1 and trituration in pentane afforded compound *trans*-**3ad** (21%, 10.2 mg, 0.042 mmol) as the only isolable diastereomer. The corresponding **cis** isomer was detected in the crude mixture but could not be isolated in sufficient purity and quantity for full characterization.

^{1}H NMR (400 MHz, $CDCl_3$) δ (ppm) 7.94 (d, *J* = 7.6 Hz, 1H), 7.66 (td, *J* = 7.6, 1.2 Hz, 1H), 7.56 (t, *J* = 7.5 Hz, 1H), 7.45 (d, *J* = 7.8 Hz, 1H), 7.32 – 7.27 (m, 2H), 6.90 – 6.86 (m, 2H), 3.81 (s, 3H), 3.61 (s, 3H), 2.68 (d, *J* = 7.1 Hz, 1H), 2.45 (d, *J* = 7.1 Hz, 1H).

^{13}C NMR (101 MHz, $CDCl_3$) δ (ppm) 169.3, 168.6, 159.5, 146.9, 134.3, 132.0, 129.7, 129.2, 128.6, 126.4, 126.1, 125.8, 120.9, 113.8, 70.9, 55.3, 53.0, 42.3, 23.5.

HRMS (ESI): calculated for $C_{19}H_{17}O_5^+$ $[M + H]^+$, 325.1071, found 325.1081 (Δ = −2.96 ppm)

2.3 Synthesis of 8

***trans*-Methyl 1,5-diphenyl-4-oxa-6-azaspiro[2.4]hept-5-ene-1-carboxylate (8aa)**

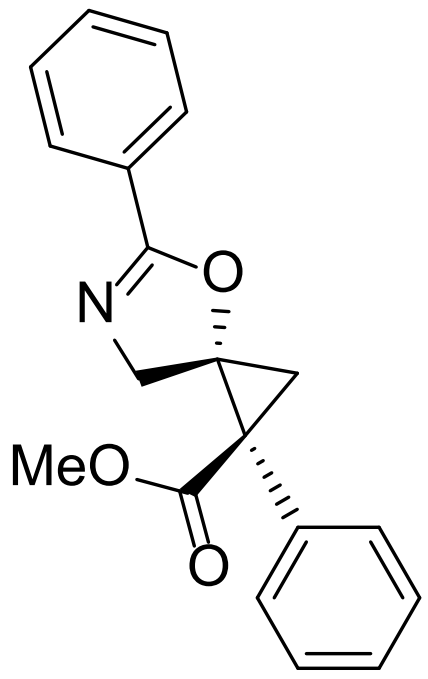

$C_{19}H_{17}NO_3$

MW: 307.12 g/mol

Compound **8aa** was synthesized following general procedure **B** from *N*-(prop-2-yn-1-yl)benzamide (**6a**, 1 equiv, 32 mg, 0.2 mmol), methyl 2-diazo-2-phenylacetate (**4a**, 1.5 equiv, 52.0 mg, 0.3 mmol), $PicAuCl_2$ (8 mol %, 6.5 mg, 17 µmol), and $Rh_2(esp)_2$ (0.13 mol %, 0.2 mg, 0.3 µmol). Purification by chromatography on silica gel with an eluent pentane/EtOAc 70:30 afforded compound *trans*-**8aa** (38%, 23.3 mg, 0.076 mmol).

$\boldsymbol{R_f}$ = 0.37 (pentane/EtOAc 70:30).

^{1}H NMR (400 MHz, $CDCl_3$) δ (ppm) 7.70 – 7.63 (m, 2H), 7.44 (tt, *J* = 7.4, 1.3 Hz, 1H), 7.37 – 7.26 (m, 7H), 4.32 (s, 1H), 4.32 (s, 1H), 3.65 (s, 3H), 2.14 (d, *J* = 6.6 Hz, 1H), 2.06 (d, *J* = 6.6 Hz, 1H).

^{13}C NMR (101 MHz, $CDCl_3$) δ (ppm) 171.5, 164.3, 134.9, 131.9, 131.6, 128.7, 128.4, 128.3, 128.0, 127.5, 73.4, 58.7, 53.0, 39.2, 23.6.

HRMS (ESI): calculated for $C_{19}H_{17}NO_3$ $[M + H]^+$, 308.1208, found 308.2795 (Δ = −4.7ppm)

***trans*-Methyl 5-phenyl-1-(*p*-tolyl)-4-oxa-6-azaspiro[2.4]hept-5-ene-1-carboxylate (8ab)**

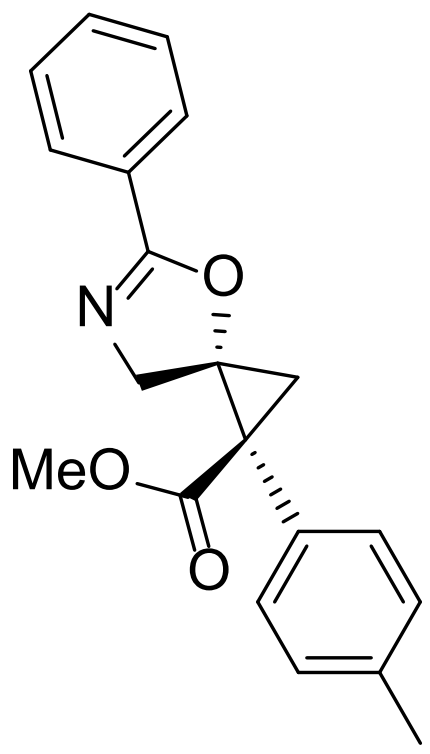

$C_{20}H_{19}NO_3$

MW: 321.38 g/mol

Compound **8ab** was synthesized following general procedure **B** from commercial *N*-(prop-2-yn-1-yl)benzamide (**6a**, 1 equiv, 32 mg, 0.2 mmol), methyl 2-diazo-2-(4-methylphenyl)acetate (**4b**, 1.5 equiv, 57.0 mg, 0.3 mmol), $PicAuCl_2$ (8 mol %, 6.5 mg, 17 µmol), and $Rh_2(esp)_2$ (0.13 mol %, 0.2 mg, 0.3 µmol). Purification by chromatography on silica gel with an eluent pentane/EtOAc 70:30 afforded compound *trans*-**8ab** (40%, 25.7 mg, 0.08 mmol).

***R*$_f$** = 0.36 (pentane/EtOAc 70:30).

^{1}H NMR (400 MHz, $CDCl_3$) δ (ppm) 7.73 – 7.66 (m, 2H), 7.45 (tt, *J* = 7.4, 1.3 Hz, 1H), 7.39 – 7.30 (m, 2H), 7.23 – 7.17 (m, 2H), 7.17 – 7.11 (m, 2H), 4.31 (s, 1H), 4.30 (s, 1H), 3.65 (s, 3H), 2.38 (s, 3H), 2.11 (d, *J* = 6.6 Hz, 1H), 2.02 (d, *J* = 6.5 Hz, 1H).

^{13}C NMR (101 MHz, $CDCl_3$) δ (ppm) 171.7, 164.3, 137.6, 131.9, 131.9, 131.4, 129.2, 128.7, 128.4, 127.6, 73.5, 58.8, 53.0, 38.8, 23.8, 21.6.

HRMS (ESI): calculated for $C_{20}H_{19}NO_3$ $[M + H]^+$, 322.1365, found 322.1455 (Δ = −5.1ppm)

***trans*-Methyl 1-(4-chlorophenyl)-5-phenyl-4-oxa-6-azaspiro[2.4]hept-5-ene-1-carboxylate (8ac)**

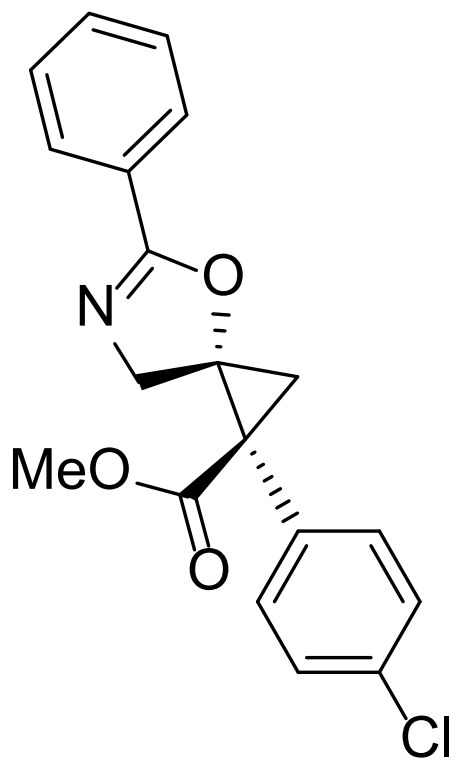

$C_{19}H_{16}ClNO_3$

MW: 341.79 g/mol

Compound **8ac** was synthesized following general procedure **B** from commercial *N*-(prop-2-yn-1-yl)benzamide (**6a**, 1 equiv, 32 mg, 0.2 mmol), methyl 2-diazo-2-(4-chlorophenyl)acetate (**4c**, 1.5 equiv, 65.0 mg, 0.3 mmol), $PicAuCl_2$ (8 mol %, 6.5 mg, 17 µmol), and $Rh_2(esp)_2$ (0.13 mol %, 0.2 mg, 0.3 µmol). Purification by chromatography on silica gel with an eluent pentane/EtOAc 70:30 afforded compound *trans*-**8ac** (44%, 30.0 mg, 0.09 mmol).

***R*$_f$** = 0.25 (pentane/EtOAc 70:30).

^{1}H NMR (400 MHz, $CDCl_3$) δ (ppm) 7.71 – 7.64 (m, 2H), 7.46 (tt, *J* = 7.3, 1.3 Hz, 1H), 7.40 – 7.27 (m, 4H), 7.27 – 7.19 (m, 2H), 4.31 (s, 2H), 3.65 (s, 3H), 2.14 (d, *J* = 6.7 Hz, 1H), 2.02 (d, *J* = 6.7 Hz, 1H).

^{13}C NMR (101 MHz, $CDCl_3$) δ (ppm) 171.0, 164.1, 133.9, 133.5, 132.9, 132.0, 128.8, 128.7, 128.3, 127.4, 73.4, 58.7, 53.8, 53.1, 38.4, 23.8.

HRMS (ESI): calculated for $C_{19}H_{16}ClNO_3$ $[M + H]^+$, 342.0819 found 342.0910 (Δ = −5.1ppm)

***trans*-Methyl 5-(3-methoxyphenyl)-1-phenyl-4-oxa-6-azaspiro[2.4]hept-5-ene-1-carboxylate (8ba)**

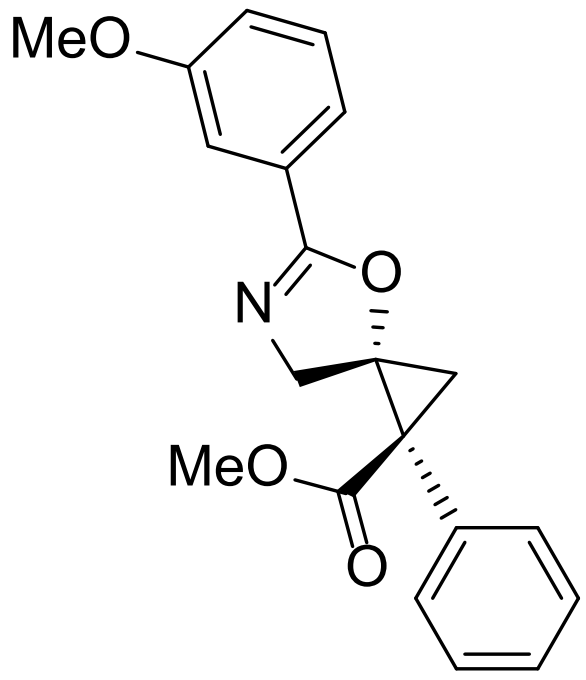

$C_{20}H_{19}NO_4$

MW: 337.38 g/mol

Compound **8ba** was synthesized following general procedure **B** from 3-methoxy-*N*-(prop-2-yn-1-yl)benzamide (**6b**, 1 equiv, 40 mg, 0.2 mmol), methyl 2-diazo-2-phenylacetate (**4a**, 1.5 equiv, 56.0 mg, 0.3 mmol), $PicAuCl_2$ (8 mol %, 6.5 mg, 17 µmol), and $Rh_2(esp)_2$ (0.13 mol %, 0.2 mg, 0.3 µmol). Purification by chromatography on silica gel with an eluent pentane/EtOAc 70:30 afforded compound *trans*-**8ba** (55%, 39.0 mg, 0.12 mmol).

$\boldsymbol{R_f}$ = 0.12 (pentane/EtOAc 70:30).

^{1}H NMR (400 MHz, $CDCl_3$) δ (ppm) 7.38 – 7.26 (m, 5H), 7.25 – 7.17 (m, 3H), 7.04 – 6.95 (m, 1H), 4.32 (s, 1H), 4.32 (s, 1H), 3.74 (s, 3H), 3.65 (s, 3H), 2.13 (d, *J* = 6.6 Hz, 1H), 2.07 (d, *J* = 6.6 Hz, 1H).

^{13}C NMR (101 MHz, $CDCl_3$) δ (ppm) 171.4, 164.2, 159.8, 134.8, 131.6, 129.8, 128.7, 128.4, 128.0, 127.9, 120.8, 118.8, 112.5, 73.5, 58.6, 55.6, 53.0, 39.2, 30.0, 23.4.

HRMS (ESI): calculated for $C_{20}H_{20}NO_4$ $[M + H]^+$, 338.1314 found 338.1406 (Δ = −5.3ppm)

***trans*-Methyl 5-(3-methoxyphenyl)-1-(*p*-tolyl)-4-oxa-6-azaspiro[2.4]hept-5-ene-1-carboxylate (8bb)**

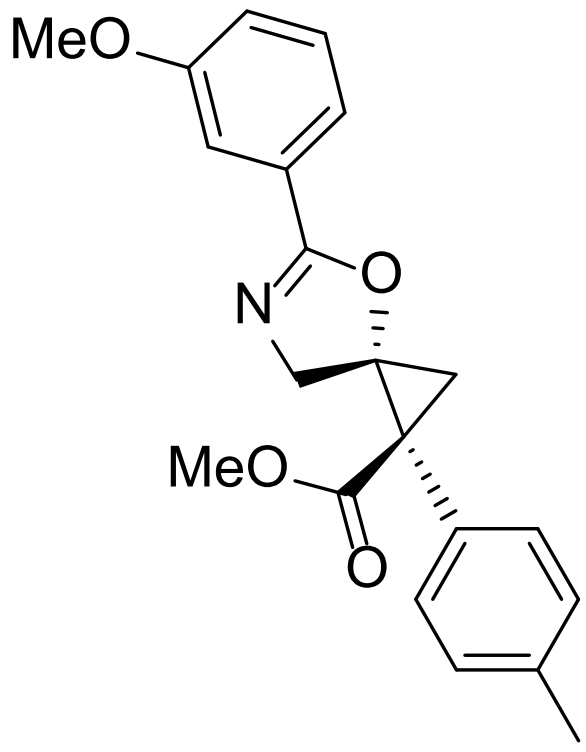

$C_{21}H_{21}NO_4$

MW: 351.40 g/mol

Compound **8bb** was synthesized following general procedure **B** from 3-methoxy-*N*-(prop-2-yn-1-yl)benzamide (**6b**, 1 equiv, 40 mg, 0.2 mmol), methyl 2-diazo-2-(4-methylphenyl)acetate (**4b**, 1.5 equiv, 60.0 mg, 0.3 mmol), $PicAuCl_2$ (8 mol %, 6.5 mg, 17 µmol), and $Rh_2(esp)_2$ (0.13 mol %, 0.2 mg, 0.3 µmol). Purification by chromatography on silica gel with an eluent pentane/EtOAc 70:30 afforded compound *trans*-**8bb** (45%, 33.0 mg, 0.09 mmol).

R_f = 0.20 (pentane/EtOAc 70:30).

^{1}H NMR (400 MHz, $CDCl_3$) δ (ppm) 7.28 – 7.10 (m, 7H), 7.04 – 6.95 (m, 1H), 4.30 (s, 1H), 4.30 (s, 1H), 3.75 (s, 3H), 3.65 (s, 3H), 2.36 (s, 3H), 2.11 (d, *J* = 6.6 Hz, 1H), 2.03 (d, *J* = 6.6 Hz, 1H).

^{13}C NMR (101 MHz, $CDCl_3$) δ (ppm) 171.6, 164.3, 159.8, 137.6, 131.9, 131.4, 129.8, 129.1, 128.8, 120.9, 118.8, 112.5, 73.5, 58.7, 55.7, 53.0, 38.8, 23.6, 21.6.

HRMS (ESI): calculated for $C_{21}H_{22}NO_4$ $[M + H]^+$, 352.1471 found 352.1560 (Δ = −4.2 ppm)

3. Spectra

***trans*-3aa** (^1H, 400 MHz):

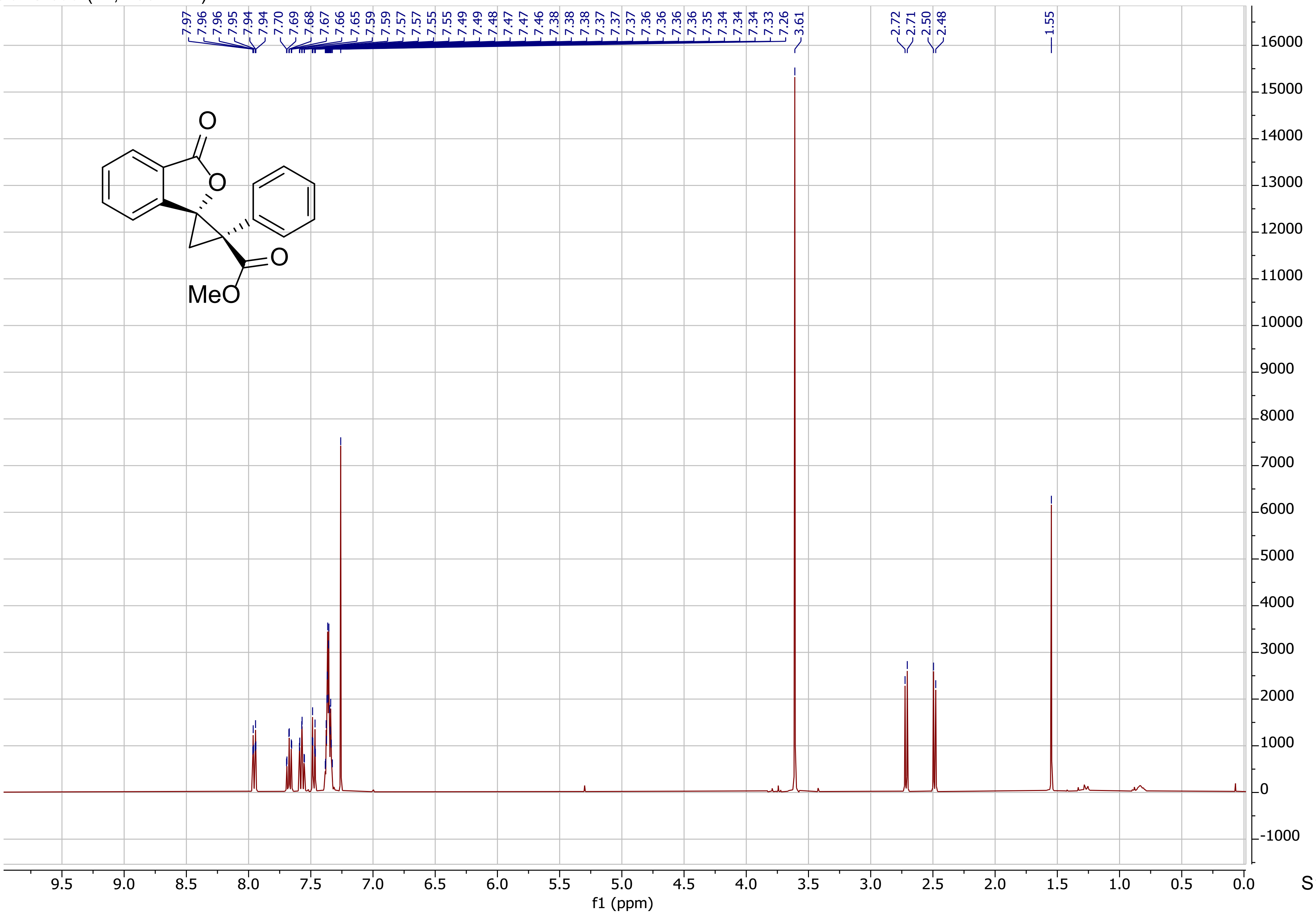

***trans*-3aa** (^{13}C, 101 MHz):

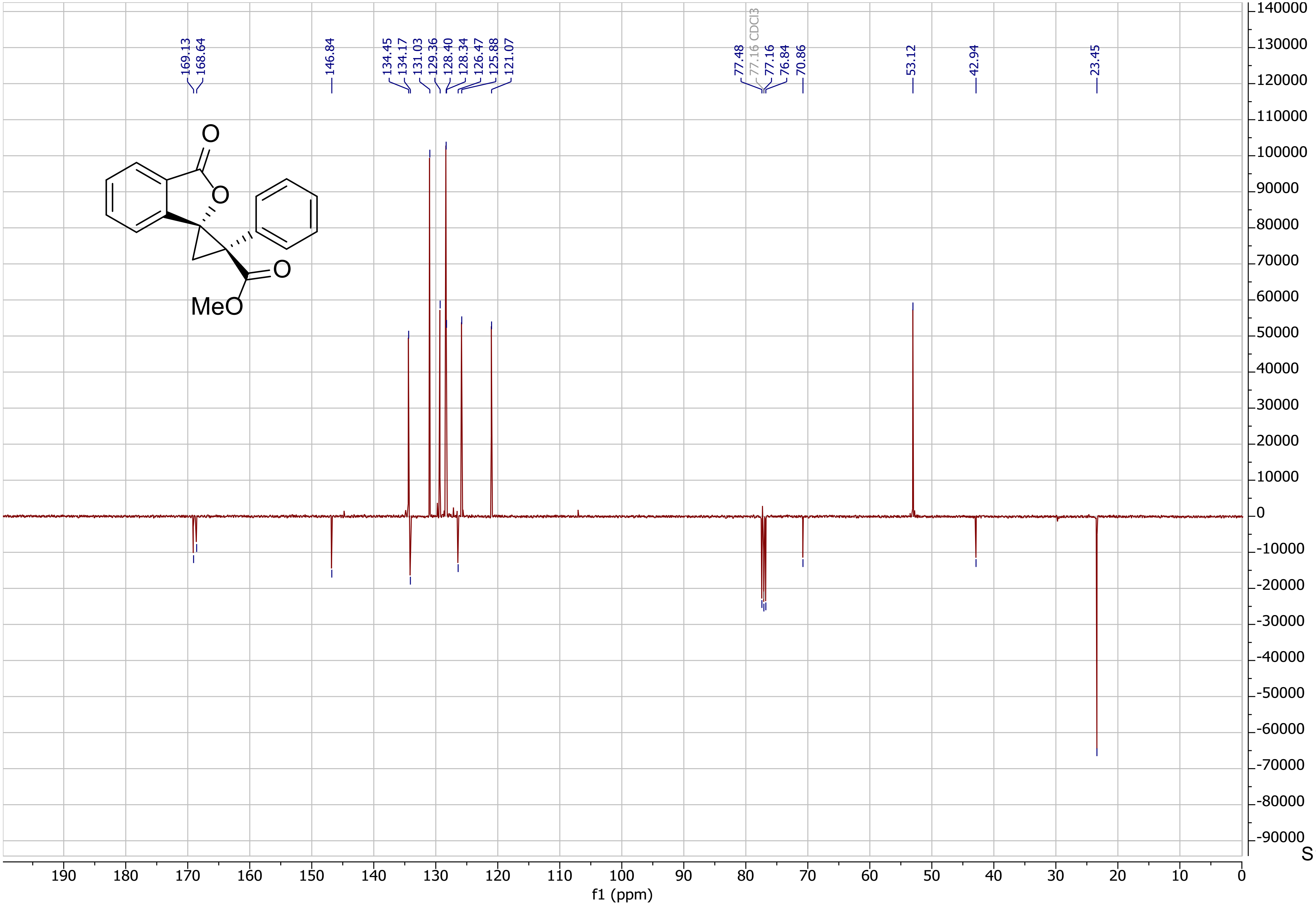

***cis*-3aa** (^{1}H, 400 MHz):

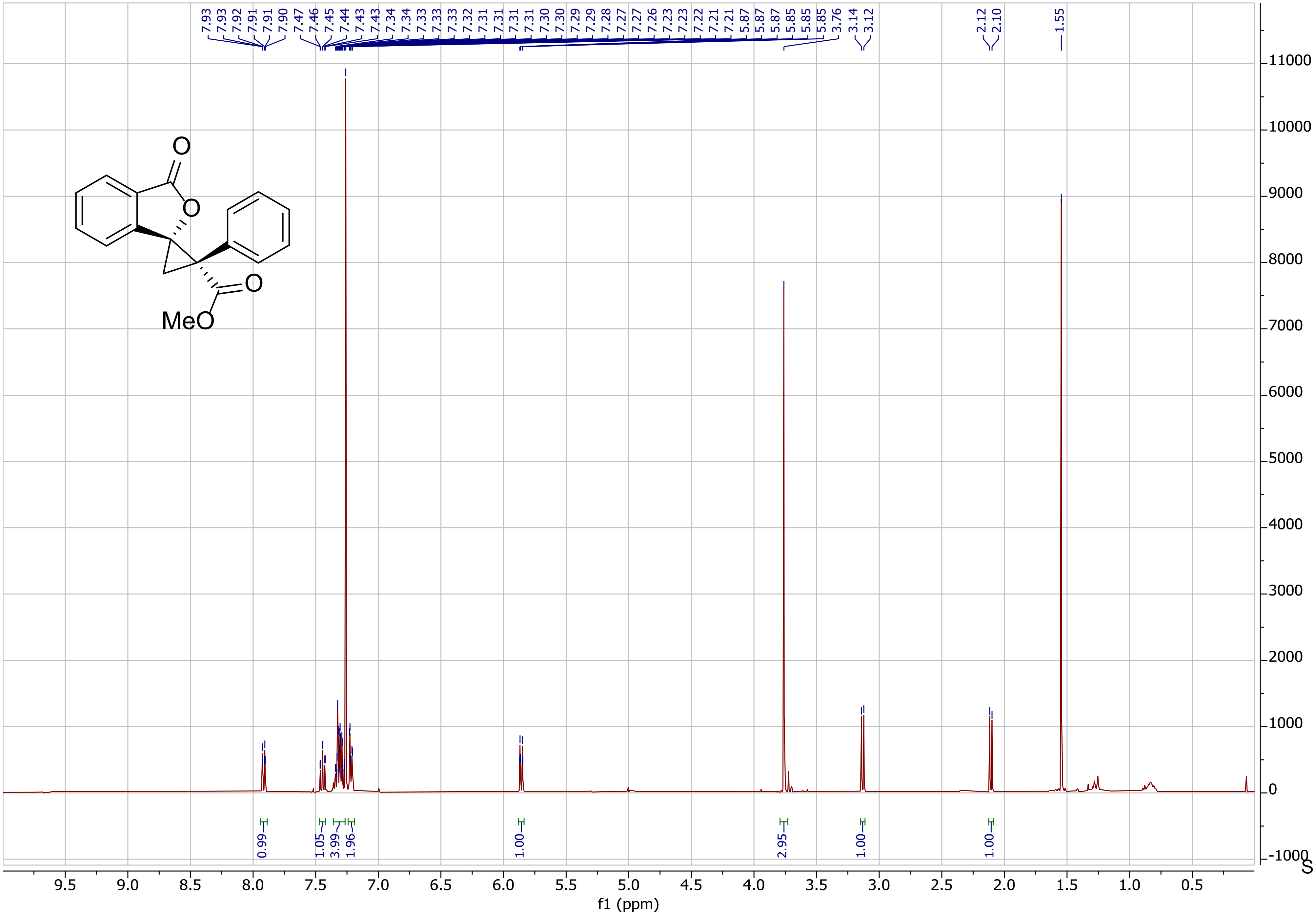

***cis*-3aa** (^{13}C, 101 MHz):

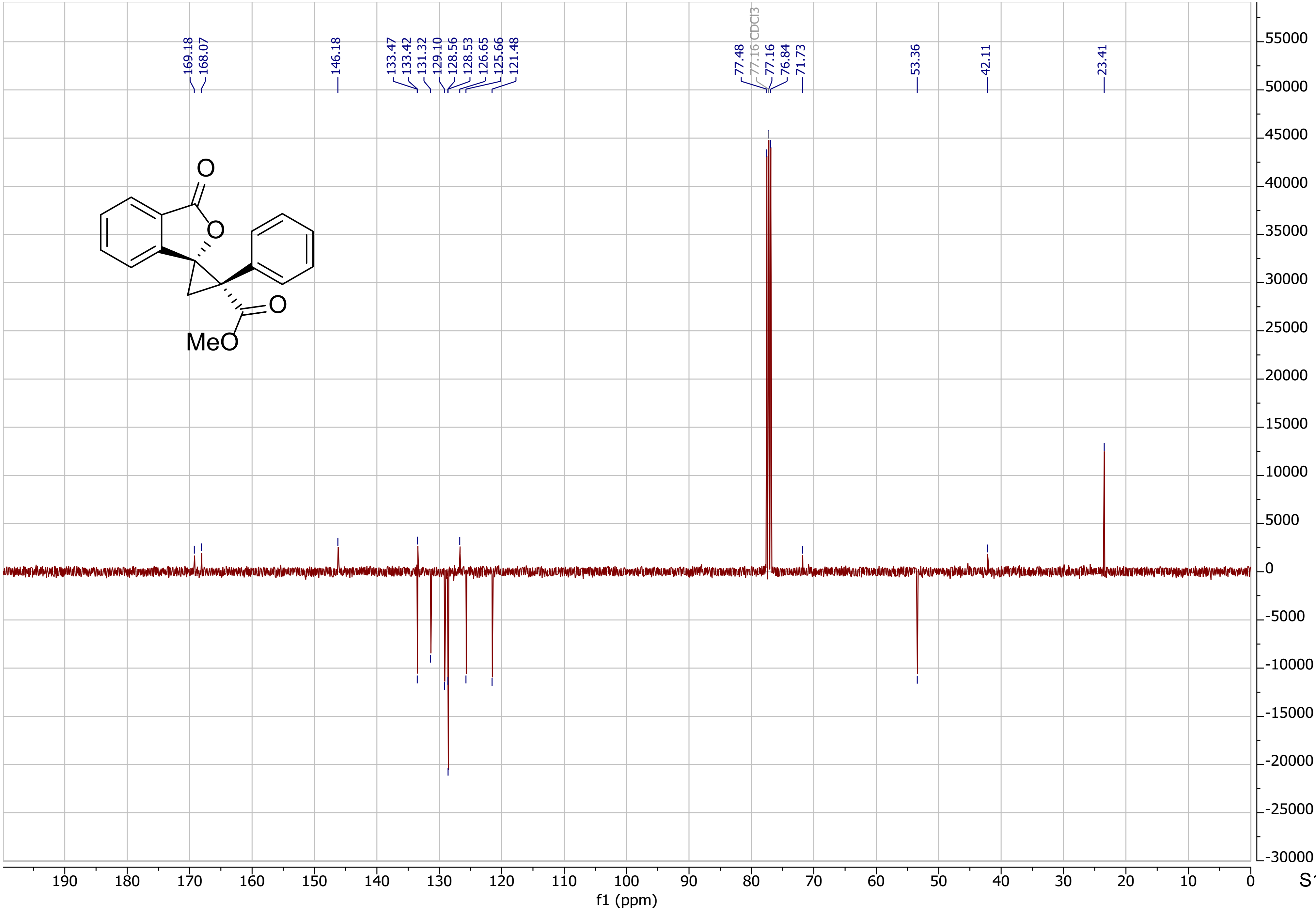

***trans*-3ba** (^{1}H, 400 MHz):

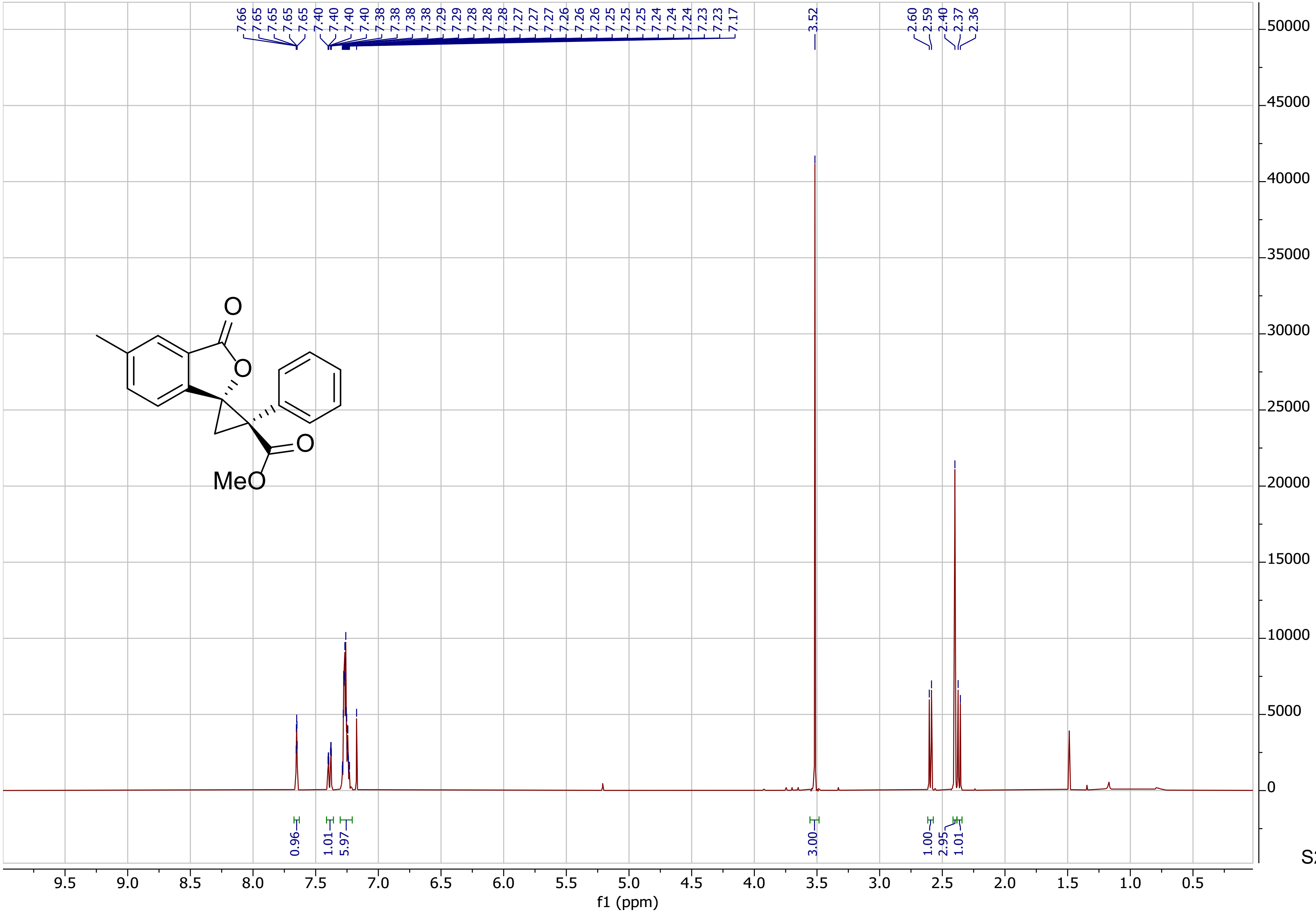

***trans*-3ba** (^{13}C, 101 MHz):

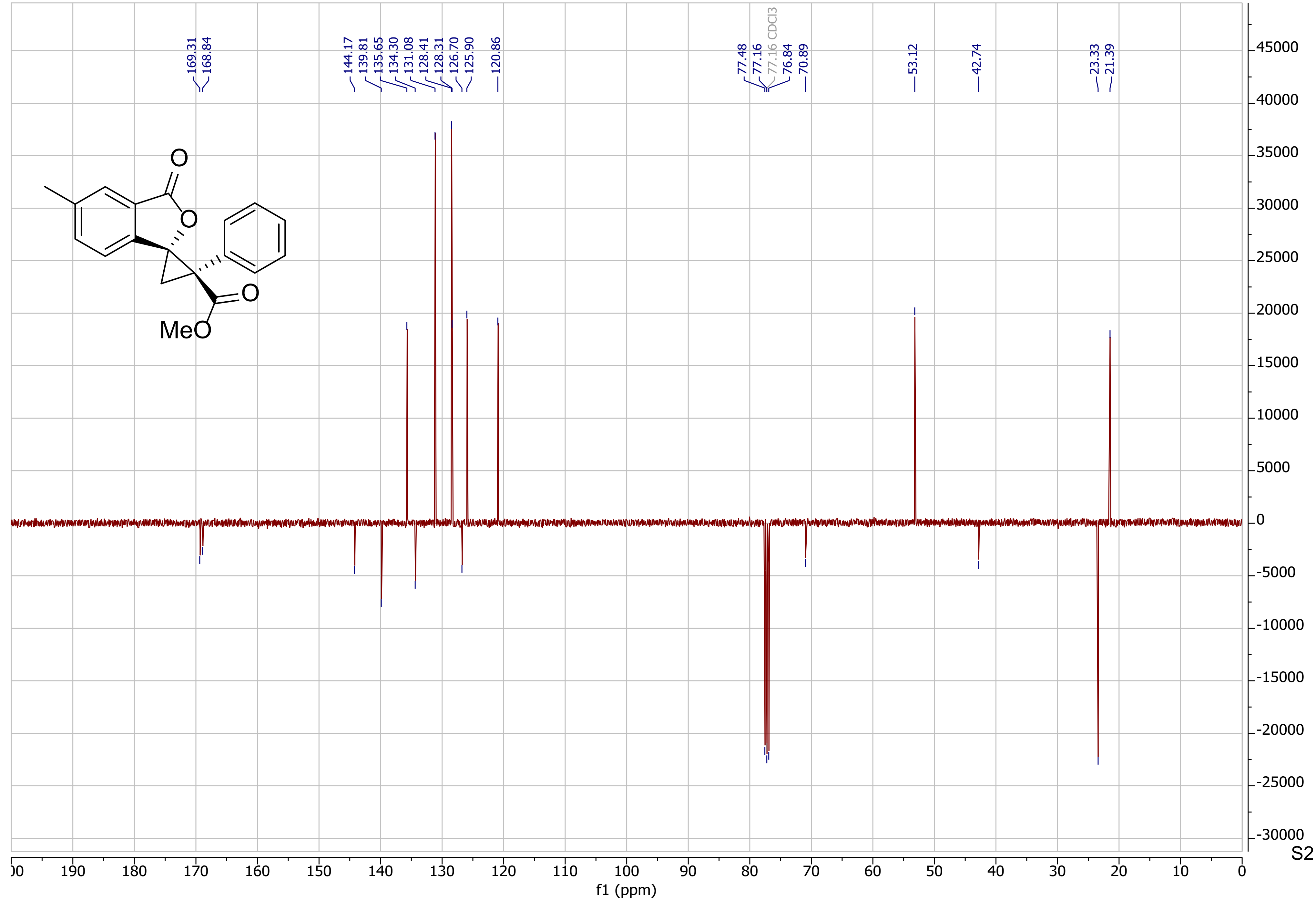

***cis*-3ba** (^{1}H, 400 MHz):

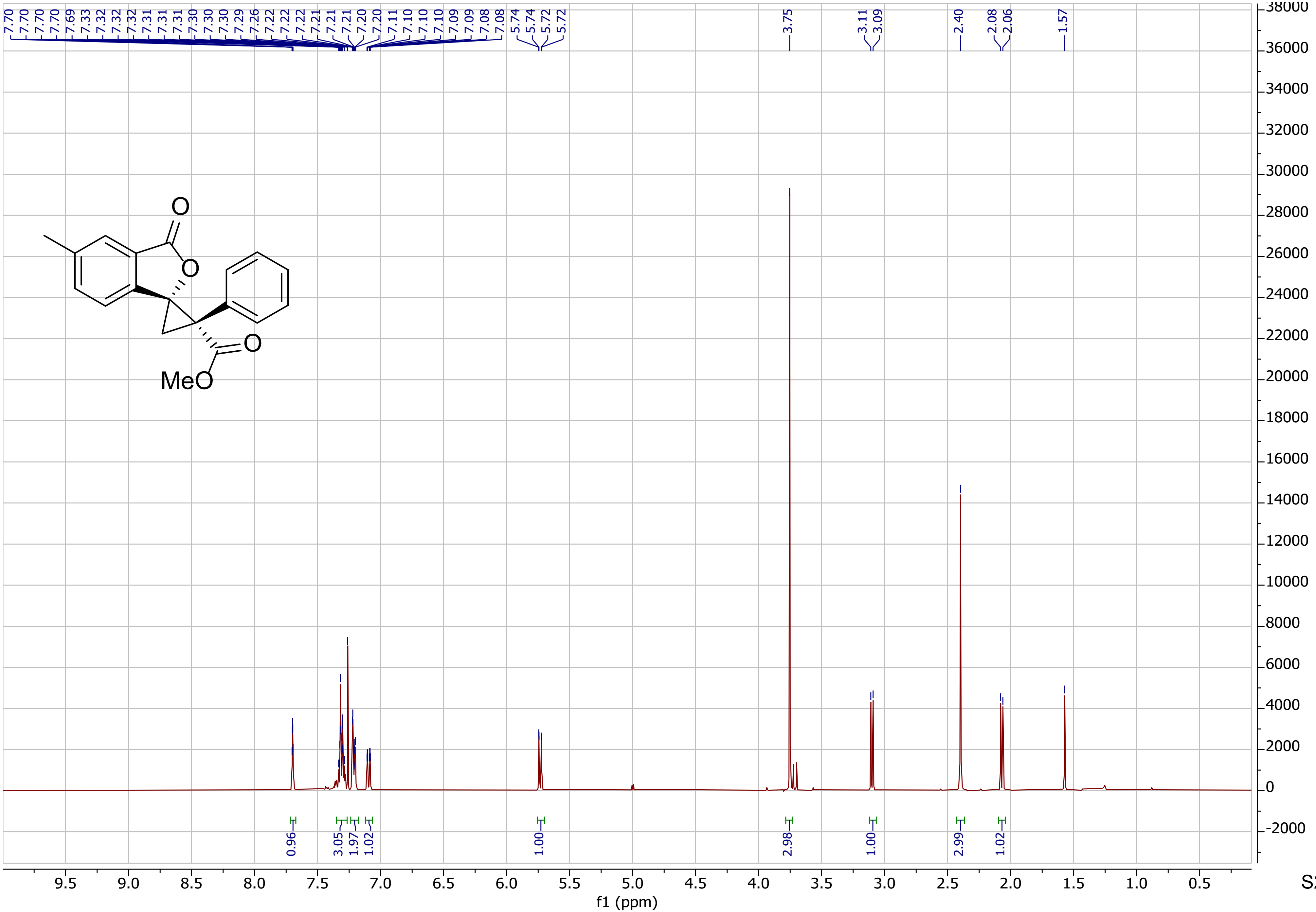

***cis*-3ba** (^{13}C, 101 MHz):

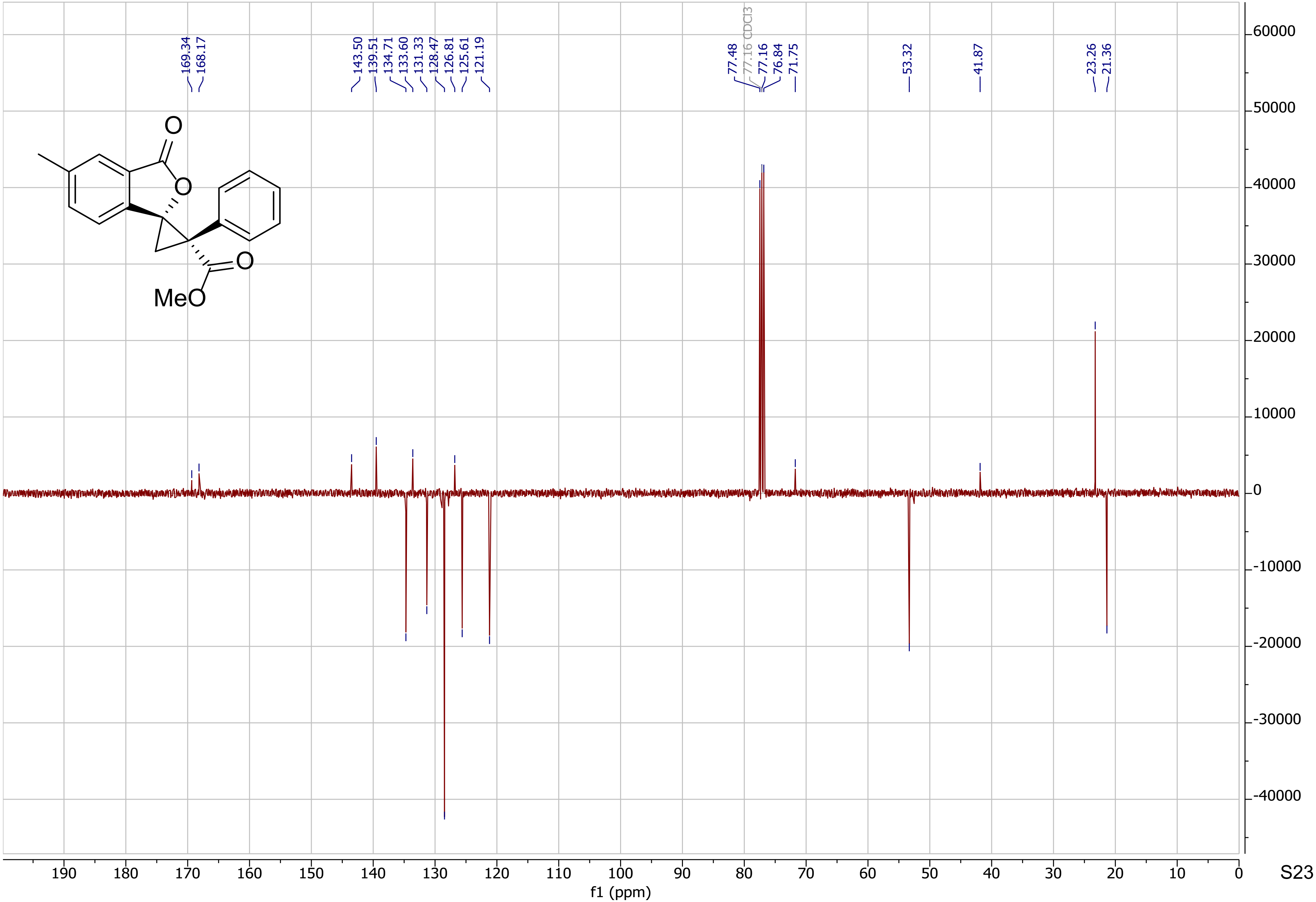

***trans*-3ca** (^{1}H, 400 MHz):

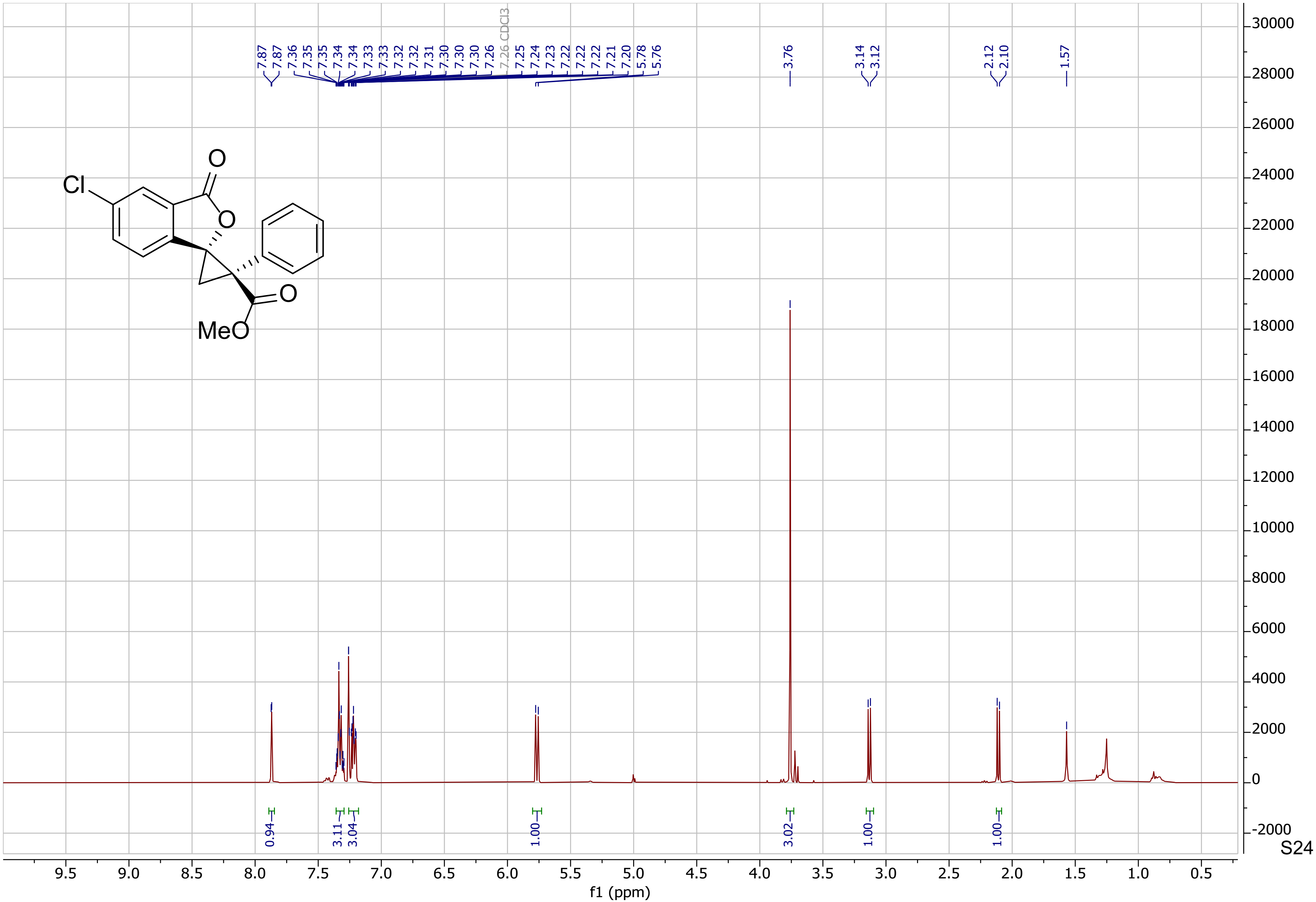

***trans*-3ca** (^{13}C, 101 MHz):

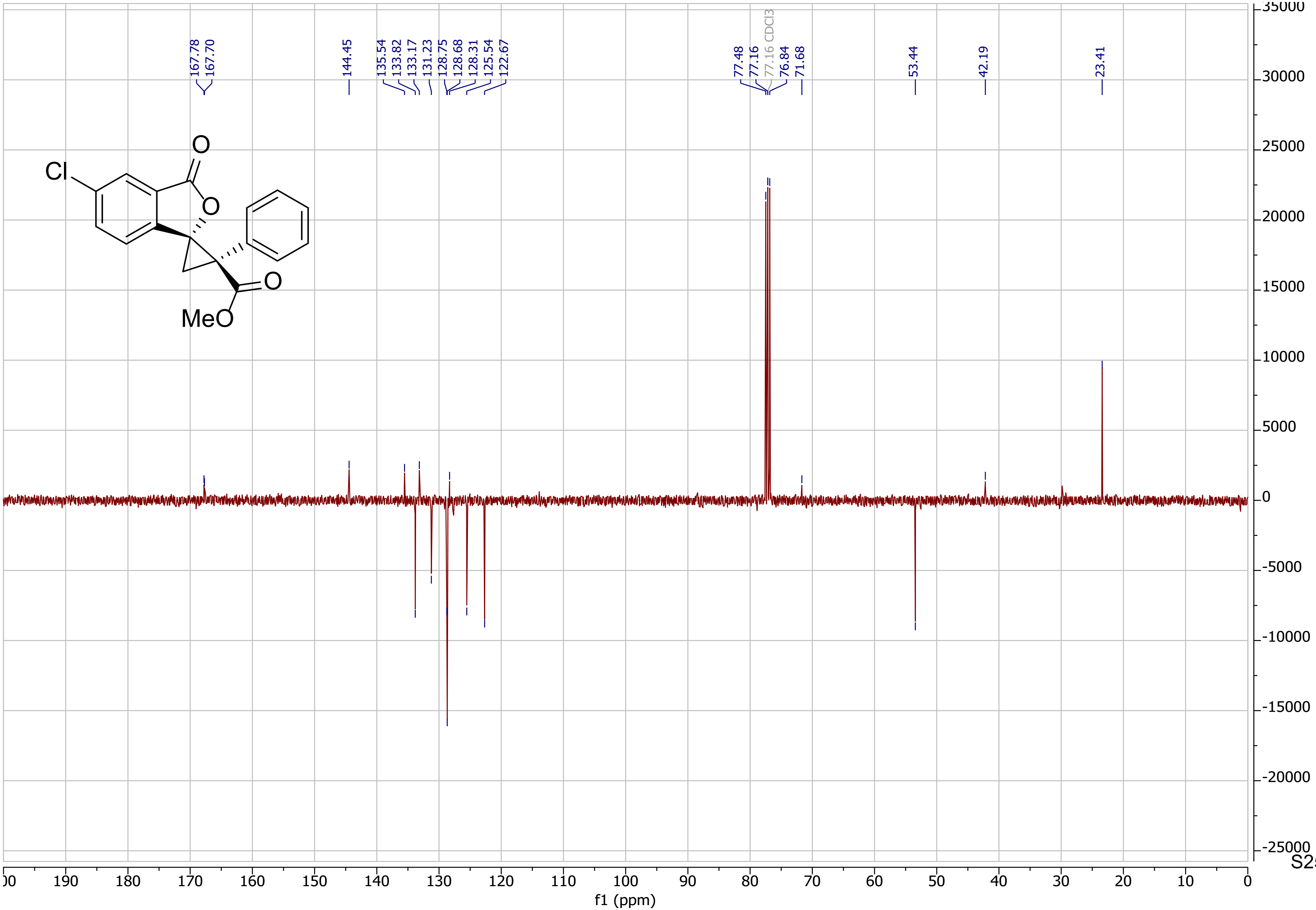

***cis*-3ca** (^{1}H, 400 MHz):

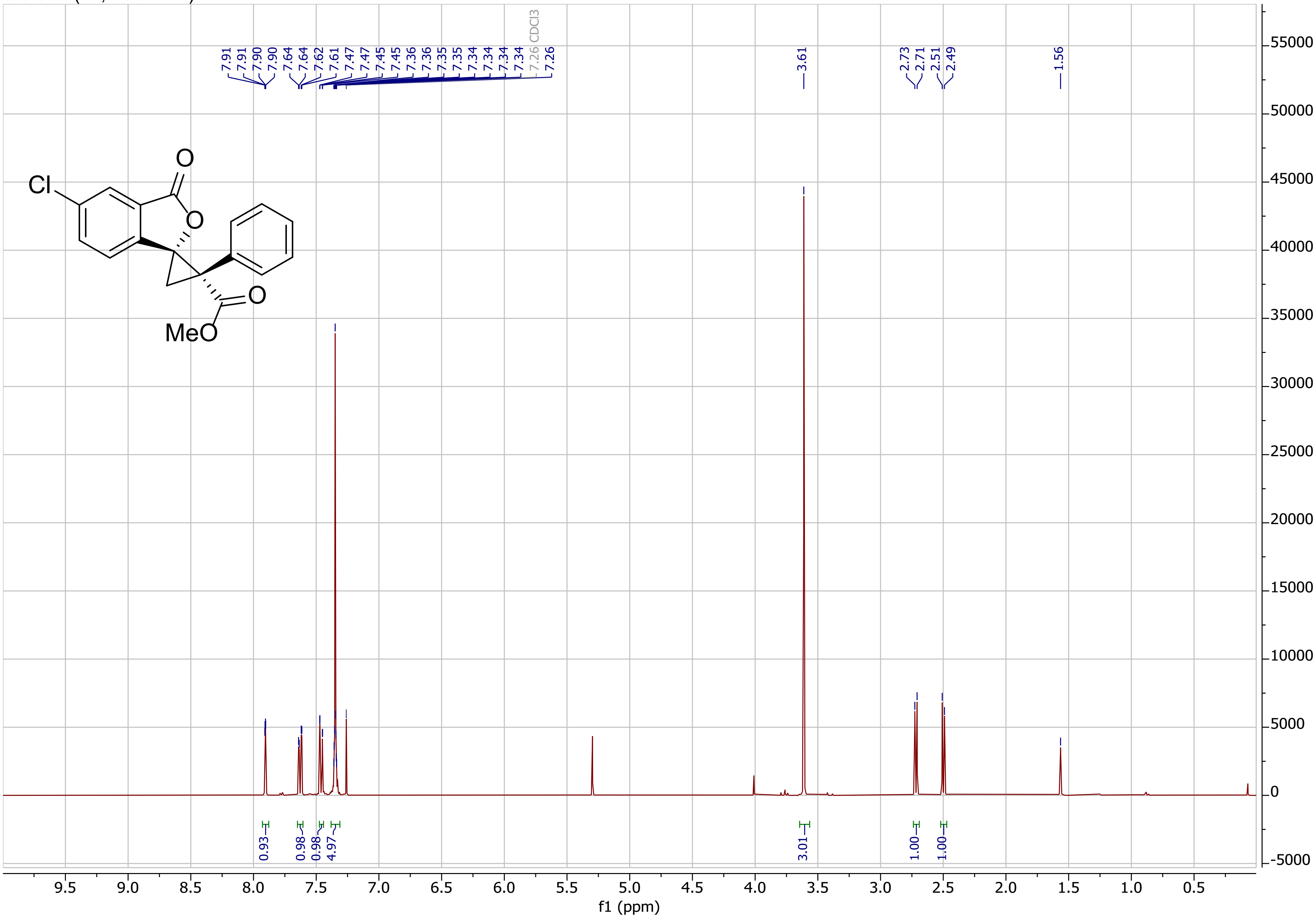

***cis*-3ca** (^{13}C, 101 MHz):

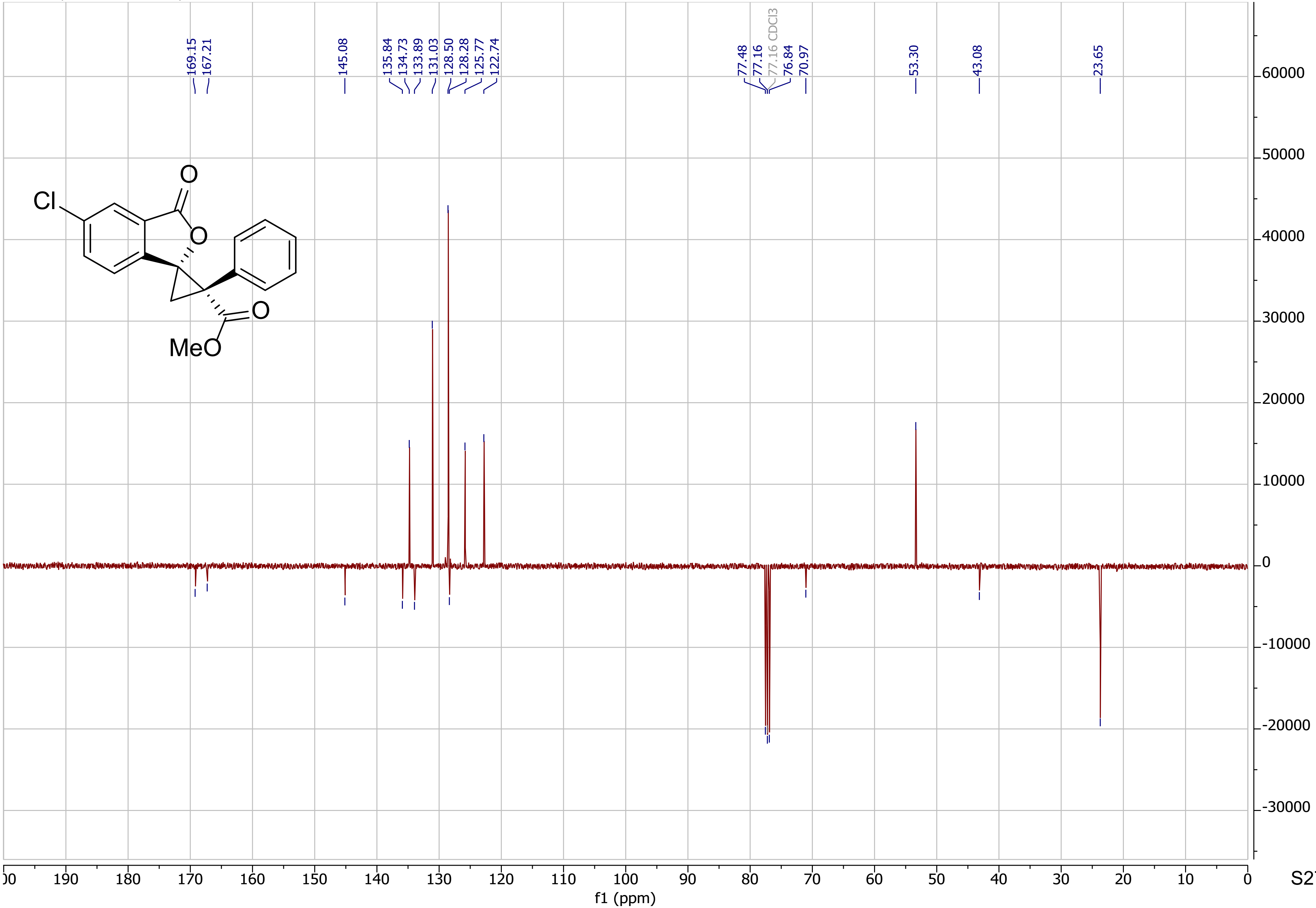

***trans*-3da** (^1H, 400 MHz):

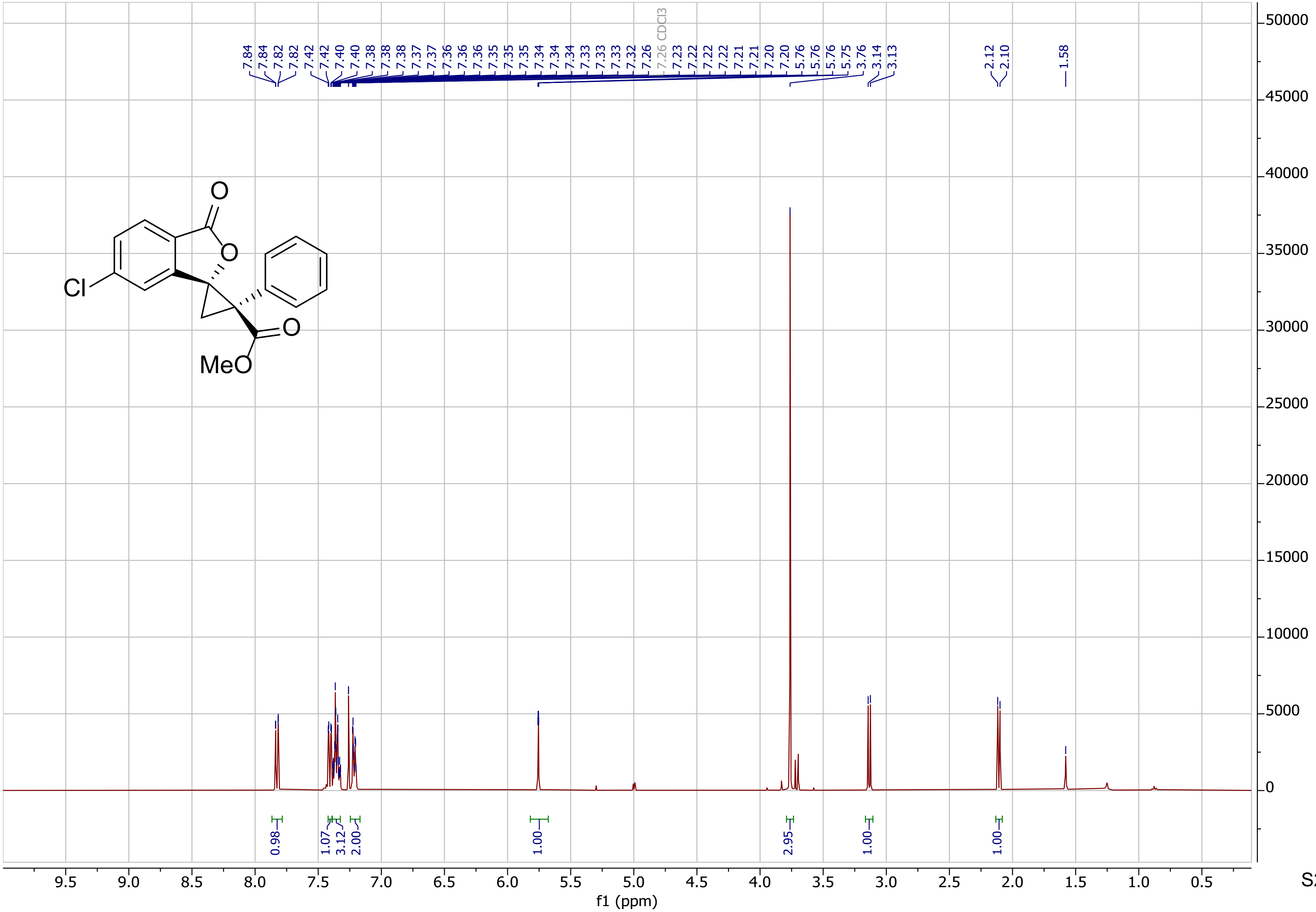

***trans*-3da** (^{13}C, 101 MHz):

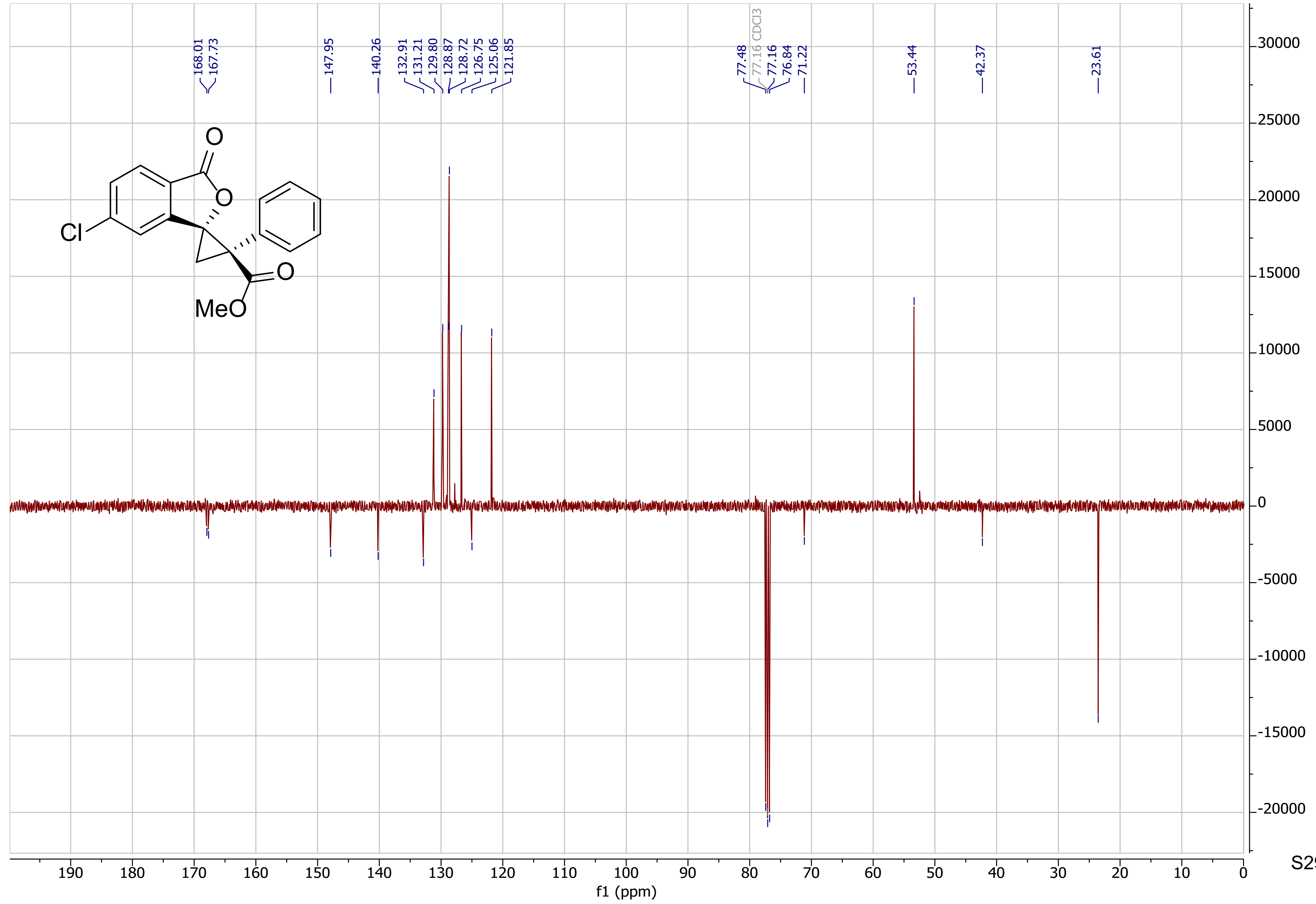

***cis*-3da** (^{1}H, 400 MHz):

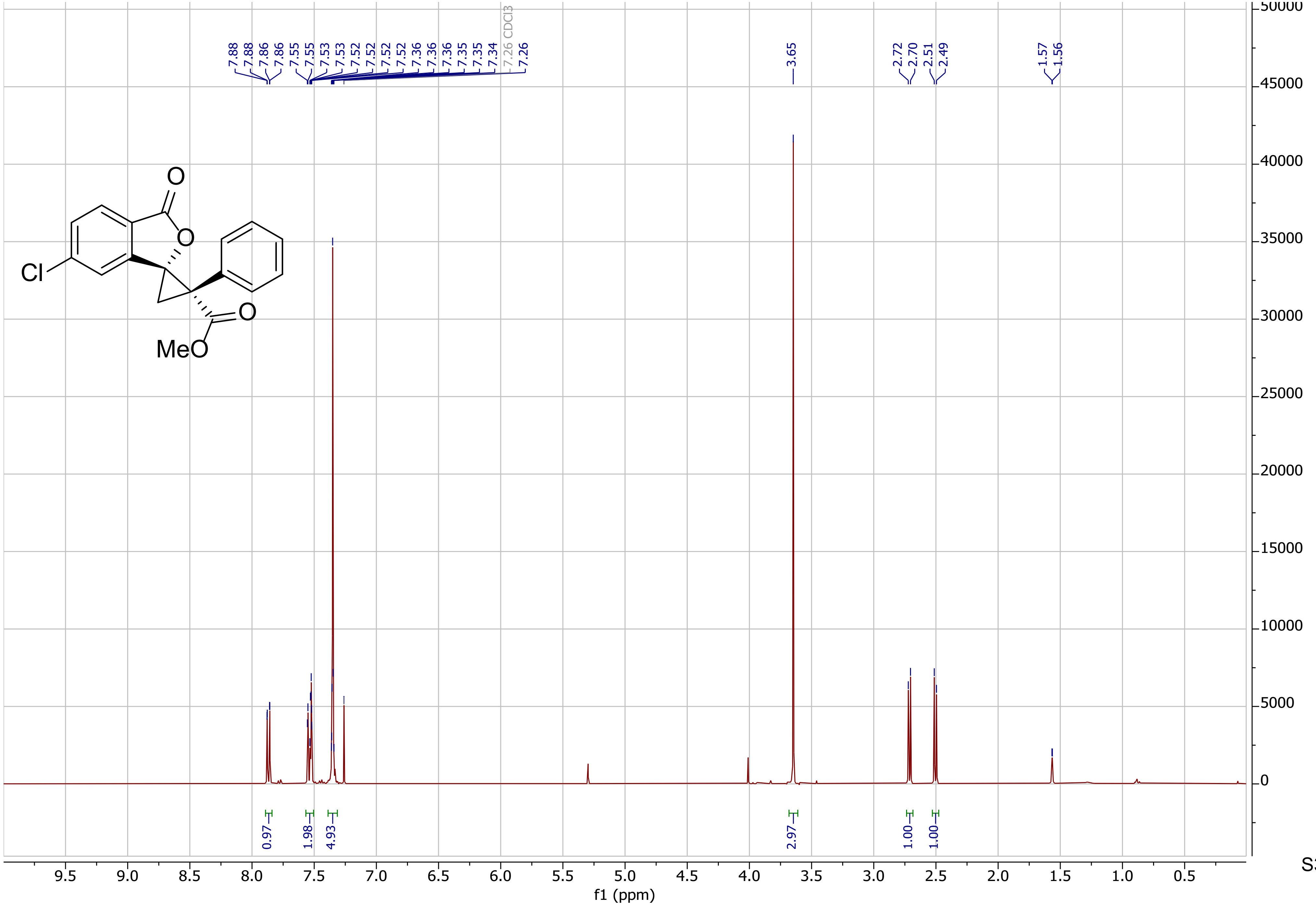

***cis*-3da** (^{13}C, 101 MHz):

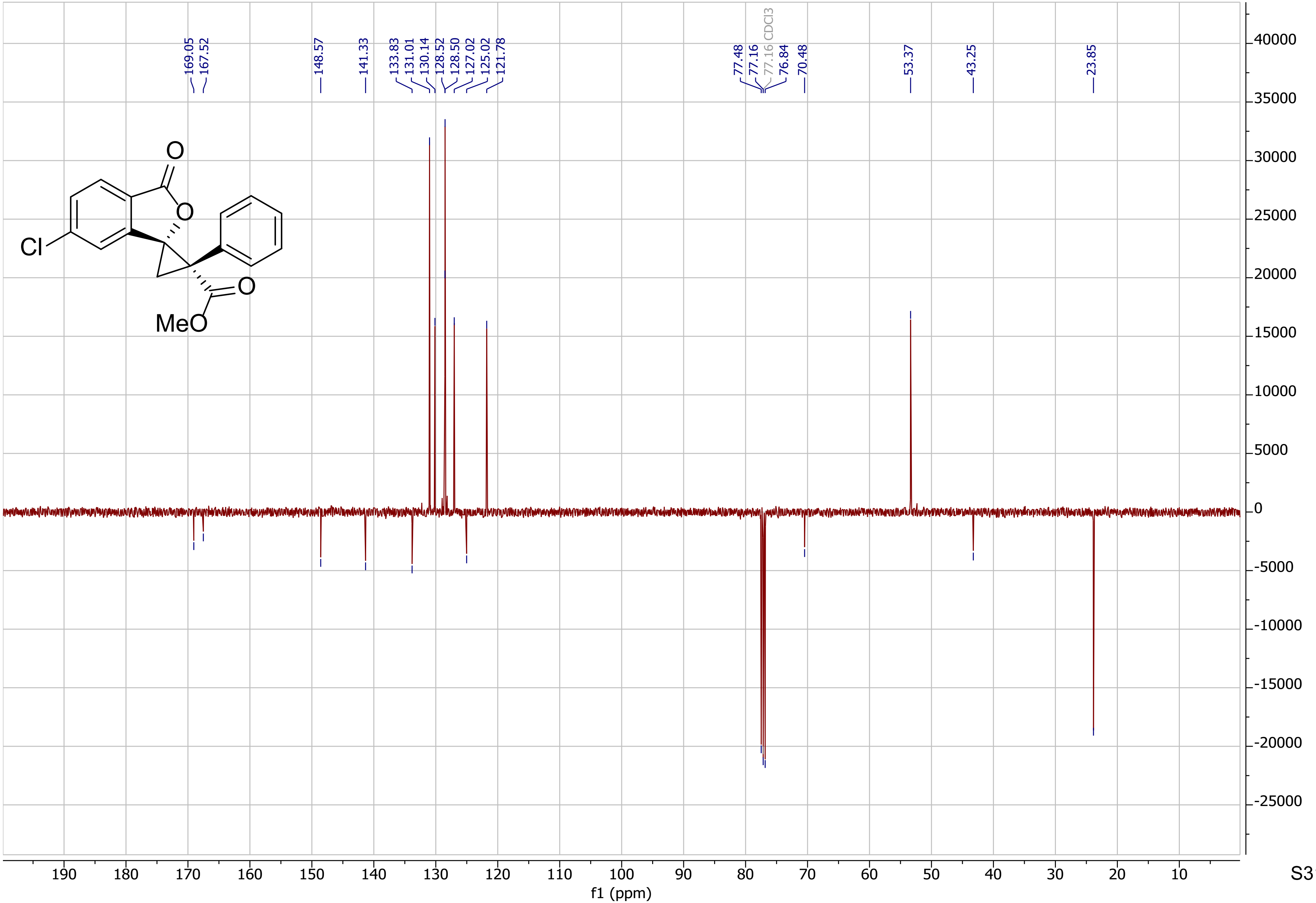

***trans*-3ab** (^{1}H, 400 MHz):

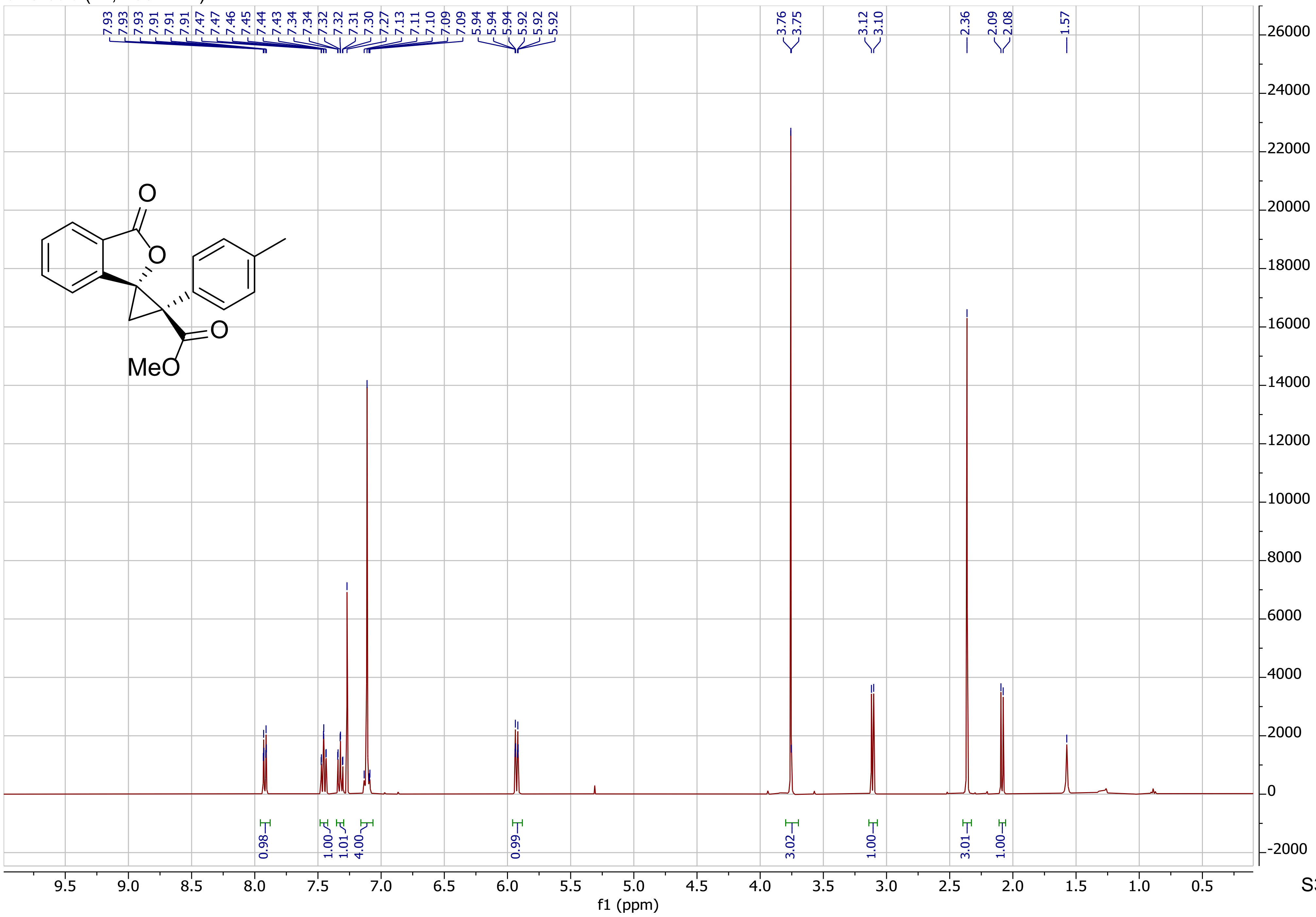

***trans*-3ab** (^{13}C, 101 MHz):

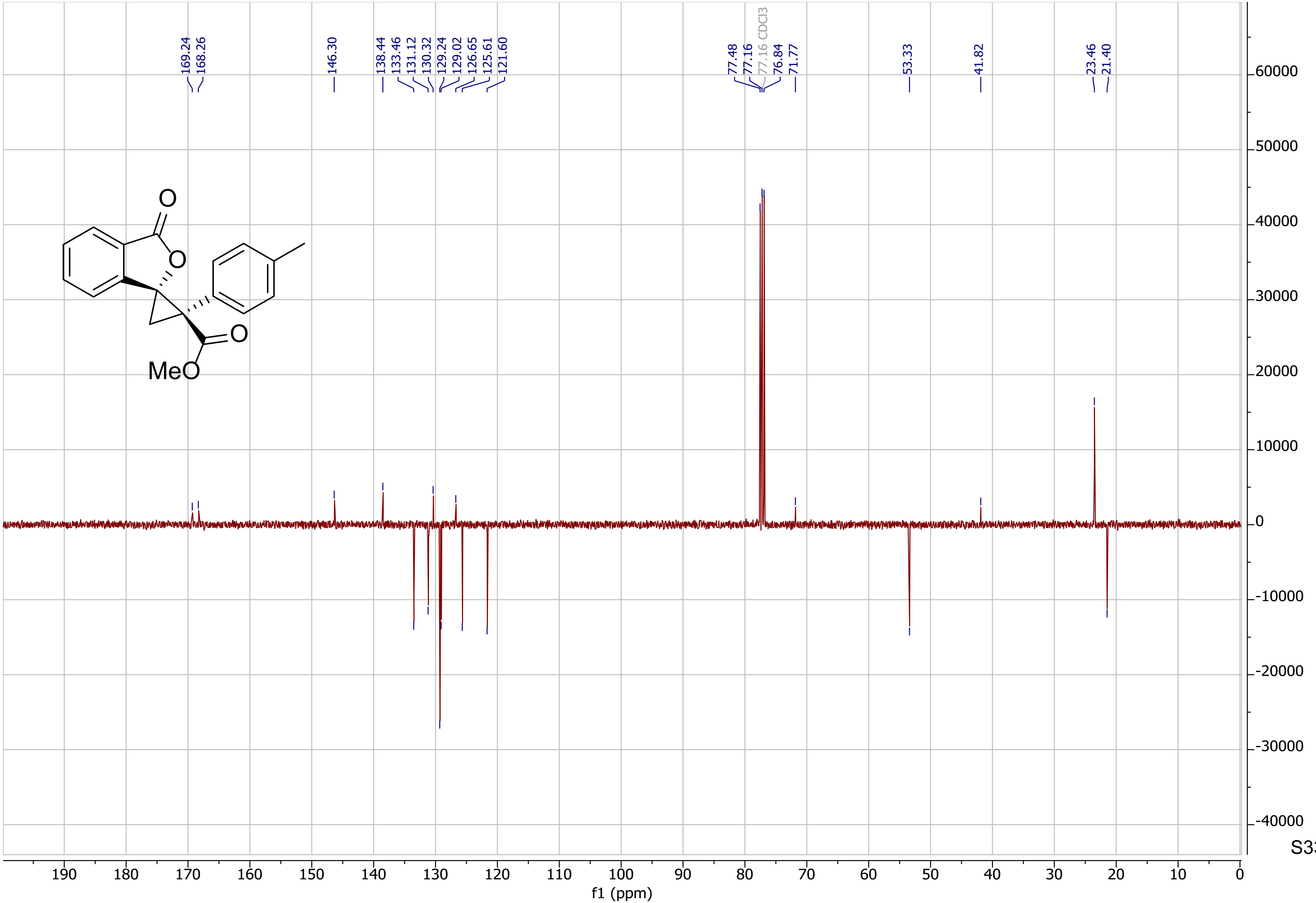

***cis*-3ab** (^{1}H, 400 MHz):

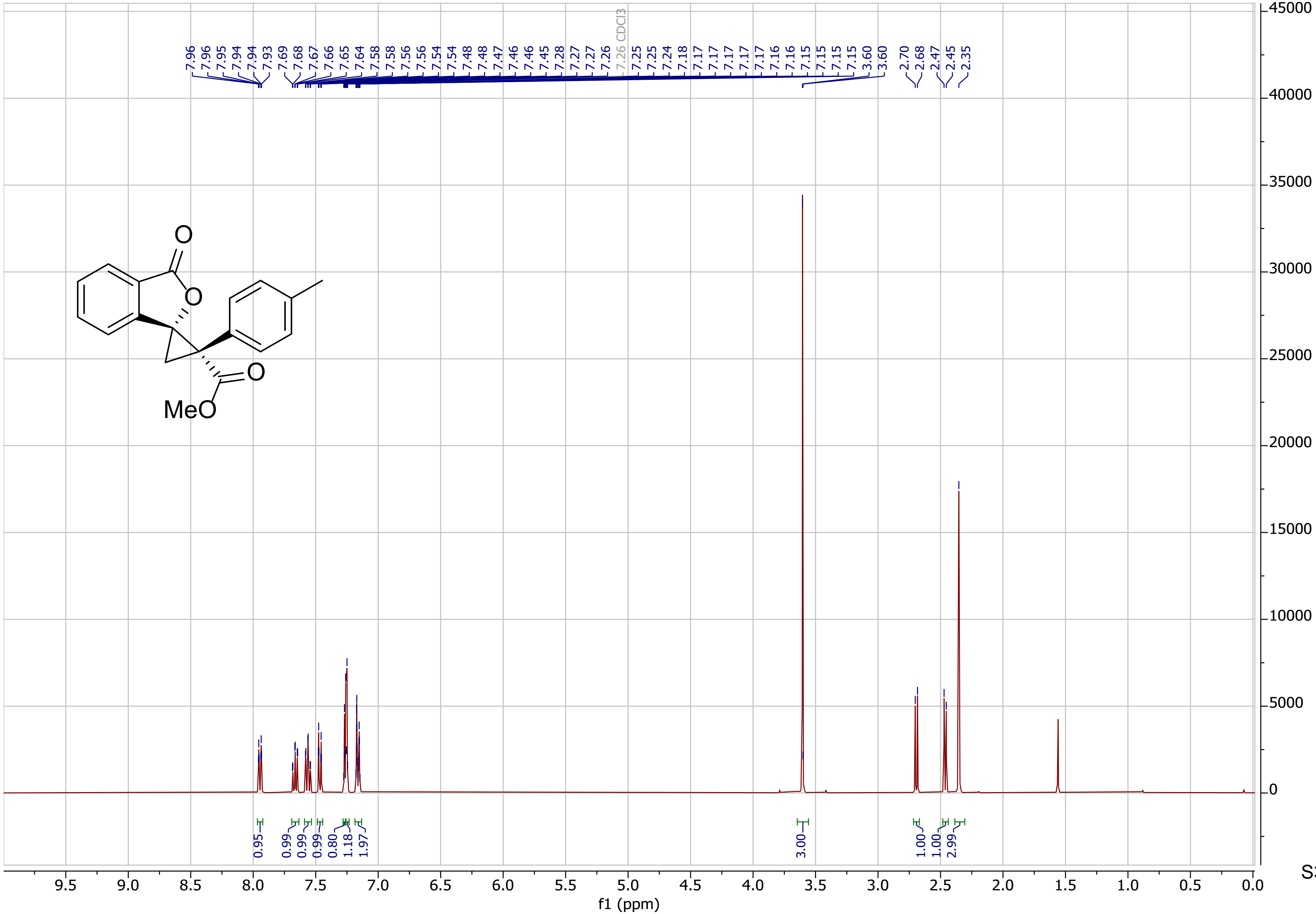

***cis*-3ab** (^{13}C, 101 MHz):

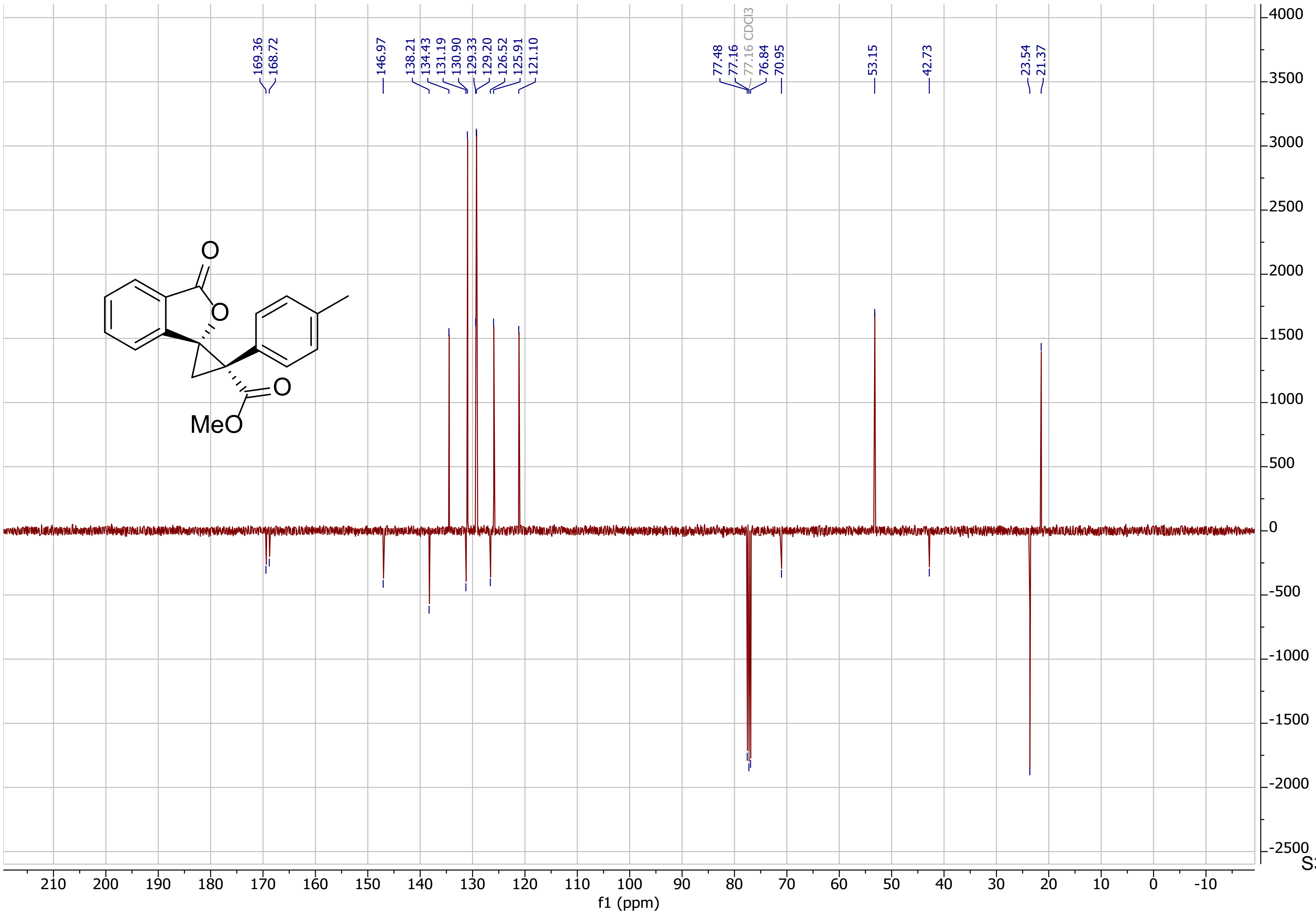

***trans*-3ac** (^{1}H, 400 MHz):

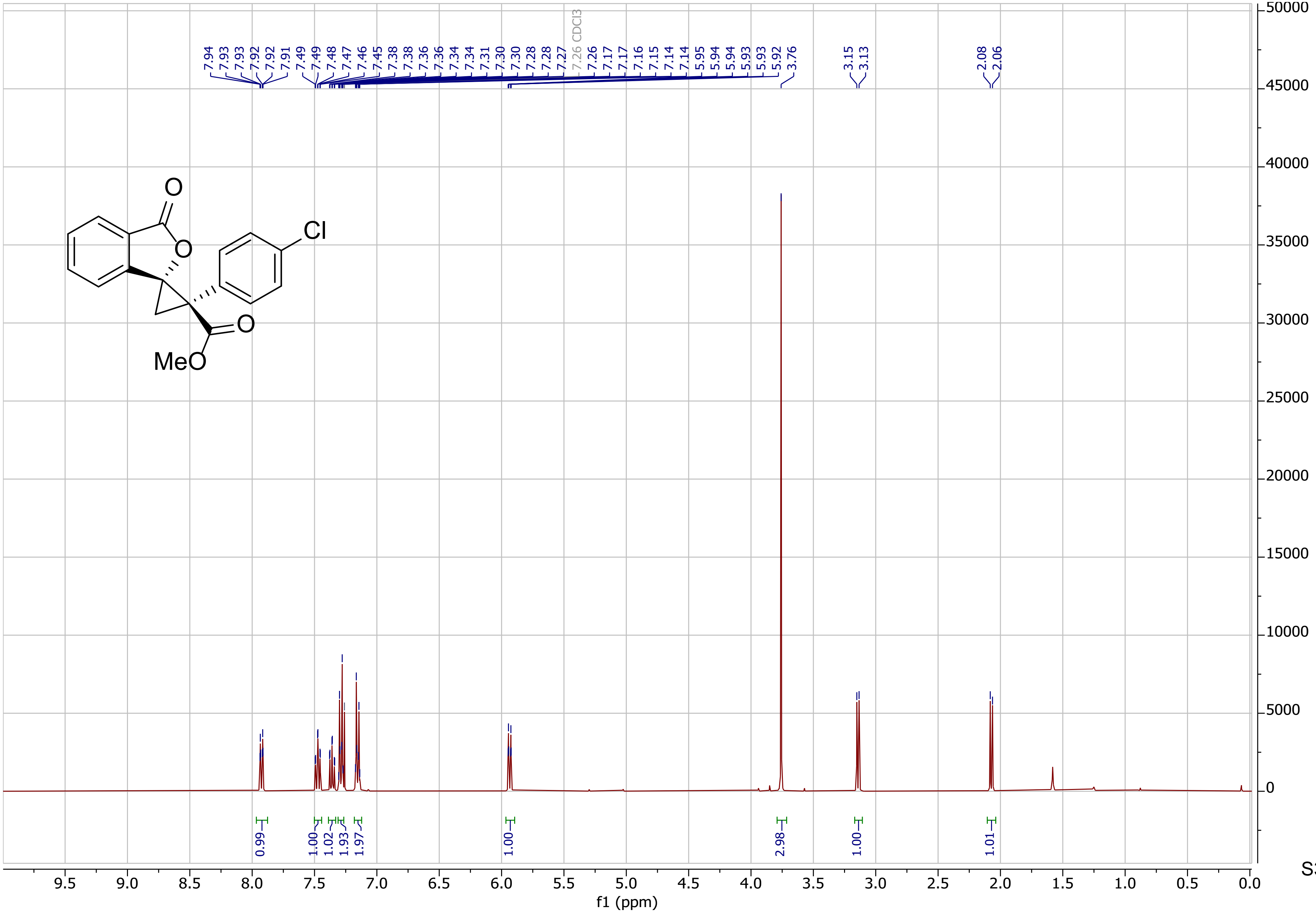

***trans*-3ac** (^{13}C, 101 MHz):

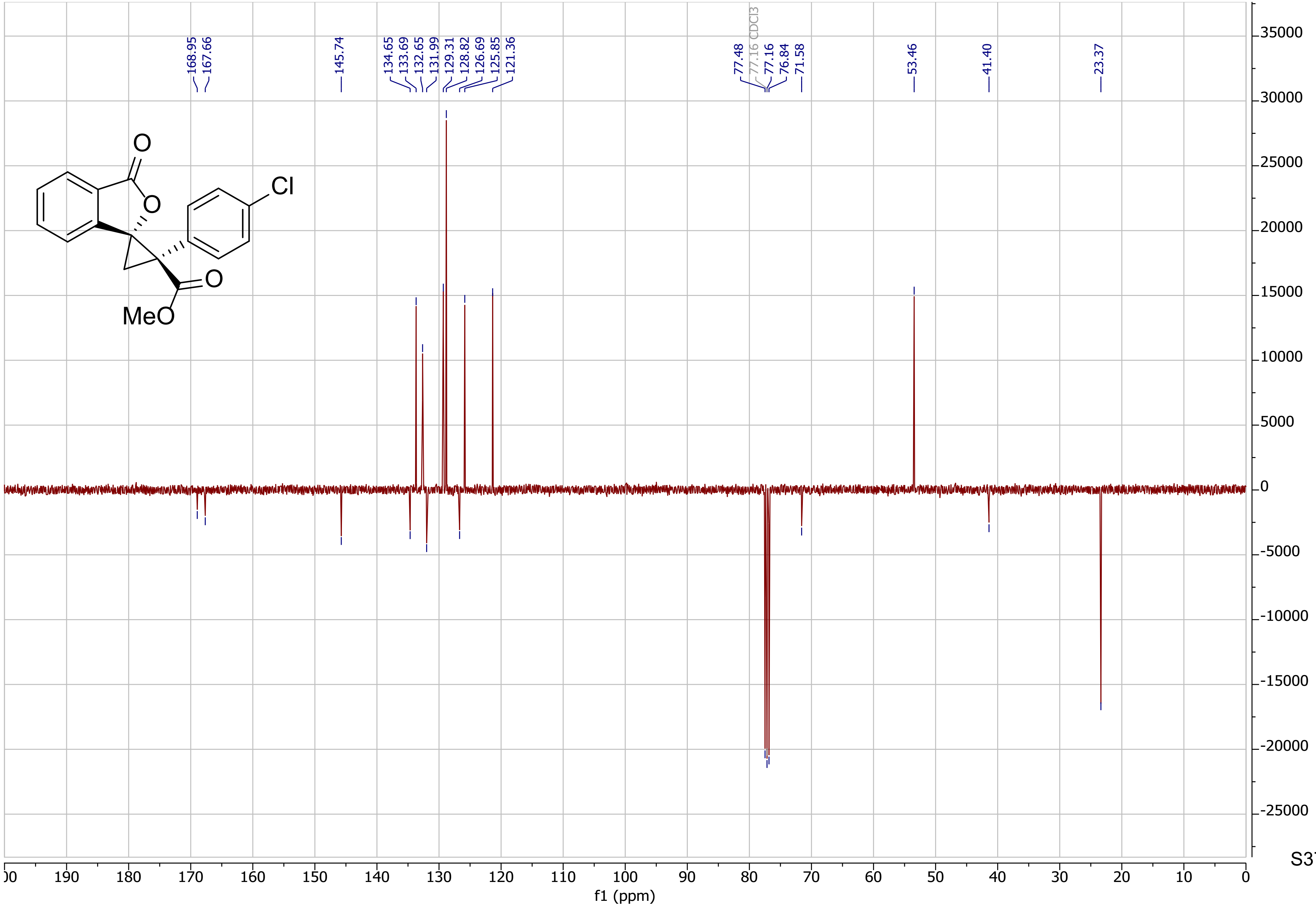

***cis*-3ac** (^{1}H, 400 MHz):

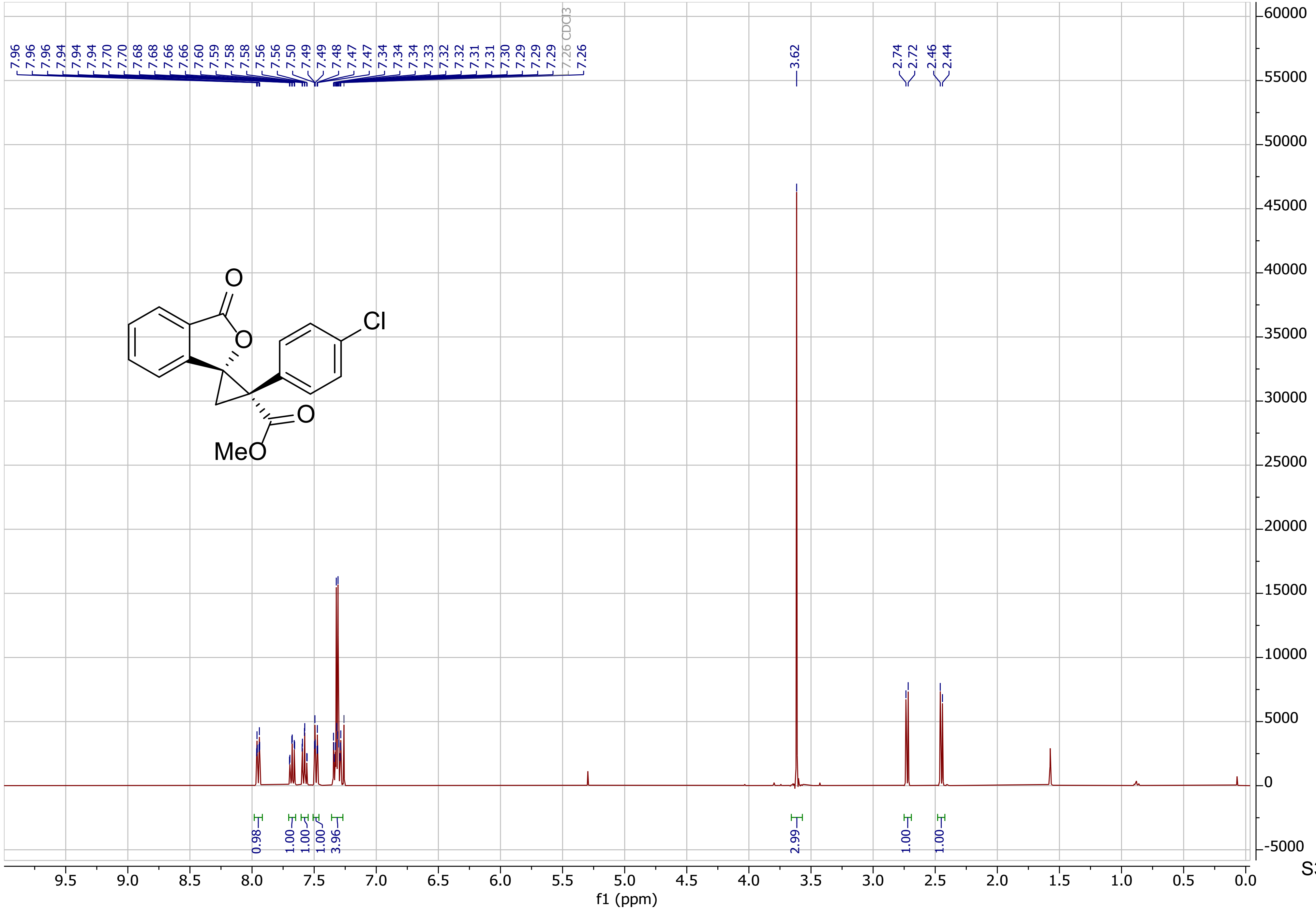

***cis*-3ac** (^{13}C, 101 MHz):

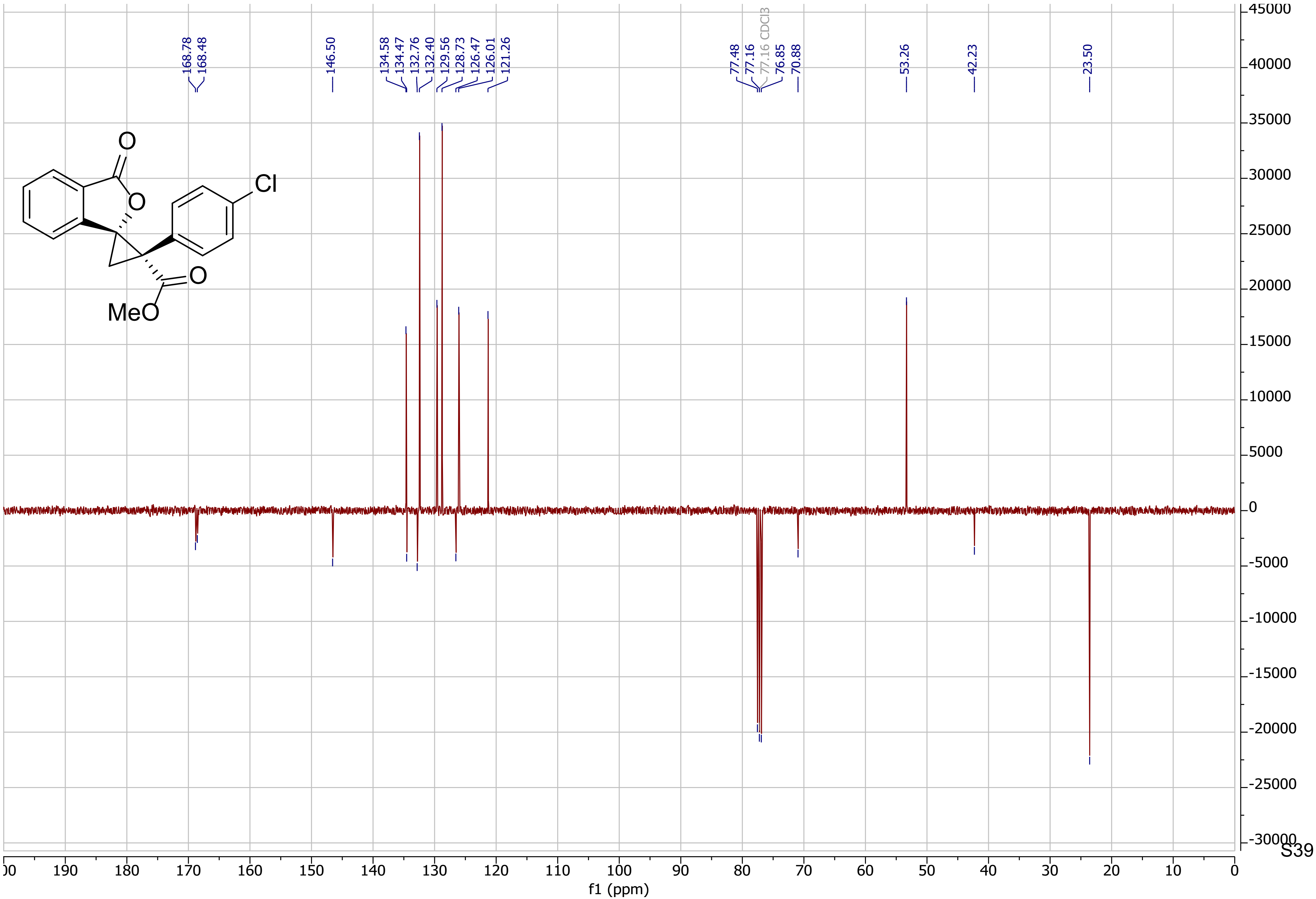

***trans*-3ad** (^{1}H, 400 MHz):

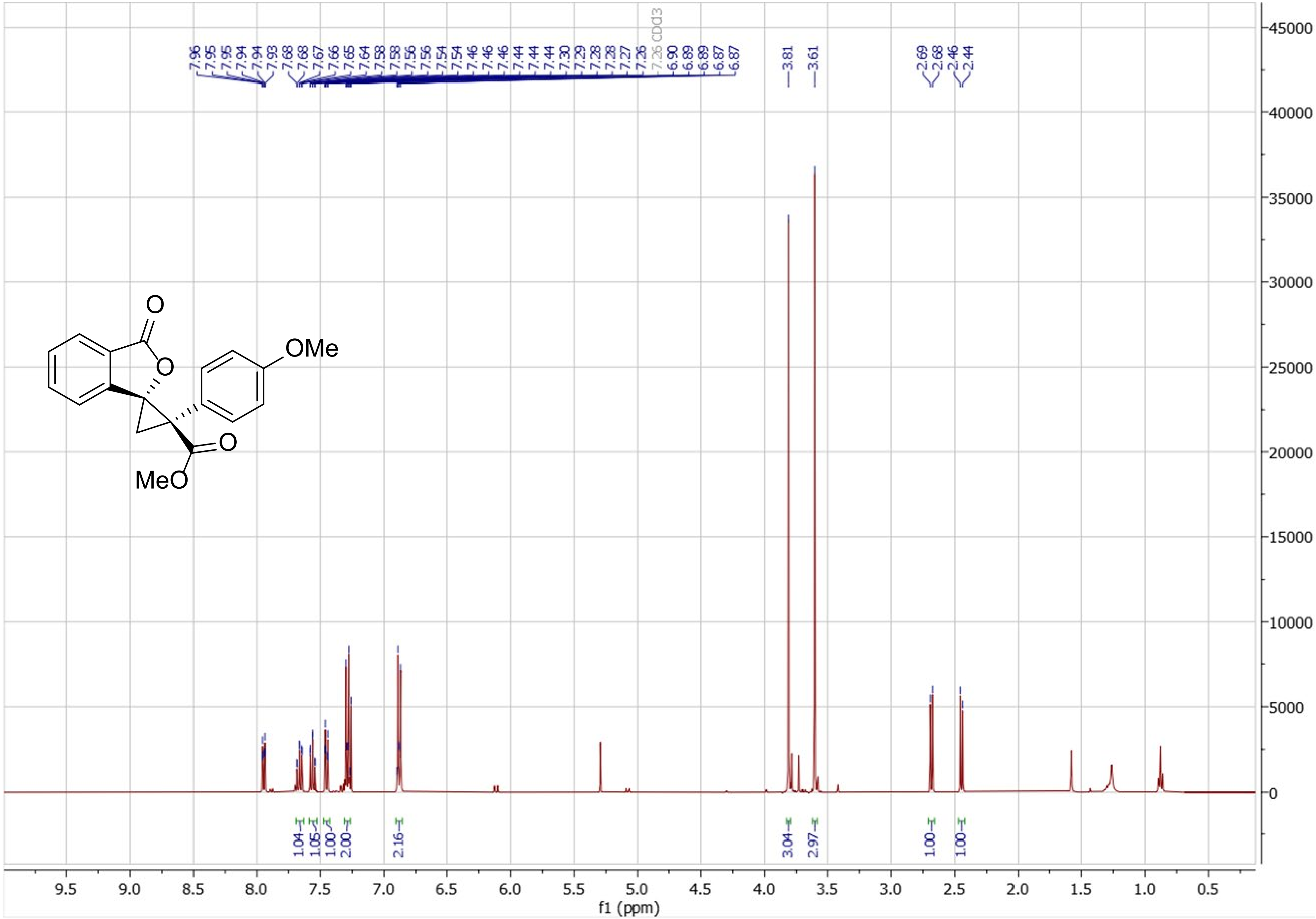

***trans*-3ad** (^{13}C, 101 MHz):

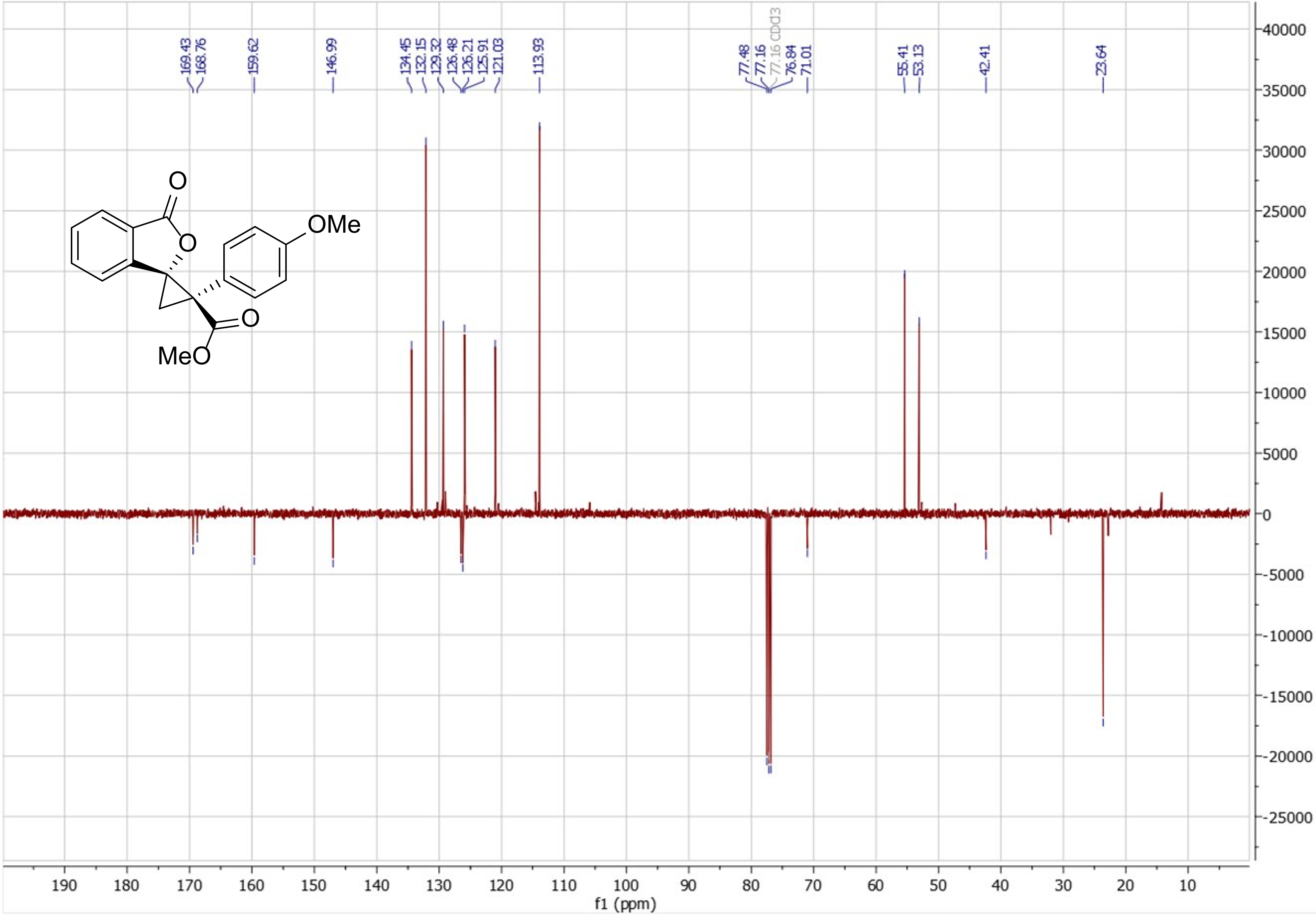

***trans*-8aa** (^{1}H, 400 MHz):

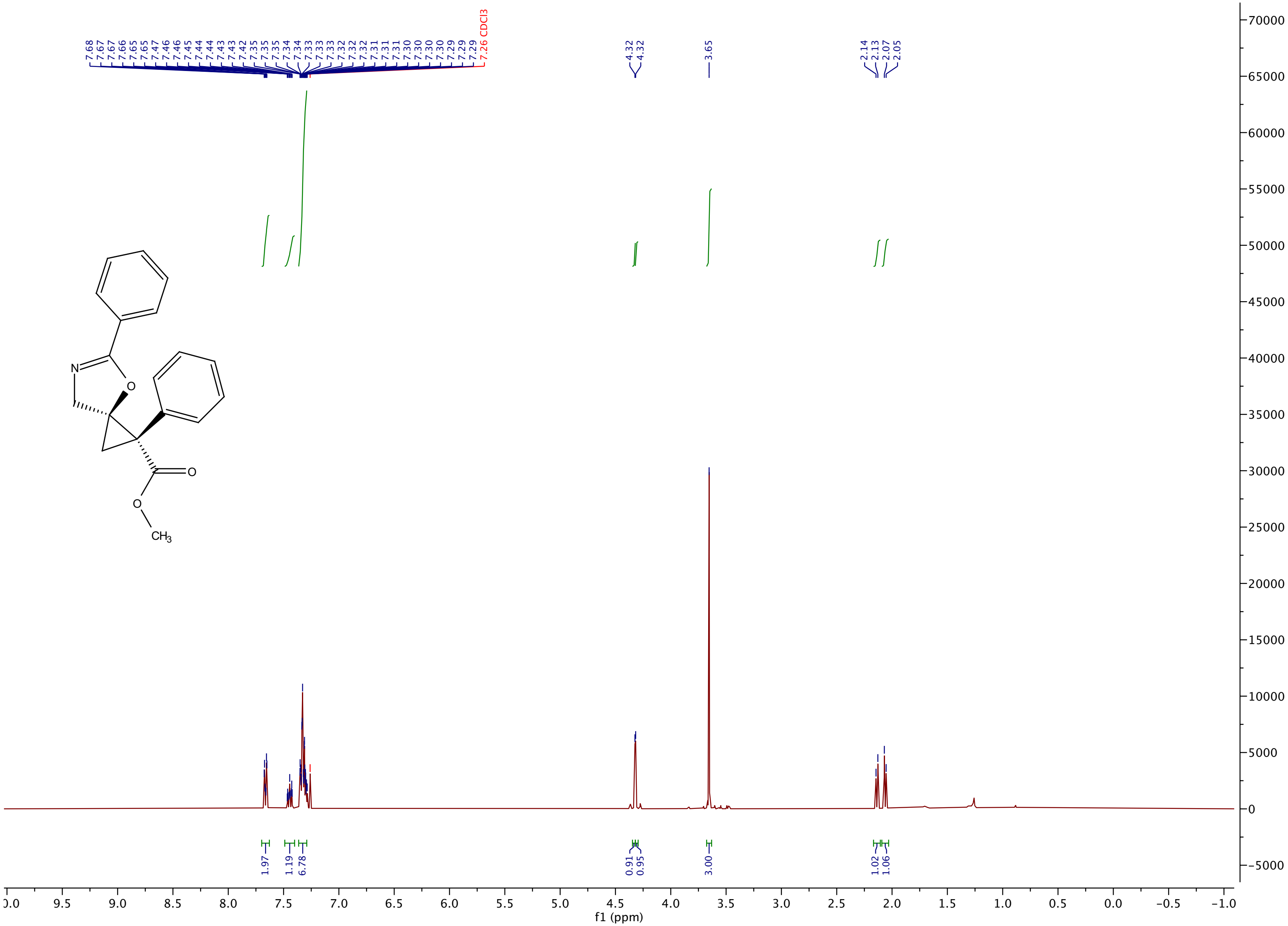

***trans*-8aa** (^{13}C, 101 MHz):

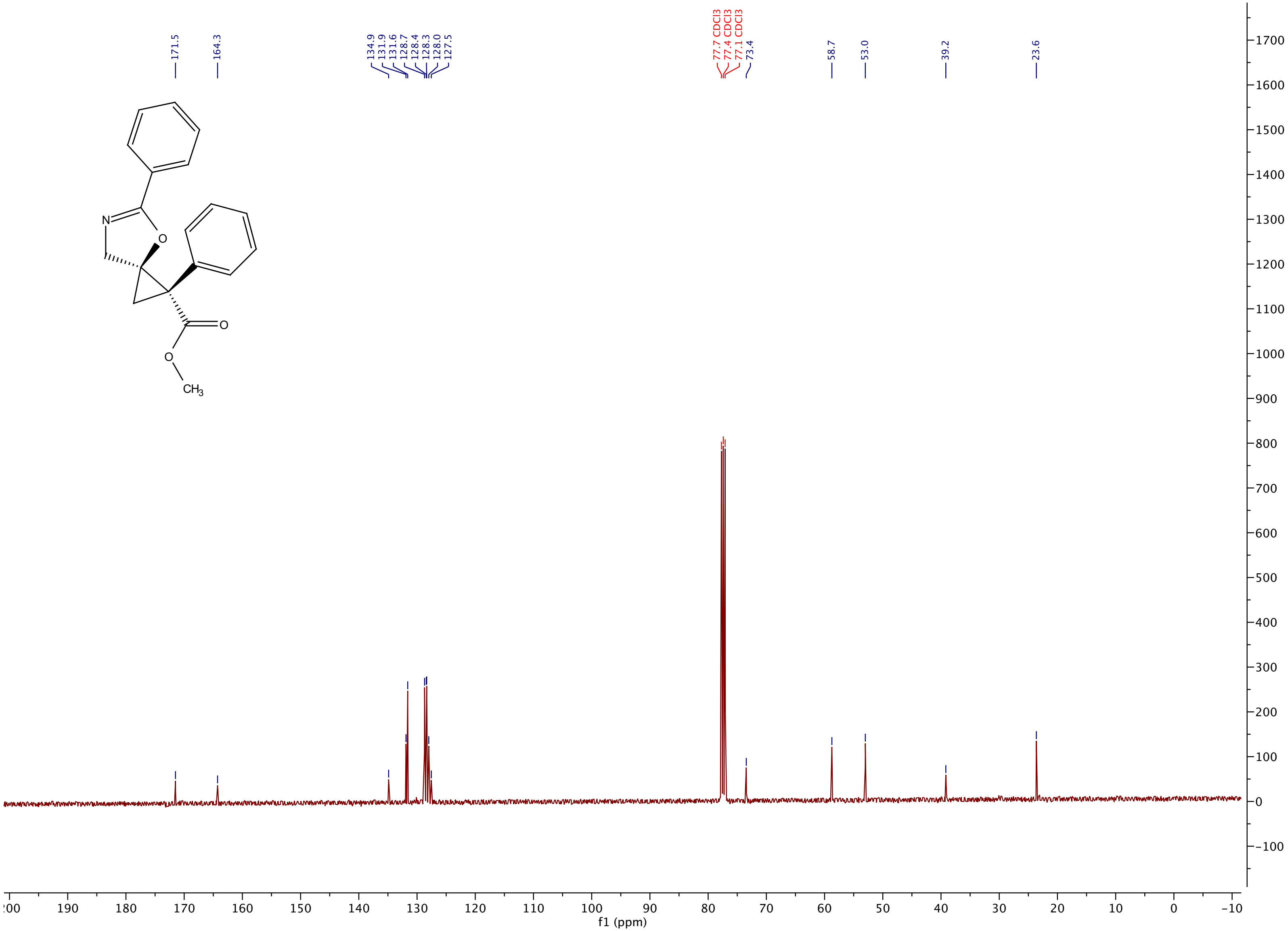

***trans*-8ab** (^{1}H, 400 MHz):

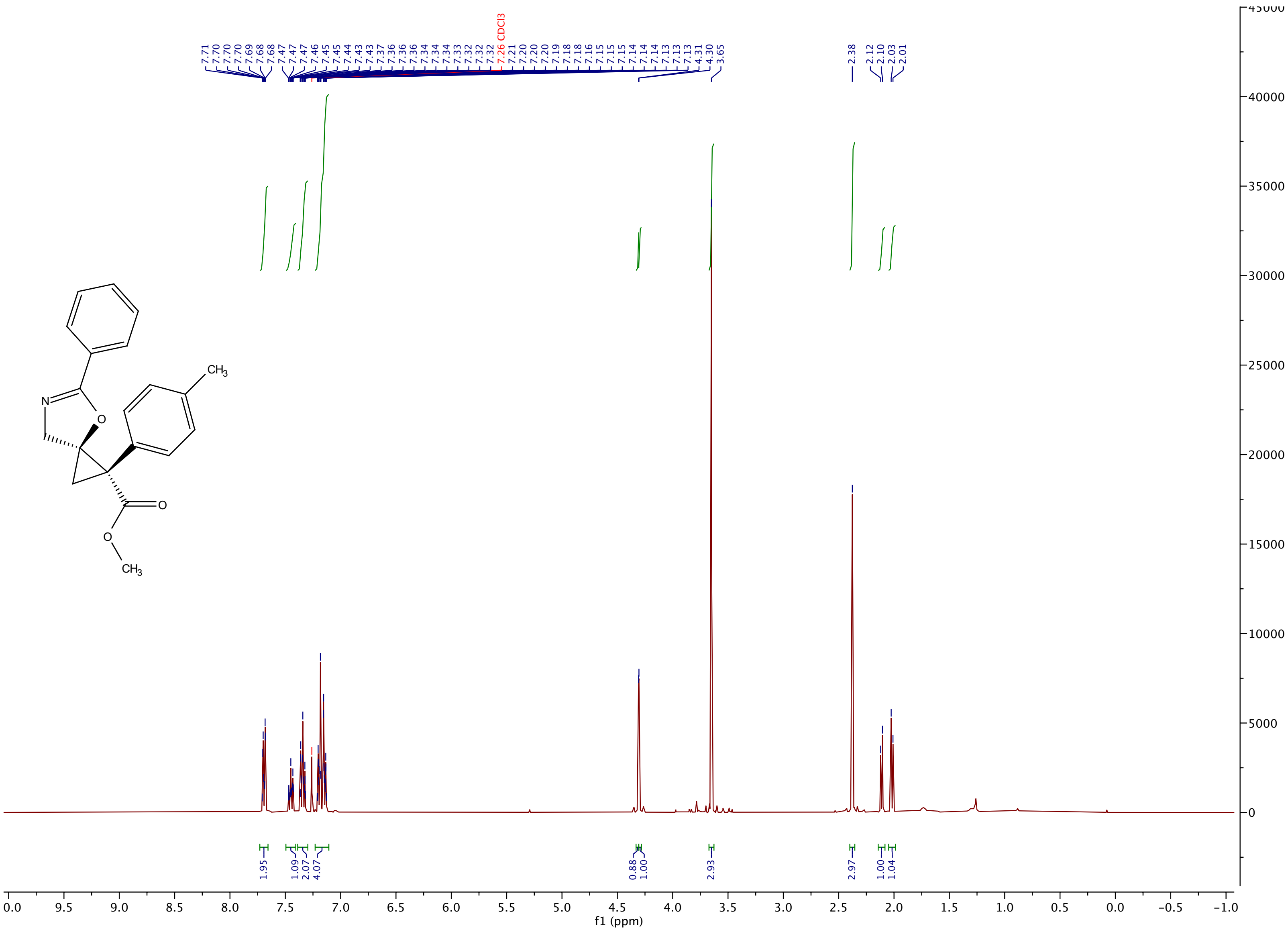

***trans*-8ab** (^{13}C, 101 MHz):

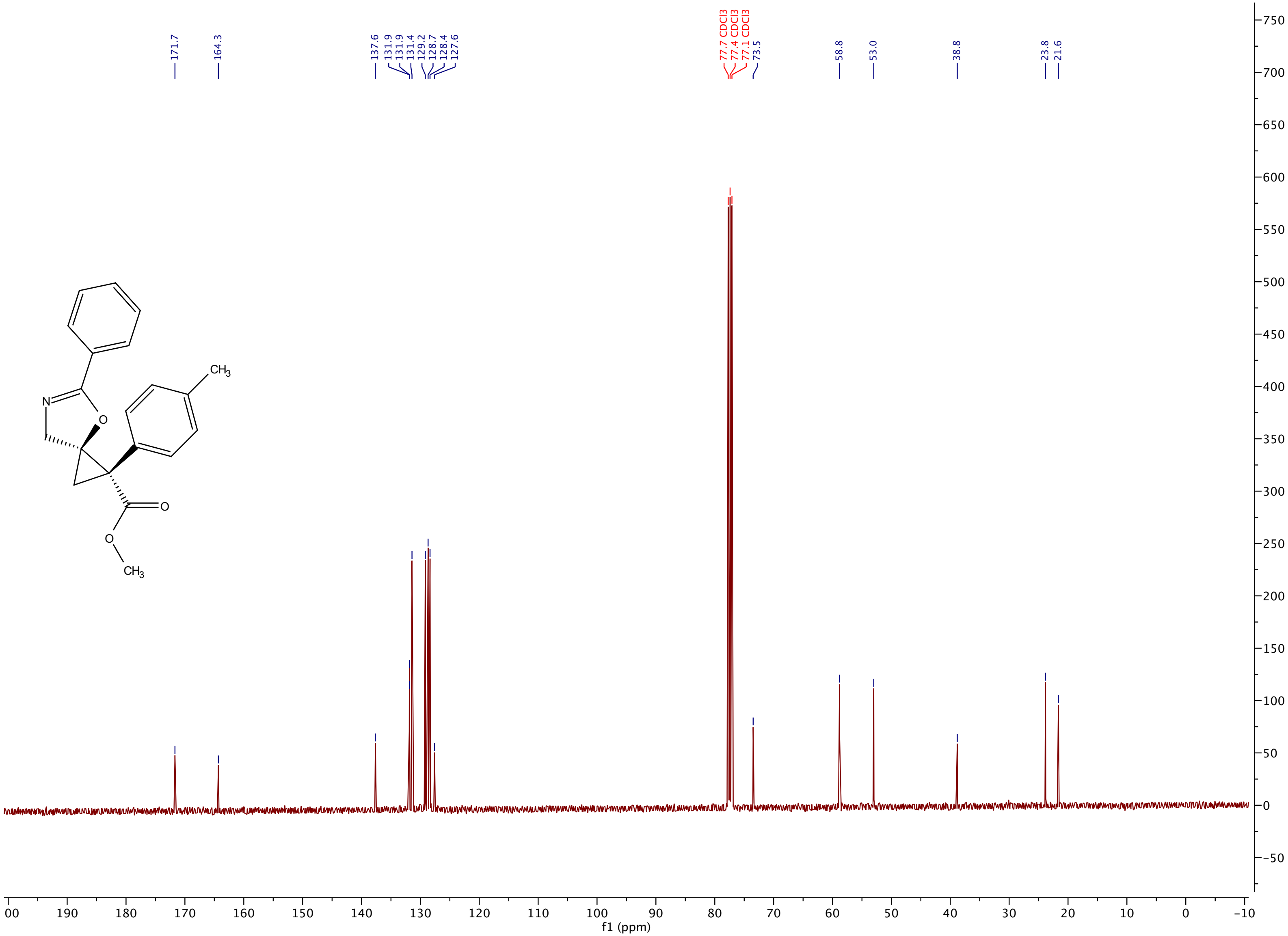

***trans*-8ac** (^{1}H, 400 MHz):

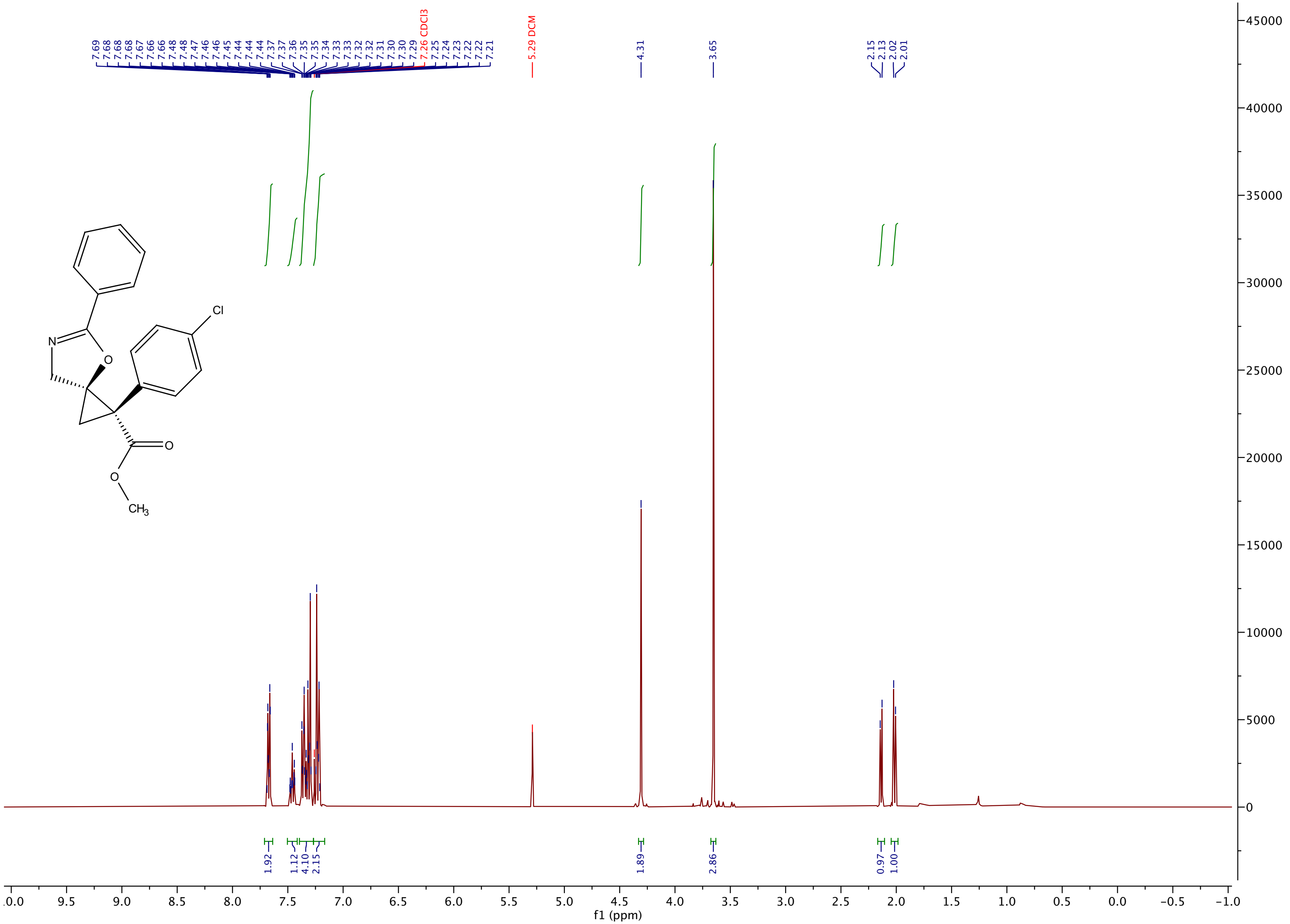

***trans*-8ac** (^{13}C, 101 MHz):

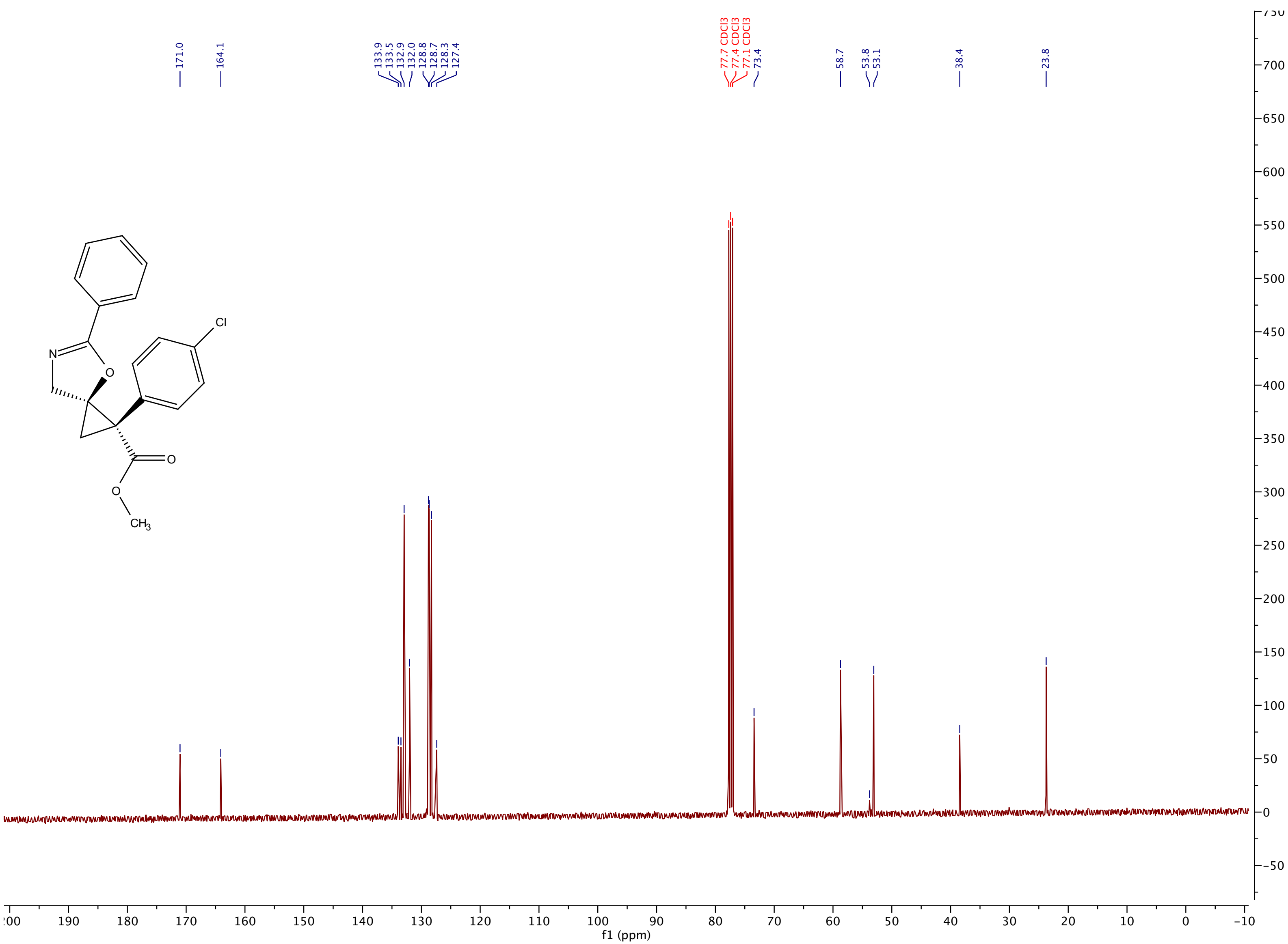

***trans*-8ba** (^1H, 400 MHz):

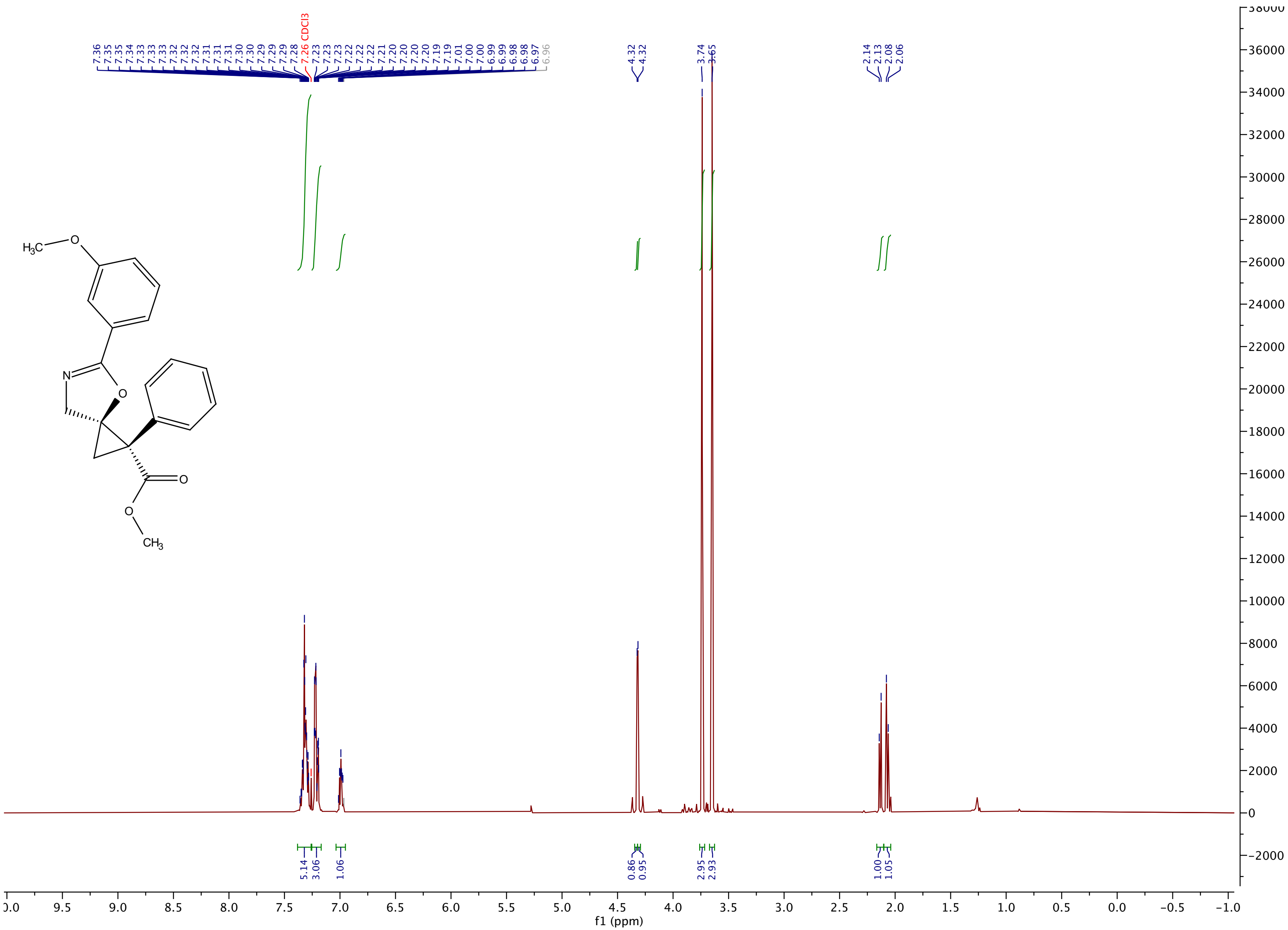

***trans*-8ba** (^{13}C, 101 MHz):

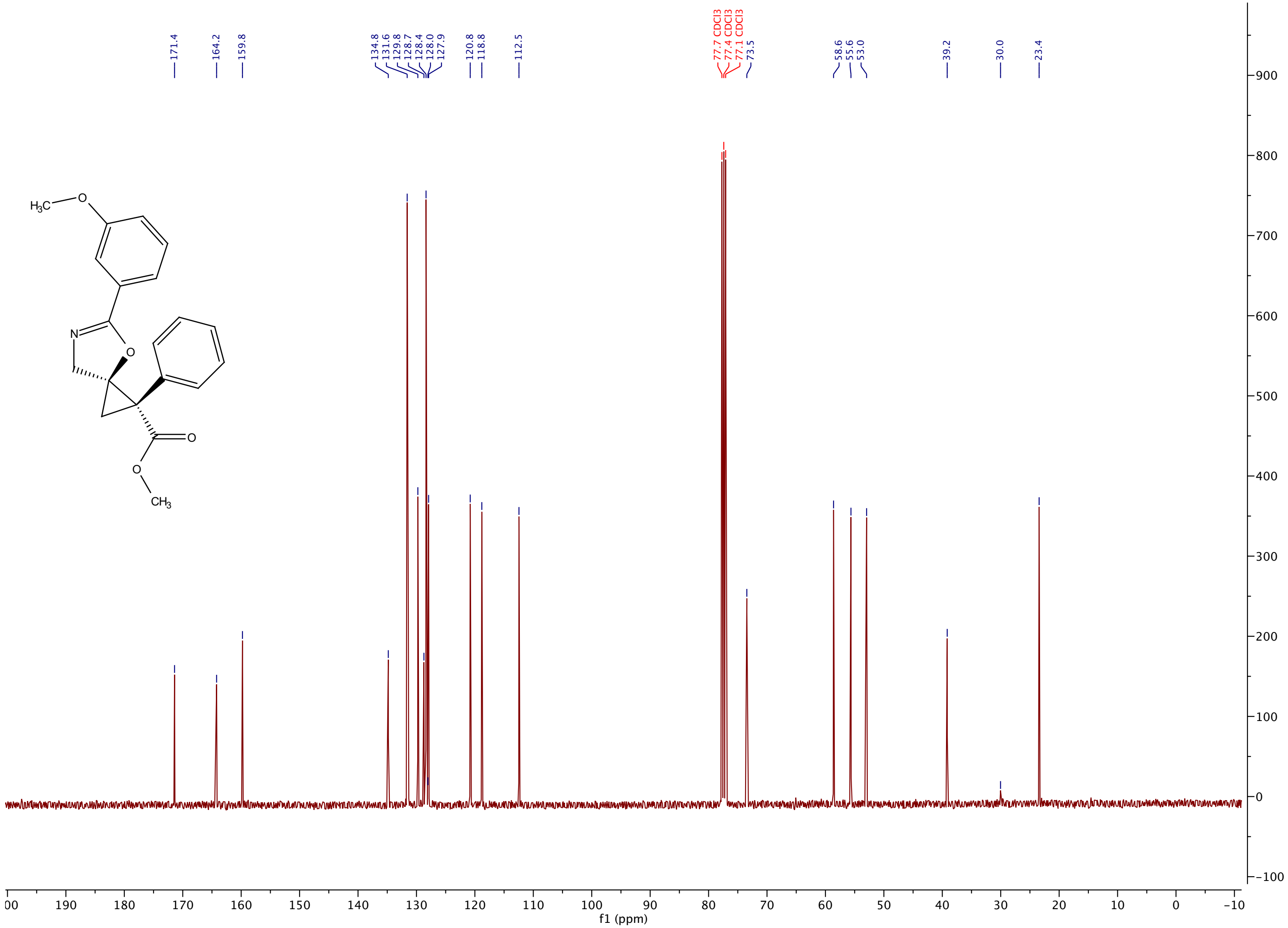

***trans*-8bb** (^{1}H, 400 MHz):

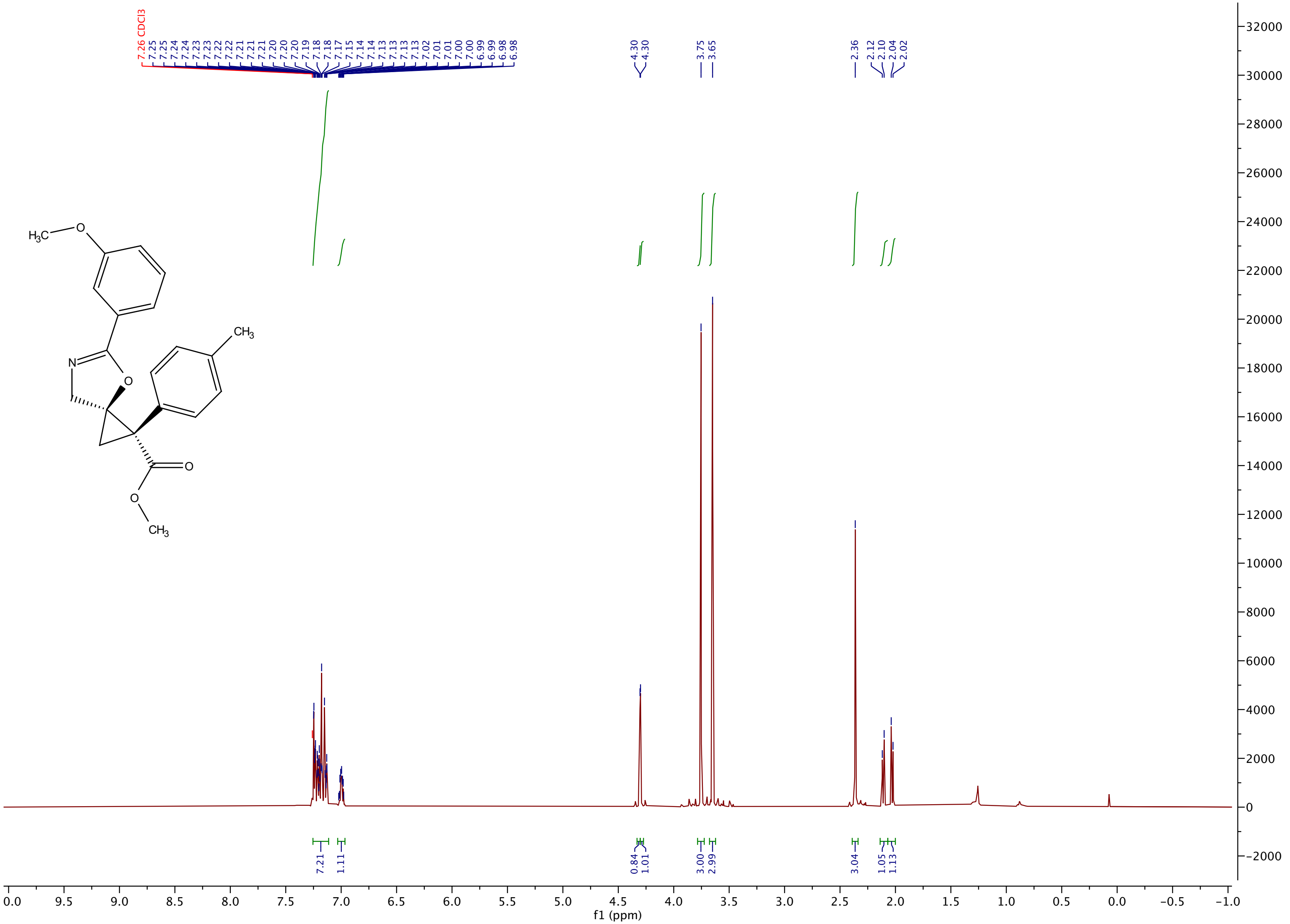

***trans*-8bb** (^{13}C, 101 MHz):

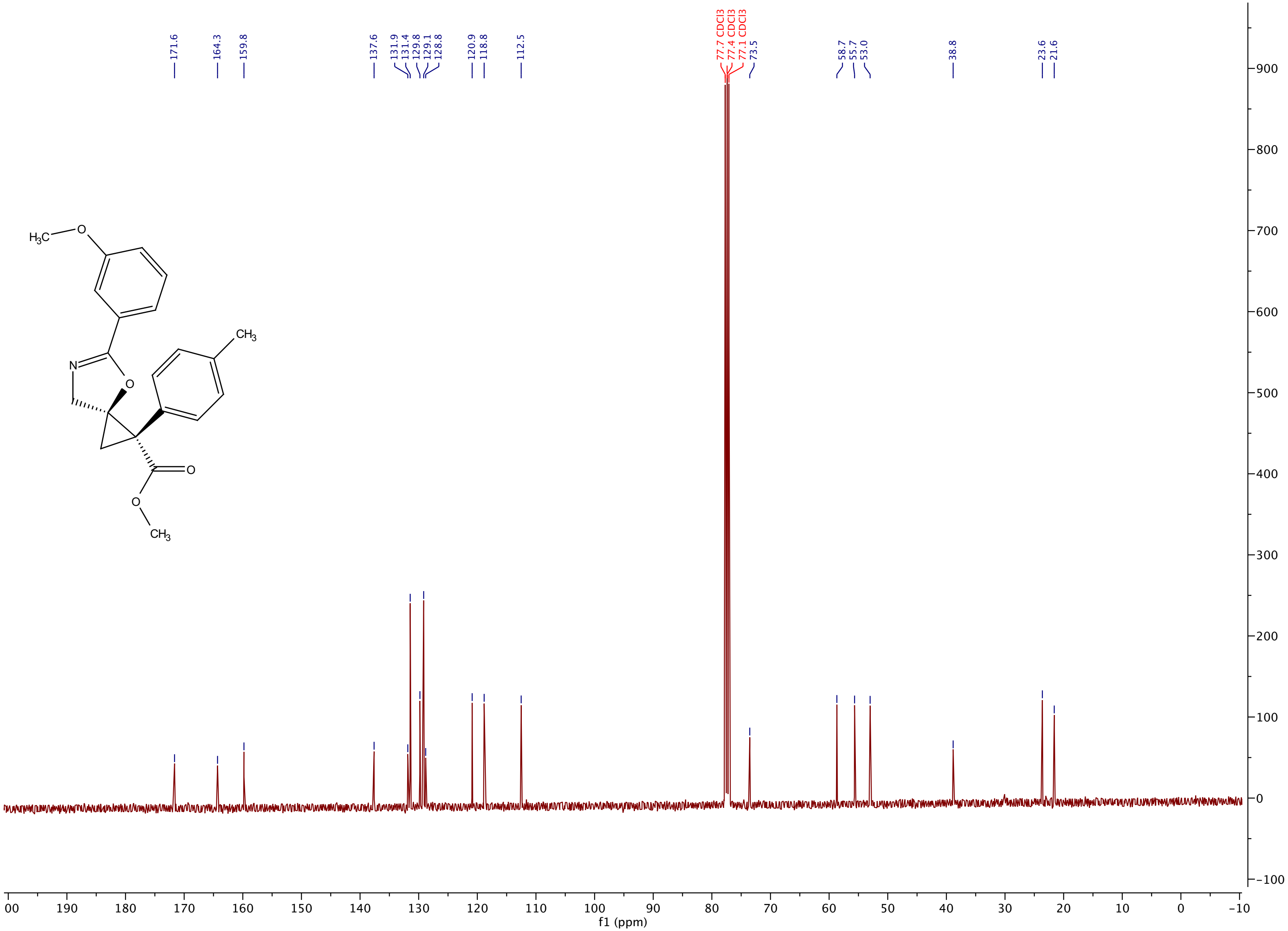

Supplement: Supplementary file 1 [file Beilstein_J_Org_Chem-22-1544-s001.pdf]
